# Thermally induced neuronal plasticity in the hypothalamus mediates heat tolerance

**DOI:** 10.1038/s41593-024-01830-0

**Published:** 2024-12-09

**Authors:** Wojciech Ambroziak, Sara Nencini, Jörg Pohle, Kristina Zuza, Gabriela Pino, Sofia Lundh, Carolina Araujo-Sousa, Larissa I. L. Goetz, Katrin Schrenk-Siemens, Gokul Manoj, Mildred A. Herrera, Claudio Acuna, Jan Siemens

**Affiliations:** 1 Institute of Pharmacology, Heidelberg University, Heidelberg, Germany; 2 Chica and Heinz Schaller Foundation, Institute of Anatomy and Cell Biology, Heidelberg University, Heidelberg, Germany; 3 Department of Pathology and Imaging, Global Drug Discovery, Novo Nordisk A/S, Måløv, Denmark; 4 Molecular Medicine Partnership Unit, European Molecular Biology Laboratory (EMBL), Heidelberg, Germany; 5 Collaboration for joint PhD degree between EMBL and Heidelberg University, Faculty of Biosciences, Heidelberg, Germany; 6 Present Address: Department of Translational Disease Understanding, Grünenthal GmbH, Aachen, Germany; 7 Present Address: Istituto Italiano di Tecnologia, Genoa, Italy

**Keywords:** Neuroscience, Physiology

## Abstract

Heat acclimation is an adaptive process that improves physiological performance and supports survival in the face of increasing environmental temperatures, but the underlying mechanisms are not well understood. Here we identified a discrete group of neurons in the mouse hypothalamic preoptic area (POA) that rheostatically increase their activity over the course of heat acclimation, a property required for mice to become heat tolerant. In non-acclimated mice, peripheral thermoafferent pathways via the parabrachial nucleus activate POA neurons and mediate acute heat-defense mechanisms. However, long-term heat exposure promotes the POA neurons to gain intrinsically warm-sensitive activity, independent of thermoafferent parabrachial input. This newly gained cell-autonomous warm sensitivity is required to recruit peripheral heat tolerance mechanisms in acclimated animals. This pacemaker-like, warm-sensitive activity is driven by a combination of increased sodium leak current and enhanced utilization of the Na_V_1.3 ion channel. We propose that this salient neuronal plasticity mechanism adaptively drives acclimation to promote heat tolerance.

## Abstract (web summary)

Ambroziak, Nencini, Pohle and colleagues identify a slowly emerging plasticity mechanism in a discrete set of hypothalamic preoptic neurons that is triggered by long-term heat exposure and that drives thermal acclimation to promote heat tolerance in mice.

## Main

Prolonged exposure to hot (but nonlethal) temperatures enhances thermoregulatory responses in peripheral organ systems to rheostatically maintain body temperature within physiological limits, an adaptive phenomenon commonly referred to as heat acclimation. It has been proposed that the central nervous system regulates these adaptive changes^1–4^.

Although hypothalamic thermoregulatory pathways orchestrating long-term acclimation and heat tolerance are unknown, several hypothalamic cell populations have been described that mediate acute heat loss responses. These neurons reside in the rostral part of the hypothalamic preoptic area (POA) with the median preoptic nucleus (MnPO) at its center, an area that from here on we refer to as the anterior ventromedial preoptic area (VMPO). A subset of VMPO neurons has been shown to respond to acute heat exposure and, in accordance with their predicted homeostatic function, acute optogenetic and chemogenetic stimulation of these—largely glutamatergic—neurons triggers prompt heat loss responses and body cooling^5–12^. However, it is not known whether POA neurons also control long-lasting rheostatic adaptations, to promote heat tolerance as a consequence of acclimation.

In the present study, we tested the hypothesis that long-term heat exposure during acclimation triggers plastic changes in the hypothalamic thermoregulatory area to regulate heat tolerance in mice.

## Results

### VMPO^LepR^ neurons gain warm sensitivity on heat acclimation

Acute exposure to hot environmental temperatures activates a subset of VMPO neurons to express the activity marker cFos^5,9,10,13–17^. We hypothesized that long-term heat exposure would alter the activity profile of these VMPO warm-responsive neurons (VMPO^WRN^), based on the premise that long-lasting thermoafferent input could induce plastic changes and cellular adaptation.

**Extended Data Fig. 1:**
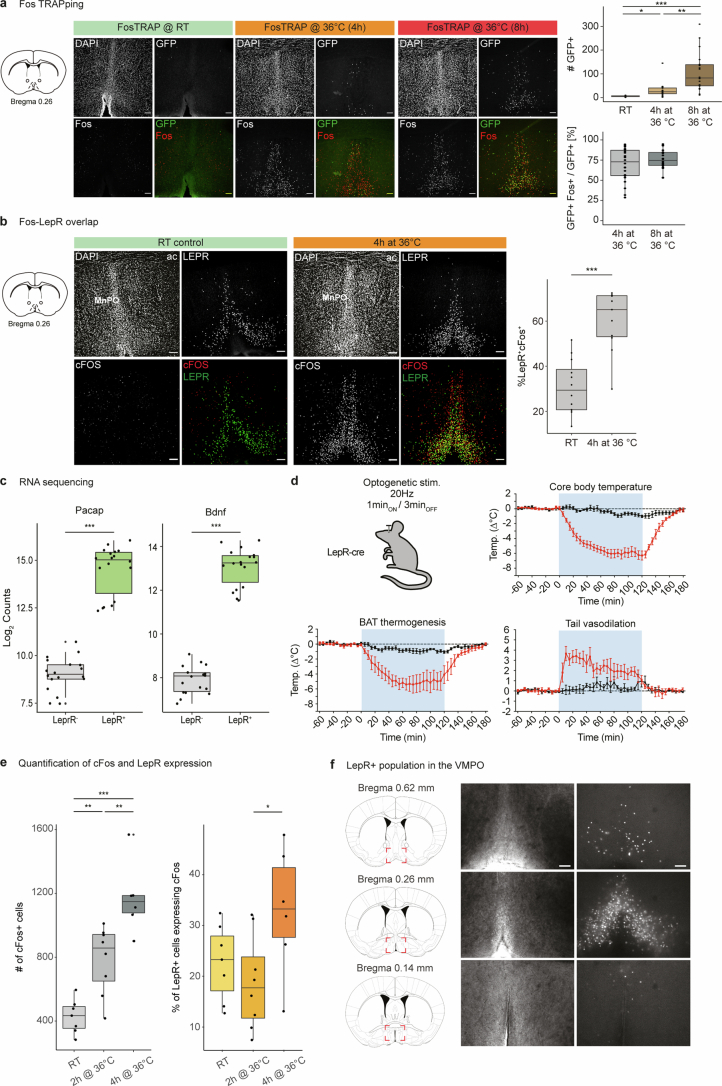
VMPO neurons responding with a delay to a heat stimulus overlap with LepR-positive neurons and are receptive to becoming activated by heat acclimation. **a**, Left: Brain sections revealing the VMPO of FosTRAP2;HTB mice that received z-4-hydroxytamoxifen (4-OHT; 50mg/kg i.p.) at room temperature (TRAP@RT) and after 2 hours of a 4-hour (TRAP@36 °C (4h)) or 8-hour (TRAP@36 °C (8h)) exposure to 36 °C,. Scale bar: 100 μm. Right top: Increasing the time of exposure to 36 °C increases the number of TRAPped GFP-positive cells in the VMPO. Kruskal-Wallis test, P <0.0001; Dunn’s multiple comparisons test, *P = 0.0110 (TRAP@RT: TRAP@36˚C (4h)), ***P < 0.0001 (TRAP@RT : TRAP@36˚C (8h)), **P = 0.0047 (TRAP@36˚C (4h) : TRAP@36˚C (8h)). n = 7 sections / 3 animals for TRAP@RT, n = 22/5 for TRAP@36˚C (4h) and n = 18/4 for TRAP@36˚C (8h). Right bottom: TRAPped neurons highly overlapped with Fos protein in animals exposed to warmth.; n = 22/5 for TRAP@36˚C (4h) and n = 18/4 for TRAP@36˚C (8h). **b**, Warm-stimulated (4 hours) and RT control brain sections of LepR-Cre;HTB mice show the rostral POA and MnPO, stained with GFP (LepR expression) and cFos. The overlap of LepR and cFos due to warm temperature exposure is quantified in the right panel graph (% of LepR-positive neurons, from 11 and 10 sections of N = 2 animals for RT and 36 °C, respectively; two-tailed T-test, ***P < 0.0001; scale bars: 100 μm). **c**, Expression of Pacap (Adcyap) and Bdnf transcripts assessed by bulk mRNA sequencing of FACS sorted LepR^+^ and LepR^-^ cells obtained from POA tissue isolated from LepR-Cre;HTB mice. DWilcoxon test, ***P < 0.0001 (LepR^+^ : LepR^-^). n=18/3 (samples/mice); each data point represents expression of the respective gene in each sample plotted as a log2 normalized value. **d**, Core body temperature, BAT and tail temperature of ChR2-expressing and control mice before, during (blue shading) and after blue light stimulation (20 Hz, 10 msec pulses, 1min ON/3min OFF). n=4 mice per group. Data represent mean ± s.e.m. **e**, Right: Quantification of the number of neurons expressing cFos in VMPO of LepR-Cre;HTB kept at room temperature (RT), and stimulated at 36 °C and displayed as % of all cFos-positive neurons. Kruskal-Wallis test, P < 0.0001; Dunn’s multiple comparisons test, **P = 0.0047 (RT : 2h @ 36 °C), ***P < 0.0001 (RT : 4h @ 36 °C), **P = 0.0034 (2h @ 36 °C : 4h @ 36 °C). n = 7 sections / 2 animals for RT, 8 sections / 3 animals for 4h @ 36 °C, and 6 sections / 2 animals for 8h @ 36 °C. Left: Quantification of the absolute number cFos-positive cells. Kruskal-Wallis test, P = 0.0442; Dunn’s multiple comparisons test, P = 0.7224 (RT : 2h @ 36^C), P = 0.1709 (RT : 4h @ 36 °C), *P = 0.0381 (2h @ 36 °C : 4h @ 36 °C). **f**, Extent of VMPO^LepR^ population. Representative images of 250 µm acute slices from LepR-Cre;HTB mice used for *ex vivo* electrophysiological experiments. Scale bar: 100 μm. Boxplots show median and interquartile range.

To assess whether exposure to warm or hot ambient temperatures (36 °C) over an extended time period would change VMPO^WRN^ neuron activity, we used a cFos-based genetic mouse model, the so-called FosTRAP2 mouse line, that allows unbiased labeling of activated neurons^18^. We captured VMPO^WRN^ neurons by exposing FosTRAP2 mice to 36 °C for 4 or 8 h. The pattern of ‘warm-TRAPped’ neurons, visualized by the expression of nuclear green fluorescent protein (nGFP) under the control of the FosTRAP2 mice (FosTRAP2;HTB), recapitulated the previously described cFos expression pattern of VMPO^WRN^ neurons (Extended Data Fig. 1a), demonstrating that FosTRAP2;HTB mice allow permanent labeling of warm-responsive neurons (WRNs). Moreover, longer heat exposure (8 versus 4 h) resulted in increased TRAPping of neurons, suggesting that progressively more neurons within the preoptic network are recruited upon longer heat exposure (Extended Data Fig. 1a).

**Fig. 1:**
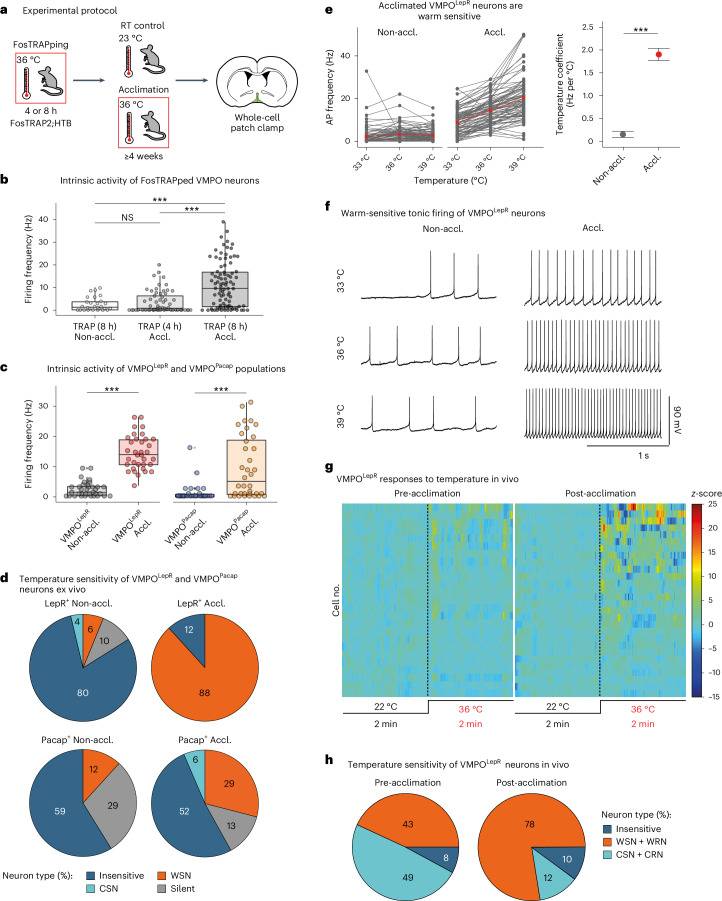
Heat acclimation increases warm-sensitive tonic AP firing of VMPO^LepR^ neurons. **a**, FosTRAPping and acclimation protocol. **b**, Spontaneous AP frequency in neurons of short (4 h) and long (8 h) warm-TRAPped mice. Neuronal activity was recoded at the 36 °C bath temperature: one-way ANOVA: *P* < 0.0001; Tukey’s multiple-comparison test: *P* = 0.8364 (TRAP (8 h) non-acclimated (Non-accl.), TRAP (4 h) acclimated (Accl.)); ^***^P < 0.0001 (TRAP (8 h) Non-accl., TRAP (8 h) Accl.); ^***^P < 0.0001 (TRAP (4 h) Accl., TRAP (8 h) Accl.) (*n* = 28/3 (TRAP (8 h) Non-accl.); *n* = 22/2 (TRAP (4 h) Accl.) and *n* = 33/3 (TRAP (8 h) Accl.)). **c**, AP firing frequency in non-acclimated (*n* = 35/6) versus acclimated (*n* = 35/6) VMPO^LepR^ and VMPO^Pacap^ neurons (*n* = 30/3 for non-acclimated and *n* = 37/3 for acclimated). Neuronal activity recoded at 36 °C bath temperature. Unpaired two-tailed Student’s *t*-test: ^***^P < 0.0001 (VMPO^LepR^ and VMPO^Pacap^ neurons). **d**, Distribution of temperature-insensitive, CSN (≤−0.6 Hz per °C), WSN (≥0.75 Hz per °C) and silent neurons in VMPO^LepR^ (*n* = 81/9 non-acclimated, *n* = 85/10 acclimated) and VMPO^P^^acap^ (*n* = 17/3 non-acclimated, *n* = 31/3 acclimated) neurons, recorded at 33 °C, 36 °C and 39 °C. **e**, Left: firing frequencies of non-acclimated (*n* = 81/9) and acclimated (*n* = 85/10) VMPO^LepR^ neurons recorded at three bath temperatures as indicated. Individual cells plotted in gray and red points represent group averages. Right: temperature coefficient (Hz per °C; mean ± s.e.m.) comparison between the non-acclimated and acclimated VMPO^LepR^ neurons. Unpaired, two-tailed Student’s *t*-test: ^***^P < 0.0001. **f**, Example traces of a non-acclimated and an acclimated VMPO^LepR^ neuron. **g**, Heatmaps displaying in vivo single-cell VMPO^LepR^ neuron responses at 22 °C and 36 °C, before (left) and after (right) 30 d of heat acclimation. **h**, Pie charts showing fractions of VMPO^LepR^ neurons increasing (WSN + WRN), decreasing (CRN + CRN) or not changing (insensitive) activity when ambient temperature was increased from 22 °C to 36 °C, before and after heat acclimation. Number of cells pre-acclimation: WSN + WRN, 22; CRN + CRN, 25; insensitive, 4; post-acclimation: WSN + WRN, 38; CRN, 6; insensitive, 5; *N* = 4 mice. Ex vivo recordings performed with fast synaptic transmission blockade. Box plots represent the median and IQR (Extended Data Figs. 2 and 3). CSN, cold-sensitive neuron.

Next, we warm-TRAPped FosTRAP2;HTB animals for either 4 or 8 h and subsequently acclimated them at 36 °C for ≥4 weeks, a time period required to reach full heat acclimation in rodents^19^. Finally, we prepared acute brain slices for electrophysiological recordings (Fig. 1a).

Strikingly, longer-TRAPped neurons—but not shorter-TRAPped neurons—showed increased tonic activity (spontaneous action potential (AP) firing) when FosTRAP2;HTB mice were acclimated (Fig. 1b).

Leptin receptor- (LepR-) and PACAP/BDNF (brain-derived neurotrophic factor)-expressing VMPO (VMPO^LepR^ and VMPO^Pacap^) neurons have been found to partially overlap with the VMPO^WRN^ neuron population, with subfractions of them coexpressing the activity marker cFos when mice are placed at warm temperatures^8,10,20^, a finding that we confirmed (Extended Data Fig. 1b,c).

Moreover, VMPO^LepR^ and VMPO^Pacap^ neurons can drive heat loss responses when activated chemogenetically and optogenetically^8,10^ (Extended Data Fig. 1d), consistent with a role in thermoregulation during heat exposure. We therefore wondered whether VMPO^LepR^ and/or VMPO^Pacap^ neurons would also change their activity profile on long-term heat acclimation. We heat acclimated animals expressing a green fluorescent reporter under the control of the leptin receptor gene (LepR-Cre;HTB) or the *PACAP* gene (PACAP;EGFP) for ≥4 weeks at 36 °C. Indeed, we also found that VMPO^Pacap^ and VMPO^LepR^ neurons increased AP firing on long-term heat acclimation, with the smaller LepR-positive population appearing to plastically transform more robustly (Fig. 1c).

We noted that 8-h warm-TRAPping labeled neurons with a greater potential to subsequently become acclimation activated compared with shorter (4-h) TRAPping (Fig. 1b). It is interesting that this result mirrored warm-induced cFos labeling of VMPO^LepR^ neurons: although native cFos expression follows an overall faster kinetic than cFos-TRAPping, a substantial fraction of cFos-positive cells coincided with VMPO^LepR^ neurons only after 4 h but not yet after 2 h (Extended Data Fig. 1e), suggesting that those VMPO neurons that slowly respond to prolonged thermal stimuli transform into acclimation-activated neurons rather than rapid responders.

**Extended Data Fig. 2:**
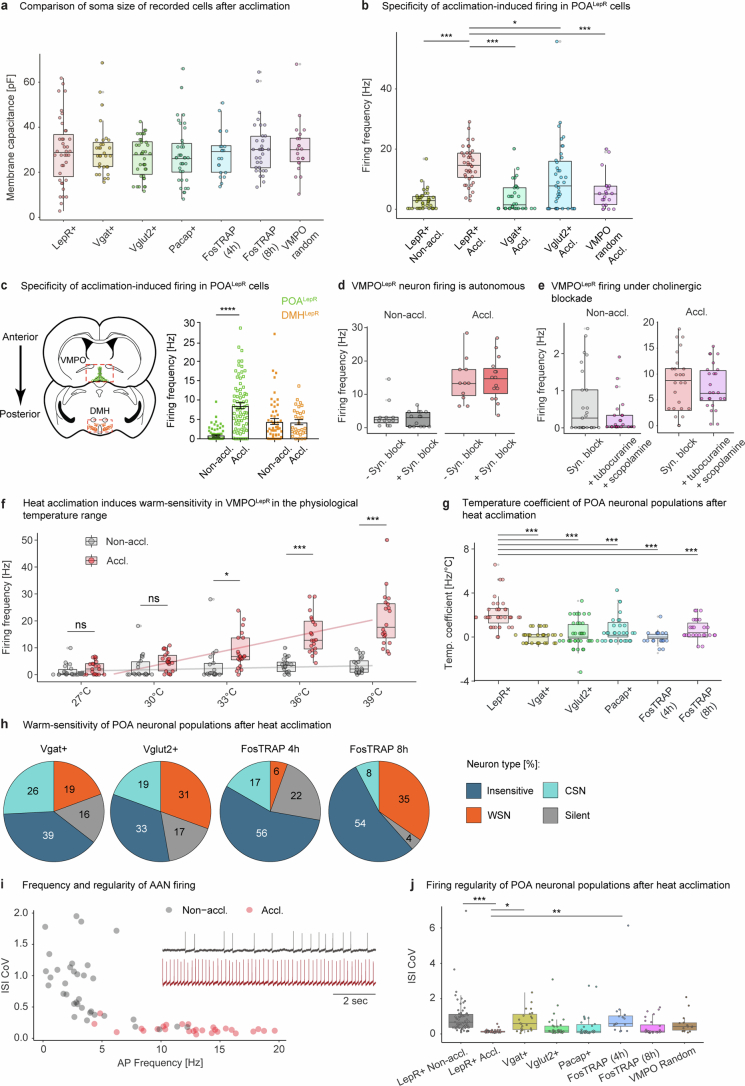
Heat acclimation-induced upregulation of tonic warm-sensitive AP firing is most robustly detected in the VMPO^LepR^ population. **a**, Comparison of membrane capacitances in acclimated neurons from various VMPO populations recorded in acute slices, selecting cells with similar soma sizes. **b**, On average, acclimated VMPO^LepR^ neurons had a significantly higher firing frequency compared to acclimated VMPO^Vgat^ neurons, acclimated VMPO^Vglut2^ neurons as well as randomly sampled VMPO acclimated neurons. Non-acclimated VMPO^LepR^ neurons plotted for reference. One-way ANOVA, P < 0.0001; Sidak’s multiple comparison test, ***P < 0.0001 (LepR+ Accl. : LepR+ Non-accl.), ***P < 0.0001 (LepR+ Accl. : Vgat+ Accl.), *P = 0.0362 (LepR+ Accl. : Vglut2+ Accl.), ***P < 0.0001 (LepR+ Accl. : VMPO random Accl.). n = 40/7 (LepR+ Accl), n = 40/6 (LepR+ Non accl), n = 31/3 (Vgat+ Accl.), n = 39/4 (Vglut2+ Accl.) and n = 21/2 (VMPO Random Accl.) cells. **c**, VMPO^LepR^ neurons (green) and DMH^LepR^ neurons (orange) were recorded in whole-cell patch clamp configuration to assess acclimation-induced AP firing frequency increases in acclimated and non-acclimated animals (n=35/5 per group). Kruskal-Wallis test (H = 46.20, d.f. = 3, P < 0.0001); Dunn’s pairwise comparisons with Bonferroni corrections (p < 0.0001 for Non-Accl. : Accl.POA LepR). No change in the average AP firing frequency was observed in DMH^LepR^ neurons. **d**, AP firing rates at 36 °C in both non-acclimated and acclimated VMPO^LepR^ neurons in *ex vivo* brain slices are comparable in the presence and absence of the fast synaptic transmission blockers (CNQX 10 µM, APV 50 µM and Gabazine 5 µM). n = 12/2 (Non-accl. –Syn. block), n = 15/3 (Non-accl. +Syn. block), n = 11/2 (Accl. –Syn. block), n = 15/3 (Accl. +Syn. block) cells. **e**, Adding acetylcholine receptor antagonists tubocurarine (10 µM) and scopolamine (10 µM) to the solution with CNQX, APV, and Gabazine slightly (but insignificantly) reduced AP firing in non-acclimated VMPOLepR neurons and did not affect AP firing in acclimated VMPOLepR cells: n = 25/5 (Non-accl. / syn. block), n = 25/4 (Non-accl. / syn. block+tubocurarine+scopolamine), n = 25/6 (Accl. / syn. block), n = 25/4 (Accl. / syn. block+tubocurarine+scopolamine). Data recorded at 33 °C. **f**, Frequencies of VMPOLepR at sub-physiological and physiological temperatures: regression analysis (grey and red lines) of non-acclimated and acclimated VMPOLepR firing rates at 33 °C, 36 °C, and 39 °C (data in main Fig. 1e). The analysis predicted that firing rates would be indistinguishable at ~29.1 °C (intersection of red and grey lines), confirmed experimentally by recording at 27 °C and 30 °C. Data partially overlapping with Fig. 1e. **g**, Warm sensitivity (measured by the temperature coefficient T_c_) of tested VMPO neuronal populations after heat acclimation. One-way ANOVA, P < 0.0001; Tukey’s multiple comparison test, ***P < 0.0001 (LepR+ : Vgat+), ***P < 0.0001 (LepR+ : Vglut2+), ***P < 0.0001 (LepR+ : Pacap+), ***P < 0.0001 (LepR+ : FosTRAP 4h), ***P < 0.0001 (LepR+ : FosTRAP 8h). n = 38/7 (LepR+), n = 31/3 (Vgat+), n = 36/4 (Vglut2+), n = 31/3 (Pacap+), n = 18/2 (FosTRAP 4h) and n = 26/3 (FosTRAP 8h) cells. **h**, Distribution of *ex vivo* recorded temperature-insensitive, cold-sensitive (CSN, temperature coefficient < −0.6 Hz/°C), warm-sensitive (WSN, temperature coefficient ≥ 0.75 Hz/°C) and silent neurons within the acclimated VMPO neuronal populations demarcated by the expression of Vgat (n = 31/3) and Vglut2 (n = 36/4) as well as ‘warm TRAPped’ neurons for either 4h (n = 18/2) or 8h (n = 26/3). Compare with Fig. 1d. **i**, Spontaneous activity pattern in representative non-acclimated and acclimated VMPO^LepR^ differs not only by frequency but also regularity of action potential firing as evidenced by the different inter spike interval coefficient of variation (ISI CoV, a measure of AP firing regularity, is the standard deviation of the interspike interval (ISI) divided the mean ISI). Analysed recordings were performed at 36 °C bath temperature. **j** Interspike interval coefficient of variation (ISI CoV) for the indicated neuronal populations obtained from heat acclimated mice. One-way ANOVA, P < 0.0001; Tukey’s multiple comparison test, ***P < 0.0001 (LepR+ Non-accl. : LepR+ Accl.), *P = 0.0215 (LepR+ Accl. : Vgat+), **P = 0.0011 (LepR+ Accl. : FosTRAP 4h). n = 63/7 (LepR+ Non-accl.), n = 57/7 (LepR+ Accl.) n = 22/3 (Vgat+), n = 31/4 (Vglut2+), n = 30/3 (Pacap+), n = 16/2 (FosTRAP 4h), n = 28/3 (FosTRAP 8h) and n = 15/2 (Random) cells. Neuronal activity was recoded in brain slices under fast synaptic transmission blockade and using ‘high-K+ aCSF’ except for panels (c) and (e) where ‘low-K+ aCSF’ and 33 °C bath temperature were used. Boxplots in (a), (b), (d), (e), (f), (g), and (j) represent median and interquartile range; data in (c) are shown as mean ± s.e.m.

To further evaluate the specificity of the observed acclimation-induced plasticity, we randomly sampled unlabeled VMPO neurons of similar size compared with VMPO^LepR^ neurons, assessed by cellular capacitance measurements (Extended Data Figs. 1f and 2a), to find that acclimation-induced plasticity is not a general phenomenon of all (randomly selected) VMPO neurons (Extended Data Fig. 2b).

Several recent studies suggest that heat loss responses are largely mediated by glutamatergic (Vglut2-positive) rather than γ-aminobutyric acid (GABA)ergic (Vgat-positive) VMPO neurons^6,9,10,21,22^. In line with these observations, we found Vglut2-positive (but not Vgat-positive) VMPO neurons to be enriched in the heat acclimation-induced population. However, their acclimation-induced response profile appeared more heterogeneous compared with VMPO^LepR^ neurons, with a considerable subset of VMPO^Vglut2^ neurons being silent or near-silent (Extended Data Fig. 2b). The observed VMPO^Vglut2^ (and VMPO^Pacap^) neuron response heterogeneity correlates with the presumed larger cell-molecular diversity of these two populations compared with the smaller VMPO^LepR^ neuron population^9^.

In contrast, cold-responsive, LepR-positive neurons residing in the dorsal medial hypothalamus (DMH^LepR^)^23–25^ did not increase their firing rates with heat acclimation (Extended Data Fig. 2c).

Importantly, in both TRAPped WRNs (Fig. 1b) and VMPO^LepR^ neurons (Extended Data Fig. 2d,e), inhibiting fast synaptic transmission did not affect the increased AP firing, indicating induction of a cell-autonomous, tonic pacemaker-like mechanism by heat acclimation.

Intriguingly, tonic activity is a characteristic feature of the so-called warm-sensitive neurons (WSNs) that increase their activity (spontaneous AP firing rate (fAP)) upon temperature (*T*_core_) increase, presumably to mount appropriate heat loss responses. Traditionally, WSNs are identified ex vivo in brain-slice preparations by monitoring their fAP while warming the temperature of the perfusion fluid^26^. However, their physiological role and significance are not fully understood, largely because specific molecular markers for this cellular population have not been found^13,15,16,27,28^.

We hypothesized that VMPO^LepR^ neurons might be the long sought-after WSNs. However, non-acclimated VMPO^LepR^ showed little to no warm sensitivity. Strikingly, heat acclimation transformed most VMPO^LepR^ neurons into robust, cell-autonomous WSNs (Fig. 1d–f).

We wondered whether acclimated and non-acclimated neurons would become indistinguishable at a bath temperature of around 29.1 °C, which was predicted by regression analysis (Fig. 1e and Extended Data Fig. 2f). Indeed, at recording temperatures ≤30 °C, the firing rates became indistinguishable (Extended Data Fig. 2f), suggesting that the decisive difference of non-acclimated versus acclimated VMPO^LepR^ neurons is their acquired warm sensitivity in the physiological temperature range (36–39 °C). Acclimation-induced warm sensitivity was lower in PACAP- and VGlut2-positive VMPO neurons (Fig. 1d and Extended Data Fig. 2g,h). Moreover, warm-sensitive tonic firing of VMPO^LepR^ neurons became highly regular as a consequence of acclimation. Again, this feature, assessed by determining the coefficient of variation of the interspike interval (ISI_Cov_), was most pronounced in acclimated VMPO^LepR^ neurons compared with any other population analyzed (Extended Data Fig. 2i,j).

Taken together, we found expression of the *LepR* gene in the VMPO to circumscribe a population of heat acclimation-activated neurons, with most VMPO^LepR^ neurons acquiring warm-sensitive pacemaker activity.

### Heat acclimation enhances warm responsiveness of VMPO^LepR^ neurons in vivo

**Extended Data Fig. 3:**
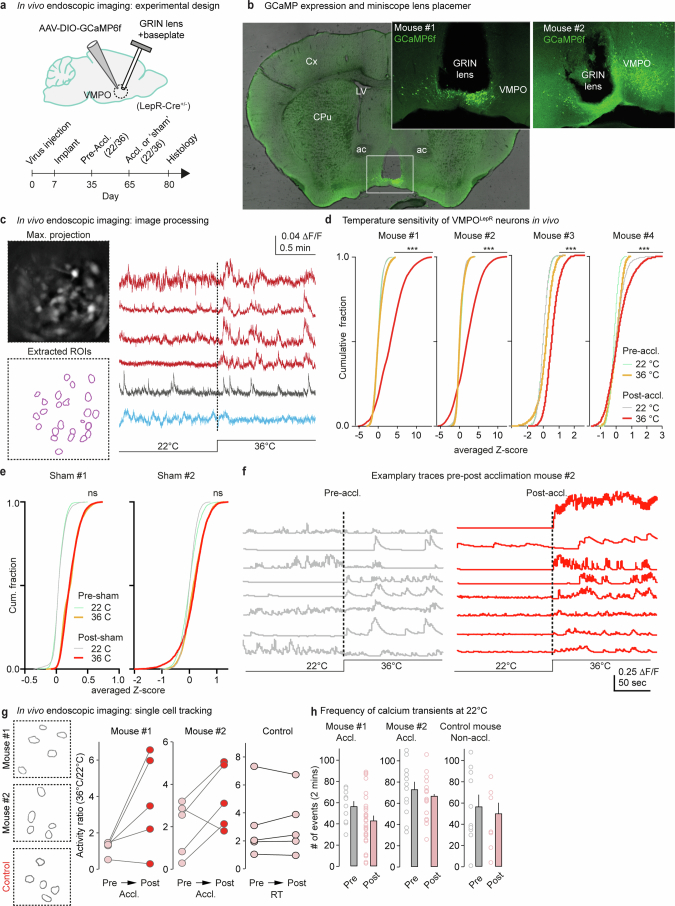
Microendoscopy reveals acclimation-induced VMPO^LepR^ warm responsiveness *in vivo.* **a**, Top: schematic of experimental configuration indicating AAV-mediated GCaMP6f delivery into VMPO^LepR^ neurons and GRIN lens implantation. Bottom: experimental timeline. **b**, Representative images showing GCaMP6f expression in VMPO^LepR^ neurons, and location of the GRIN lens implant. **c**, Left: maximal projection image from a representative imaging session and location of the regions of interest (ROI). Right: calcium dynamics from 6 representative neurons recorded from a non-acclimated mouse at RT (22 °C) and at 36 °C. Note that some cells increase (red traces), decrease (blue trace) and don’t change (grey trace) activity. **d**, Cumulative distribution plots of the activity (averaged z-scores) of all extracted neurons in 4 mice upon increasing ambient temperature acutely from RT (22 °C) to 36 °C, before and after acclimation for 30 days at 36 °C. Mann-Whitney U test, ***P < 0.0001 for cumulative fraction at 36 °C Pre vs Post acclimation. **e**, Cumulative distribution plots of the activity (averaged z-scores) of all extracted neurons in 2 time-matched control mice upon increasing ambient temperature acutely from RT to 36 °C, before and after sham acclimation. The time-matched controls did not undergo acclimation to temperature but stayed at at 22 °C for 30 days in between recording sessions. Mann-Whitney U test, P = 0.2273 (Sham #1) and P = 0.3624 (Sham #2) for cumulative fraction at 36 °C Pre vs Post sham acclimation. **f**, Representative calcium dynamics (ΔF/F) traces in 8 randomly selected cells from mouse #2 from panel **d** before (Pre-accl.) and after heat acclimation (Post-accl.). **g**, Representative VMPO^LepR^ that could be reliably tracked and recorded before and after acclimation (30 days in between recording sessions) in 2 acclimated mice, and in a time-matched RT control mouse. Activity of single cells upon changes in ambient temperature from 22 °C to 36 °C. Cells were longitudinally recorded before (pre) and after (post) temperature acclimation. **h**, Summary plots of calcium transients in 2 mice (left, middle) before and after heat acclimation with the mouse kept at 22 °C during recordings and a non-acclimated mouse with matched inter-recording interval in between recording sessions (30 days) (right); related to panel h. Data based on N=4 acclimated animals and N=2 time-matched non-acclimated animals. Data shown as mean ± s.e.m.

Next, we assessed whether heat acclimation also induced activity changes of VMPO^LepR^ neurons in vivo. To this end, we stereotactically delivered the Cre-dependent calcium sensor GCaMP6f into the VMPO of LepR-Cre mice and performed micro-endoscopic (Miniscope) imaging in freely moving mice^29^ before and after heat acclimation (Extended Data Fig. 3a,b). Indeed, acclimation increased the heat responsiveness of VMPO^LepR^ neurons in vivo. Not only did we find that more VMPO^LepR^ neurons responded to a heat challenge subsequent to acclimation, but that the neurons also responded more robustly (Fig. 1g,h, Extended Data Fig. 3c–g and Supplementary Videos 1 and 2). This observation agrees with findings showing that increases in body temperature (on a heat challenge) are directly transferred to POA neurons^27,30^.

Although it is technically challenging to register and follow individual neurons by Miniscope imaging over the extended acclimation period, such an analysis did not reveal an increase in acclimation-induced baseline activity at 22 °C ambient temperature (Extended Data Fig. 3h).

### Enhanced VMPO^LepR^ neuron activity mediates heat tolerance

**Fig. 2:**
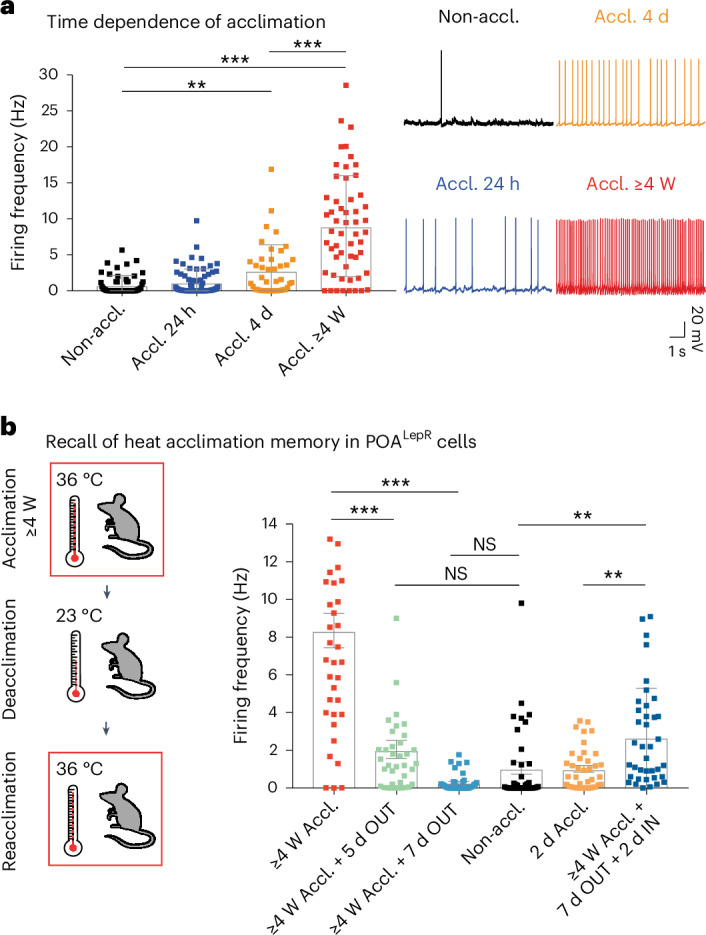
Kinetics of VMPO^LepR^ neuron acclimation, deacclimation and reacclimation. **a**, Left: AP firing frequencies of VMPO^LepR^ neurons recorded from non-acclimated mice and mice acclimated for 24 h, 4 d and 4 weeks (full acclimation). Kruskal–Wallis test (*H* = 69.51, degrees of freedom (d.f.) = 3, *P* < 0.0001) with Dunn’s pairwise comparisons and Bonferroni’s corrections: ^**^P = 0.0062 (Non-accl.:Accl. 4 d), ^***^P < 0.0001 (Non-accl.:Accl. ≥4 weeks), ^***^P = 0.0005 (Accl. 4 d:Accl. ≥4 weeks; *n* = 42/5 per group). Right: representative traces of AP firing patterns as a function of heat acclimation duration, recorded in VMPO^LepR^ neurons. Brain slices were recorded at 33 °C bath temperature (mean ± s.d.). **b**, AP firing frequency (Hz) measured in VMPO^LepR^ neurons from LepR-Cre;HTB mice after different acclimation, deacclimation and reacclimation periods. Non-accl. control (black), 2-d Accl. (orange), full acclimation (≥4 weeks Accl., red), 5 or 7 d of deacclimation after full acclimation (≥4 weeks Accl. + 5 d OUT, green; ≥4 weeks Accl. + 7 d OUT, light blue, respectively) or reacclimation after removing fully (4–5 weeks) acclimated animals for 7 d from the 36 °C acclimation chamber to RT and reacclimating them for only 2 d at 36 °C (≥4 weeks Accl. + 7 d OUT + 2 d IN, dark blue). After full acclimation (4–5 weeks), AP firing returned to baseline after 7 d of deacclimation. Reacclimation for just 2 d significantly elevated AP firing to levels much higher than those achieved by a short 2-d acclimation in naive animals. One-way ANOVA (*F*(5, 189) = 26.85, *P* < 0.001) with Šidák’s multiple-comparison test: ^***^P < 0.0001 (≥4-week Accl.:≥4-week Accl. + 5 d OUT); ^***^P < 0.0001 (≥4-week Accl.:≥4-week Accl. + 7 d OUT); ^**^P = 0.0061 (2-d Accl.:≥4-week Accl. + 5 d OUT + 2 d IN); ^**^P = 0.0061 (Non-accl.:≥4-week Accl. + 5 d OUT + 2 d IN); ^**^P = 0.004 (2-d Accl.:≥4-week Accl. + 7 d OUT + 2 d IN); ^**^P = 0.0002 (Non-accl.:≥4-week Accl. + 7 d OUT + 2 d IN) (*n* = 38/3 cells per group; mean ± s.e.m.). NS, not significant.

Heat acclimation-induced activity increases in VMPO^LepR^ neurons were first detectable ex vivo after 4 d of heat acclimation, further increasing until reaching a maximum at about 4 weeks of acclimation (Fig. 2a); a similar time frame is required to reach a fully heat-acclimated state in rodents, resulting in their increased heat tolerance^31^.

When fully acclimated mice were returned to 23 °C ambient temperature, AP firing in VMPO^LepR^ neurons subsided to baseline levels within 7 d (Fig. 2b). However, an ‘adaptive memory’ remained: subsequent to a 7-d deacclimation phase at 23 °C, high AP firing rates in VMPO^LepR^ neurons were quickly retrieved when animals were placed again at 36 °C for only 2 d, reaching significantly higher AP firing rates compared with naive mice subjected to a 2-d acclimation period for the first time (Fig. 2b). This property is reminiscent of acclimation-induced adaptations observed in peripheral organs promoting heat tolerance, which are also quickly recalled after primed acclimation^31,32^. We therefore wondered whether heat acclimation-induced tonic activity in VMPO^LepR^ neurons mediates this adaptive response and conveys heat tolerance.

**Fig. 3:**
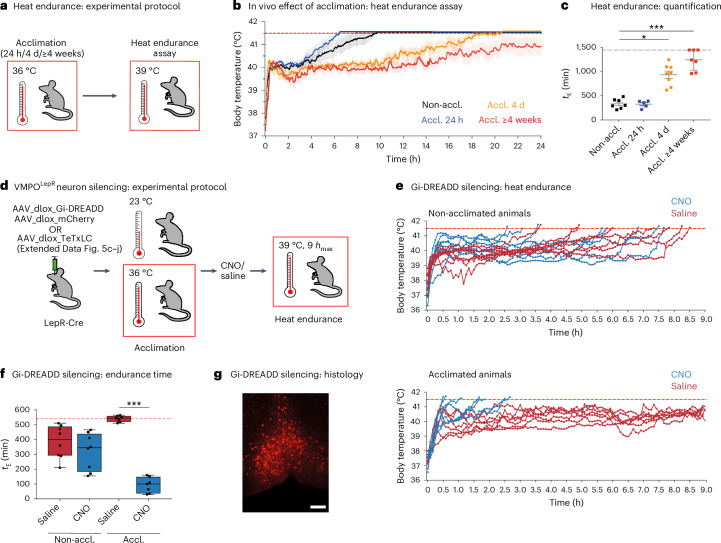
Increased heat tolerance after heat acclimation is dependent on VMPO^LepR^ neuron activity. **a**, Heat endurance assay. **b**, Average body temperature (mean ± s.e.m.) of non-acclimated (black; *N* = 7), 24-h (blue; *N* = 5), 4-d (orange; *N* = 8) and 4- to 5-week (red; *N* = 7) acclimated animals in the heat endurance assay monitored for a maximum of 24 h or until the animal reached the cut-off temperature of 41.5 °C (dashed red line). **c**, Endurance time (*t*_E_; minutes) of mice shown on the left. The cut-off time is 24 h (dashed gray line). Kruskal–Wallis test: *H* = 20.78, d.f. = 3, *P* < 0.0001, with Dunn’s pairwise comparisons and Bonferroni’s corrections: ^*^P = 0.0262 (Non-accl:Accl. 4 d), ^***^P = 0.0006 (Non-accl.:Accl. ≥4 weeks). The error bars represent the mean ± s.e.m. **d**, Schematic showing the two experimental strategies used to interfere with VMPO^LepR^ neuron activity. *h*_max_, assay cut-off time. **e**, Heat endurance assay of Gi-DREADD-expressing mice. Non-acclimated (top) or acclimated (bottom) animals were injected with either CNO (i.p. 0.3 mg kg^−1^) or saline 10 min before the assay and the body temperature was continuously monitored. Non-acclimated animals endured for similarly short times, independent of whether they received CNO or vehicle (saline). In acclimated mice, CNO injection (but not saline injection) eliminated acquired heat tolerance and the animals quickly reached the cut-off temperature (41.5 °C). **f**, The *t*_E_ for the groups shown in **e**. Box plots show the median and IQR. Kruskal–Wallis test: *H* = 24.33, d.f. = 3, *P* < 0.0001, with Dunn’s pairwise comparisons and Bonferroni’s corrections: ^***^P < 0.0001 (Accl. saline:CNO); *N* = 8 animals for Non-accl. groups and *N* = 7 for Accl. groups. Note that, as a result of the assay cut-off time of 9 h, the heat tolerance capacity (*t*_E_) of the acclimated saline-treated group is underestimated. **g**, Representative image of VMPO^LepR^ neurons showing mCherry labeling of the Gi-DREADD-mCherry fusion protein. Scale bar, 250 μm. Box plots show the median and IQR (Extended Data Figs. 4 and 5).

**Extended Data Fig. 4:**
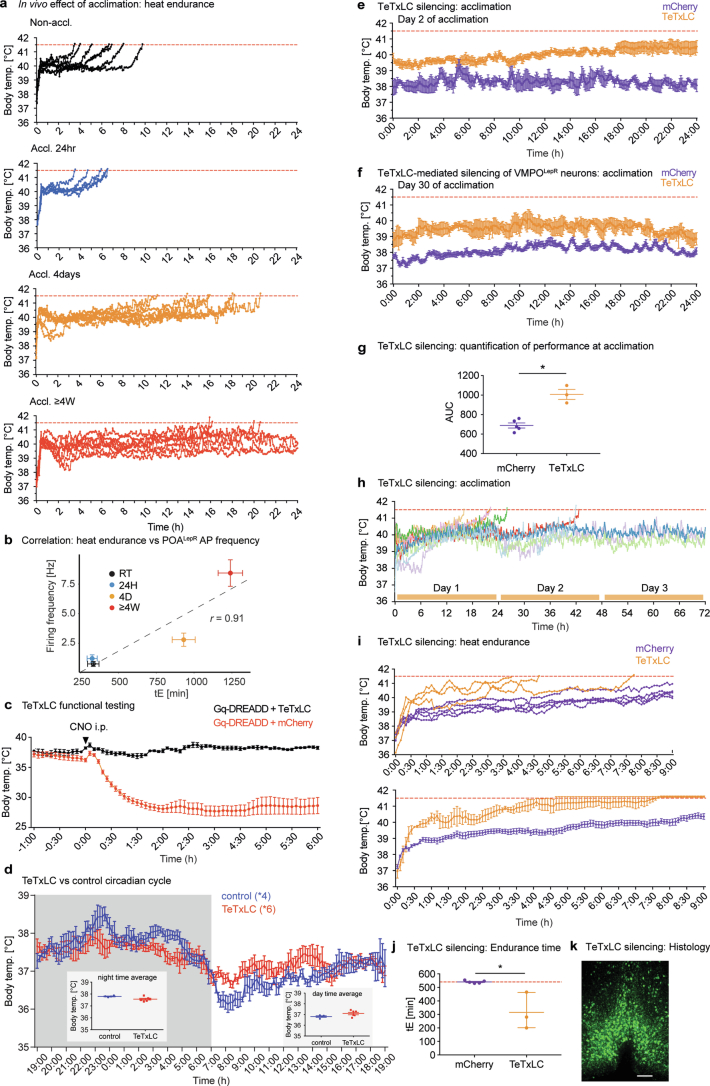
Heat acclimation-induced heat tolerance is blocked by TeTxLC-mediated VMPO^LepR^ silencing. **a**, Body temperature of individual non-acclimated (Non-Accl., black, N = 7), 24hr-acclimated (Accl. 24hr, blue, N = 5), 4 days-acclimated (Accl. 4days, orange, N = 8), and ≥ 4 weeks acclimated (Accl. ≥ 4W, red, N = 7) mice during 24-hour heat endurance assay. **b**, Correlation plot between heat endurance time (tE) and average firing frequency of VMPO^LepR^ neurons after varying duration of acclimation. Pearson's (r) correlation coefficient between the two parameters is shown. **c**, TeTxLC functionality was tested by measuring body temperature after CNO injection in mice with VMPO^LepR^ neurons expressing Gq-DREADD or Gq-DREADD+TeTxLC (N = 4 per group). Only mice without TeTxLC showed a temperature decrease. **d**, Average body temperature over 24 hours at RT shows no difference between TeTxLC- (N = 6) and mCherry-infected (N = 4) animals. Two-way ANOVA (effect of treatment * time: F (144, 1152) = 2.236, P < 0.0001) with Sidak’s multiple comparisons (P = ns). Insets: Mean body temperature of the two groups during nighttime (left) and daytime (right). **e, f**, Average body temperature of TeTxLC- and mCherry-infected animals at day 2 (**e**) and day 30 (**f**) of heat acclimation, showing that TeTxLC-animals are hyperthermic. Note that during heat acclimation 6 of the 9 TeTxLC-animals dropped out. At day 2: N = 6 and N = 5 for TeTxLC and mCherry, respectively. At day 30: N = 3 and N= 5 for TeTxLC and mCherry, respectively. **g**, Quantification of the area under the curve (AUC) calculated for the two groups for the last day of acclimation. Mann-Whitney U test (two-tailed), *P = 0.0286. N = 3 mice for TeTxLC and N = 5 mice for mCherry control. **h**, Body temperature traces of individual TeTxLC-silenced LepR-Cre animals are shown for the first 3 days of heat acclimation (36 °C). 6 out of 9 animals with silenced VMPO^LepR^ neuron outputs reached the Tcore cut-off. **i**, TeTxLC-silenced animals that completed the 30-day acclimation cycle (N = 3) were tested side-by-side with control animals (N = 5) in the heat endurance assay. Top: Body temperature traces of individual mice. Bottom: average body temperature traces for the TeTxLC- and mCherry-expressing groups. **j**, Quantification of the endurance time (tE) in the 9-hour (540 min) heat endurance assay for the two groups. Mann-Whitney U test (two-tailed); *P = 0.0262 (mCherry : TeTxLC). N = 3 for TeTxLC and N = 5 for mCherry control mice. Data presented as mean ± SD. **k**, Representative image of VMPO^LepR^ neurons labelled with EGFP that is co-expressed with TeTxLC; Size bar = 250 μm. All data represent as mean ± s.e.m with the exception of panel (**j**).

Heat tolerance expands the limit of tolerable temperatures^33–35^. To assess the beneficial autonomic effects of acclimatization in vivo and probe the tolerance to heat, we utilized a heat endurance assay during which the animal is challenged with hot ambient temperatures (39 °C) while the body temperature (*T*_core_) is monitored telemetrically (Fig. 3a,b)^36^. Non-acclimated mice were able to keep their *T*_core_ < 41.5 °C—demarcating the maximal *T*_core_ that mice are able to tolerate^33,37^—for an average endurance time (*t*_E_) of only 333.6 ± 37.6 min (mean ± s.e.m.). In opposition to this, animals acclimated at 36 °C for ≥4 weeks were able to sustain their *T*_core_ within the physiological range for long time periods (*t*_E_ = 1,235 ± 81.3), with some animals even exceeding a full circadian cycle (Fig. 3b,c and Extended Data Fig. 4a), attesting to the high heat tolerance level that they had gained after acclimation. We found that longer acclimation periods enhanced heat tolerance more robustly than shorter acclimation periods and, interestingly, increased heat endurance correlated with increased average AP firing frequencies of VMPO^LepR^ neurons (Extended Data Fig. 4b).

To address whether acclimation-induced activity in—and resulting synaptic output of—VMPO^LepR^ neurons is required for gaining heat tolerance, we silenced the cells by virally delivering Cre-dependent tetanus toxin light chain (TeTxLC)^38^ into the POA of LepR-Cre mice before acclimation. We verified the effectiveness of TeTxLC silencing (Extended Data Fig. 4c). Although the *T*_core_ and overall behavior of TeTxLC-silenced animals were normal at ambient temperatures of 23 °C (Extended Data Fig. 4d), the mice were compromised during the 36 °C acclimation phase and presented with higher *T*_core_ temperatures than littermate controls (Extended Data Fig. 4e–g); several animals reached 41.5 °C during the first 2 d of acclimation (Extended Data Fig. 4h) and thus could not be tested in the heat endurance assay. Presumably, strong and permanent TeTxLC-mediated inhibition revealed that output of the fraction of rapidly heat-responsive VMPO^LepR^ neurons in non-acclimated mice (Fig. 1g,h and Extended Data Fig. 1a,b) has a role in acute heat defense of the animals.

**Extended Data Fig. 5:**
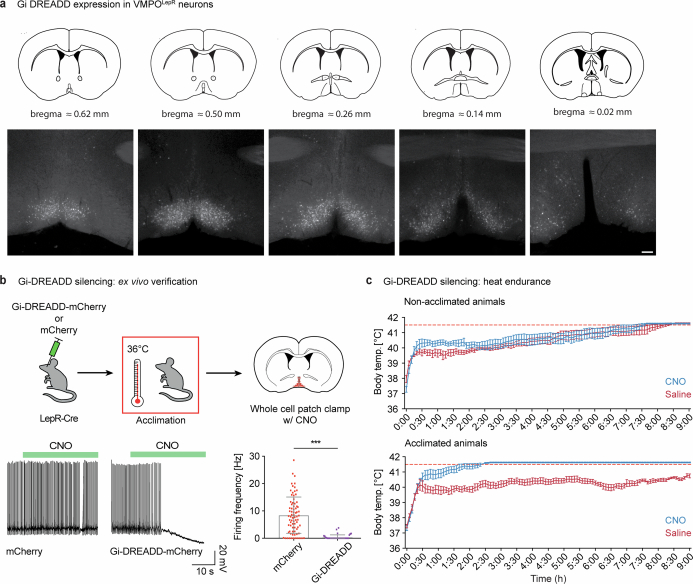
Gi-DREADD-driven inhibition of acclimation-induced VMPO^LepR^ activity prevents heat tolerance. **a**, Schematic drawings representing different anatomical positions along the rostral caudal axis of the preoptic hypothalamic region with the 3 middle drawings (approx. bregma = 0.5 mm to bregma = 014) indicating the center of the VMPO region (top) with corresponding typical fluorescent images depicting the extent of virally (AAV) delivered Cre-dependent Gi-DREADD expression in a LepR-Cre mouse (bottom). **b**, Top: Schematic showing the protocol used for *ex vivo* verification of CNO triggered, Gi-DREADD mediated inhibition of VMPO^LepR^ following heat acclimation. Bottom left: Representative electrophysiological traces showing the effect of CNO on the firing pattern of acclimated VMPO^LepR^ neurons injected with either Cre-dependent Gi-DREADD-mCherry AAV or only a Cre-dependent mCherry control AAV. Bottom right: average (mean ± s.e.m.) tonic AP firing frequency of acclimation-induced VMPO^LepR^ cells in the presence of 5 µM CNO. Mann-Whitney U test (one-tailed), ***P < 0.0001. n = 35/3 cells per group. **c**, Heat endurance assay: Average (mean ± s.e.m.) body temperature of non-acclimated (top) or acclimated (bottom) Gi-DREADD-positive and CNO-injected animals during the heat endurance assay. The same animals injected with saline instead of CNO were also plotted for comparison. N = 8 mice for the non-acclimated and N = 7 mice for the acclimated condition.

The remaining TeTxLC-silenced animals were able to complete the full 30-d acclimation cycle but, subsequently, failed the heat endurance assay and performed similarly to non-acclimated control animals (Extended Data Fig. 4i–k). Although in agreement with VMPO^LepR^ neurons having a role in heat acclimation, this experiment did not provide conclusive evidence that their tonic activity slowly drives the development of heat tolerance over the extended acclimation period. Moreover, this experiment did not allow us to conclude whether heightened acclimation-induced, warm-sensitive activity in VMPO^LepR^ neurons is mediating increased heat tolerance after acclimatization and during the heat endurance assay. To inhibit acclimation-induced AP firing in VMPO^LepR^ neurons subsequent to heat acclimation, we made use of chemogenetic interference using the inhibitory hM4Di (Gi-DREADD) receptor^39^ in LepR-Cre mice (Fig. 3d and Extended Data Fig. 5a). Different from the tetanus toxin approach, virally mediated Gi-DREADD expression in VMPO^LepR^ neurons does not hinder acclimation-relevant adaptive changes to occur, but inhibits neuronal activity only in the presence of the DREADD agonist clozapine *N*-oxide (CNO), which we verified in brain-slice recordings (Extended Data Fig. 5b).

When VMPO^LepR^ neurons were chemogenetically silenced during the heat challenge period, acclimated animals failed to maintain their *T*_core_ within physiological boundaries in the heat endurance assay (Fig. 3e–g and Extended Data Fig. 5c). Strikingly, Gi-DREADD-mediated inhibition resulted in rapid hyperthermia and short endurance times in acclimated animals, whereas it did not accelerate hyperthermia in non-acclimated controls, demonstrating that acclimation-induced, warm-sensitive AP firing of VMPO^LepR^ neurons triggers the utilization of gained heat tolerance capacity.

Collectively, these results suggest that acclimation-induced, VMPO^LepR^ neuron, warm-sensitive activity is necessary for both building up heat tolerance capacity over the course of the acclimation period and recruiting heat tolerance mechanisms on an acute heat challenge.

### LPBN → POA pathway is critical for the induction of heat acclimation

**Extended Data Fig. 6:**
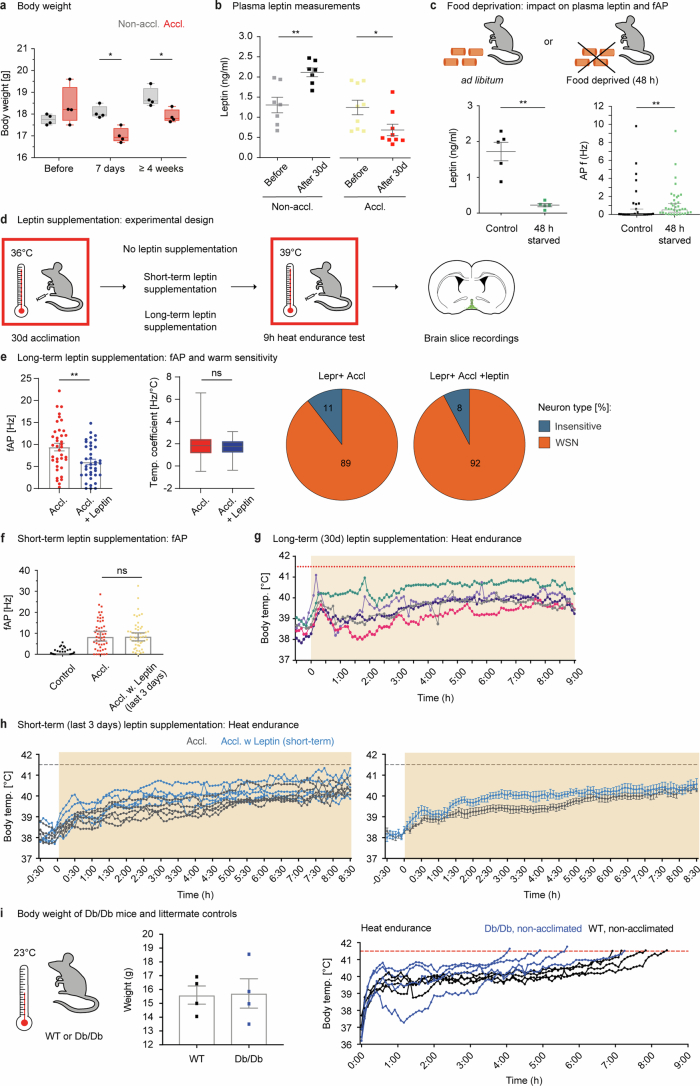
Effect of leptin signaling on VMPO^LepR^ activity and on heat acclimation and heat endurance. **a**, Body weight of acclimated animals decreased and remained significantly lower compared to that of non-acclimated counterparts during the entire acclimation period. Two-way ANOVA (effect of treatment * acclimation time: F (2, 12) = 29.14, P < 0.0001) with Tukey’s pairwise comparisons: *P = 0.0177 (Non-accl. : Accl. at 7 days), *P = 0.0366 (Non-accl. : Accl. at ≥4 weeks). N=4 per group. **b**, Blood plasma leptin measurements in non-acclimated and acclimated animals over the course of 30 days. Mann-Whitney U test (two-tailed), **P = 0.0070 for the non-acclimated condition and *P = 0.0142 for the acclimated condition. N = 7 for Non-accl. and N = 9 for Accl. **c**, Leptin content in the blood and frequency of action potential firing of VMPO^LepR^ neurons were tested upon food-deprivation (48 h). Mann-Whitney U test (two-tailed), **P = 0.0079 for Leptin concentration and **P = 0.0050 for fAP. **d**, LepR-Cre;HTB mice received i.p. leptin injections twice daily for the last 3 days (short-term) or the entire 30-day acclimation (long-term). Controls received saline. Post-acclimation, mice underwent a 9-hour heat endurance test, followed by *ex vivo* recording of VMPOLepR neuron activity. **e**, Left: long-term leptin treatment slightly reduced fAP in acclimated VMPO^LepR^. Unpaired two-tailed t-test, **P = 0.0035. n = 38/5 (Accl.) and n = 39/3 (Accl. + Leptin) cells. Middle: long-term supplementation of leptin did not have an effect on warm-sensitivity of VMPO^LepR^ neurons, measured by temperature coefficient. n = 38/5 (Accl.) and n = 39/3 (Accl. + Leptin) cells. Right: Distribution of temperature-insensitive, and warm-sensitive (WSN) within acclimated VMPO^LepR^ control group (n = 38/5 cells) and VMPO^LepR^ group (39/3 cells). Recordings were performed at at 36 °C using ‘high-K^+^ aCSF’ and in the presence of synaptic blockers CNQX, AP-V and gabazine. **f**, Short-term leptin treatment did not have any impact on fAP in acclimated VMPO^LepR^; non-acclimated VMPO^LepR^ group was plotted for visual comparison. n = 51/5 (Accl.) and n = 51/5 (Accl. + Leptin 100 nM in aCSF) cells. Tonic neuronal activity was recoded without synaptic blockade, using ‘low-K+ aCSF’ and at 33 °C bath temperature. **g**, Body temperature traces of LepR-Cre;HTB animals during the heat endurance assay following long-term supplementation of leptin during 30d of heat acclimation at 36 °C. N = 5 mice. **h**, Body temperature traces of individual (left) LepR-Cre;HTB animals following short-term supplementation of leptin duringheat challenge. Group averages are presented in the right panel. N = 5 animals each. **i**, Db/db animals were pair-fed with littermate control mice for 1 week and kept at room temperature (22 °C/23 °C) before undergoing the 9-hour heat endurance assay. Middle: body weight was comparable between the two groups prior to the assay. Right: Both control and Db/Db mice reached the body temperature cut-off of 41.5 °C prior to the conclusion of the 9-hour period. N = 4 animals per group. All data presented as mean ± s.e.m. Boxplots represent median and interquartile range.

In line with a reduction in body weight (Extended Data Fig. 6a), a decline in blood plasma leptin levels paralleled the increase in warm-sensitive firing when mice were heat acclimated (Extended Data Fig. 6b). Leptin signaling has been implicated in POA-orchestrated thermoregulation and body temperature adaptation^40–42^. We therefore wondered whether a reduction in leptin levels during acclimation is a prerequisite for—or permissive of—the induction of AP firing increases in VMPO^LepR^ neurons. We found that modulating leptin levels in vivo, either by food deprivation (which naturally lowers leptin levels) or by supplementing leptin by intraperitoneal (i.p.) injections during acclimation, had only a small or negligible effect on VMPO^LepR^ neuron activity or the performance of acclimated animals in the heat endurance assay, respectively (Extended Data Fig. 6c–h).

To assess whether the absence of leptin signaling may promote heat tolerance, we also tested whether leptin receptor-deficient Db/Db mice^43^ would be better equipped to cope with 39 °C heat without prior heat acclimation. However, we found that Db/Db mice did not perform longer in the heat endurance assay compared with their pair-fed and weight-matched littermate controls (Extended Data Fig. 6i). We thus concluded that the reduction in leptin levels has a minor role in shaping VMPO^LepR^ neuron activity and heat acclimation.

**Extended Data Fig. 7:**
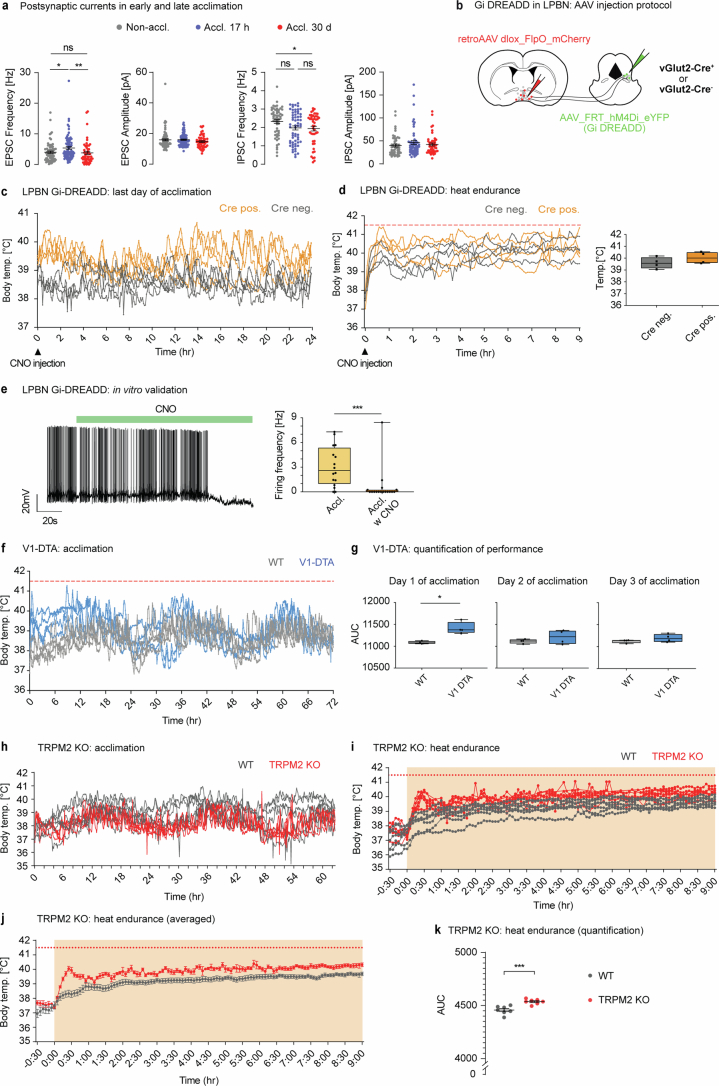
Thermo-afferent pathways via the PBN are required during the initial phase of heat acclimation but appear to become obsolete at late acclimation stages. **a**, Spontaneous EPSC and IPSC in VMPOLepR neurons from non-acclimated, short-term (17 h), and long-term (30 days) acclimated LepR-Cre;HTB mice. Left: EPSC frequency increased after 17 hours of acclimation and returned to baseline after 30 days. Kruskal-Wallis test, P = 0.0017; Dunn’s multiple comparisons test, *P = 0.0328 (Non-accl. : Accl. 17h), **P = 0.0024 (Accl. 17h : Accl. 30d). n = 74/4 (Non-accl.), n = 82/4 (Accl. 17h) and n = 51/4 (Accl. 30d) cells. 2^nd^ from left: EPSC amplitude did not change between the conditions tested. 3^rd^ from left: IPSC frequency was found to decrease over the course of acclimation. One-way ANOVA, P = 0.0345; Tukey’s multiple comparison test, *P = 0.0497 (Non-ccl.:Accl. 30d). n = 57/3 (Non-accl.), n = 60/3 (Accl. 17h) and n = 46/3 (Accl. 30d) cells. 4^th^ from left: IPSC amplitude did not change between the conditions tested. Data shown as mean ± s.e.m. **b**, Strategy for Gi-DREADD delivery into glutamatergic LPBN neurons that target VMPO using the Vglut2-Cre mouse line. **c**, Gi-DREADD mediated inhibition of PBN→POA projection neurons renders Cre-positive animals (orange), but not Cre-negative controls (grey), slightly hyperthermic after CNO injection at the end of acclimation. **d**, CNO-mediated inhibition of these PBN→POA projection neurons during the heat endurance assay did not perturb heat tolerance. Left: Body temperature traces of individual Cre-positive (orange, N = 4) and Cre-negative (grey, N = 4) mice during heat endurance assay. Right: Body temperature of mice expressing Gi-DREADD and AAV-injected controls during the assay. **e**, Electrophysiological traces showing the effect of CNO on the firing pattern of Gi-DREADD-expressing PBN neurons recorded from an acclimated mouse *ex vivo*. Right panel: average AP firing frequency (n=17) of control and CNO treated cells. Mann-Whitney U test (two-side); ***P < 0.0001. n = 17/2 cells each. **f**, Body temperature of individual V1-DTA ablated and wildtype littermate control animals (N = 4 per group) during the first three days of heat acclimation. **g**, Area under the curve (AUC) calculated from body temperature recordings for three consecutive days of acclimation (Day 1 = 0–24 h, Day 2 = 24–48 h and Day 3 = 48–72 h) for the V1-DTA ablated and control mouse groups. Mann-Whitney U test (two-tailed); *P = 0.0286. N = 4 animals per group. **h**, Body temperature traces of individual TRPM2-KO and control animals during the first 3 days of acclimation. N = 7 mice each. **i**, **j**, Individual (i) and average (± s.e.m.) (j) body temperature traces TRPM2-KO and control animals during the heat endurance assay. N = 7 mice per group. **k**, Quantification of the area under curve (AUC) for TRPM2-KO and control animals during the heat endurance assay. Unpaired two-tailed t-test, ***P = 0.0006. N = 7 mice per group. Boxplots represent median and interquartile range.

Given the results, we hypothesized that synaptic transmission could serve as an initial trigger of the observed neuronal plasticity mechanism. Intriguingly, at the early stages (~17 h after placing animals at 36 °C) but not at the late stages of heat acclimation, we found that VMPO^LepR^ neurons receive a higher frequency of excitatory synaptic inputs compared with non-acclimated animals (Extended Data Fig. 7a).

**Fig. 4:**
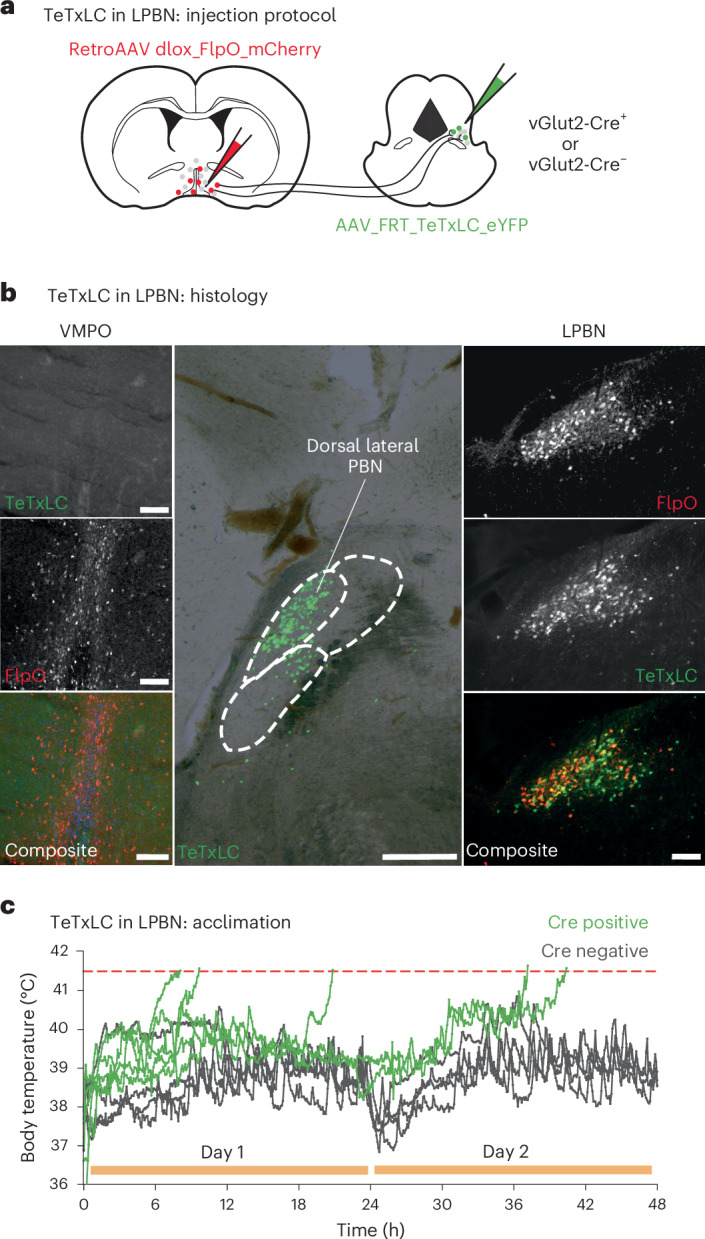
Thermoafferent LPBN pathway is required to trigger heat acclimation. **a**, Schematic showing the viral injection strategy for TeTxLC-mediated silencing of excitatory (Vglut2-positive) presynaptic neurons located in the LPBN and innervating VMPO. **b**, Example images showing the expression of AAV-FRT-TeTxLC-EGFP (green) and retroAAV-dlox-FlpO-mCherry (red) in the VMPO (left) and in VMPO-projecting LPBN neurons (right). Scale bars, 250 μm. The histological labeling confirmed double infection of glutamatergic LPBN neurons in Vglut2-Cre mice expressing the recombinase FlpO (red; derived from the retroAAV injected into VMPO) and TeTxLC (green; derived from Cre- and FlpO-dependent AAV particles injected into the LPBN) (middle). Scale bar, 100 μm. Note that labeled neurons are mainly located in the dorsal lateral part of the LPBN; no TeTxLC is detectable in the POA (top left), assuring that inhibition happened at the level of the LPBN but not the POA. **c**, Body temperature traces of individual LPBN → VMPO silenced (Cre-positive, green, *N* = 5) and nonsilenced control (Cre-negative, gray, *N* = 5) animals during the initial 48 h of heat acclimation. In contrast to Cre-negative animals, all animals expressing TeTxLC failed to maintain their body temperature <41.5 °C during the first 2 d of acclimation (Extended Data Figs. 6 and 7).

These findings suggested that heat-driven, thermoafferent excitatory synaptic inputs to VMPO^LepR^ neurons could be involved in triggering their plasticity and warm-sensitive tonic firing. Previously, the lateral parabrachial nucleus (LPBN) had been shown to constitute a major hub for thermoafferent pathways that are relayed to the rostral POA^13,14,17^. We therefore wondered whether synaptic LPBN → VMPO transmission is important for acclimation. Thermoregulatory LPBN neurons innervating the POA are Vglut2 positive^17^. This allowed us to use Vglut2-Cre mice in combination with a dual viral delivery strategy to selectively silence those LPBN projections reaching the VMPO: first we stereotactically supplied Cre-dependent FlpO retroAAV (adeno-associated virus) particles (designed to infect axonal nerve terminals^44^) into the POA. Subsequently, we injected AAV particles expressing FlpO-dependent TeTxLC into the LPBN (Fig. 4a,b). Although silencing LPBN → POA transmission did not alter the baseline *T*_core_ of mice kept at normal (23 °C) ambient temperatures, it prevented acclimation and mice were unable to maintain their *T*_core_ within the physiological range when placed at 36 °C (Fig. 4c), a result similar to that observed when silencing VMPO^LepR^ neurons directly (Extended Data Fig. 4h).

However, unlike transiently blocking VMPO^LepR^ neurons at the end of the acclimation period, transiently blocking VMPO-projecting LPBN neurons after a 30-d heat acclimation period did not abrogate heat tolerance: we again used a dual viral delivery strategy (Extended Data Fig. 7b) but, in this case, to temporarily silence LPBN → POA projection neurons via Gi-DREADD. Injecting CNO into Vglut2-Cre mice at the end of their long-term acclimation phase slightly (and fairly briefly) increased the *T*_core_ (Extended Data Fig. 7c), but did not interfere with their performance in the heat endurance assay (Extended Data Fig. 7d). Subsequently, we verified the effectiveness of Gi-DREADD receptors in silencing LPBN → POA projection neurons ex vivo (Extended Data Fig. 7e).

These results are consistent with a role for LPBN → VMPO projections in the initial induction of heat acclimation, but this pathway plays only a minor role, if any, in driving and sustaining long-term heat acclimation.

Congruent with this hypothesis, we found that Trpv1-Cre;DTA mice, lacking most—but not all—peripheral thermosensory neurons as a result of the genetically controlled expression of the diphtheria toxin^45,46^ were slightly, but significantly, hyperthermic at the beginning of the acclimation phase (Extended Data Fig. 7f,g), in agreement with previous acute heat-challenge results^45^. However, body temperatures recovered to normal levels by days 2–3 of acclimation, indicating that, with a delay, another (peripheral or central) mechanism was able to compensate for reduced primary afferent thermosensory signals. Similarly, the TRPM2 ion channel, which previously has been implicated in the acute detection of warm or hot temperatures in the peripheral and central nervous systems^27,47,48^, appeared largely dispensable for long-term heat acclimation, and both groups (TRPM2 knock-out (KO) and control mice) performed similarly in the heat endurance assay (Extended Data Fig. 7h–k).

Together, these results suggest that thermoafferent excitatory synaptic pathways via the LPBN are largely important at the beginning of heat acclimation, presumably to trigger adaptive plasticity in VMPO^LepR^ neurons, which thereby become autonomous, warm-sensitive, pacemaker neurons.

### Long-lasting VMPO^LepR^ neuron activity increases heat tolerance

**Extended Data Fig. 8:**
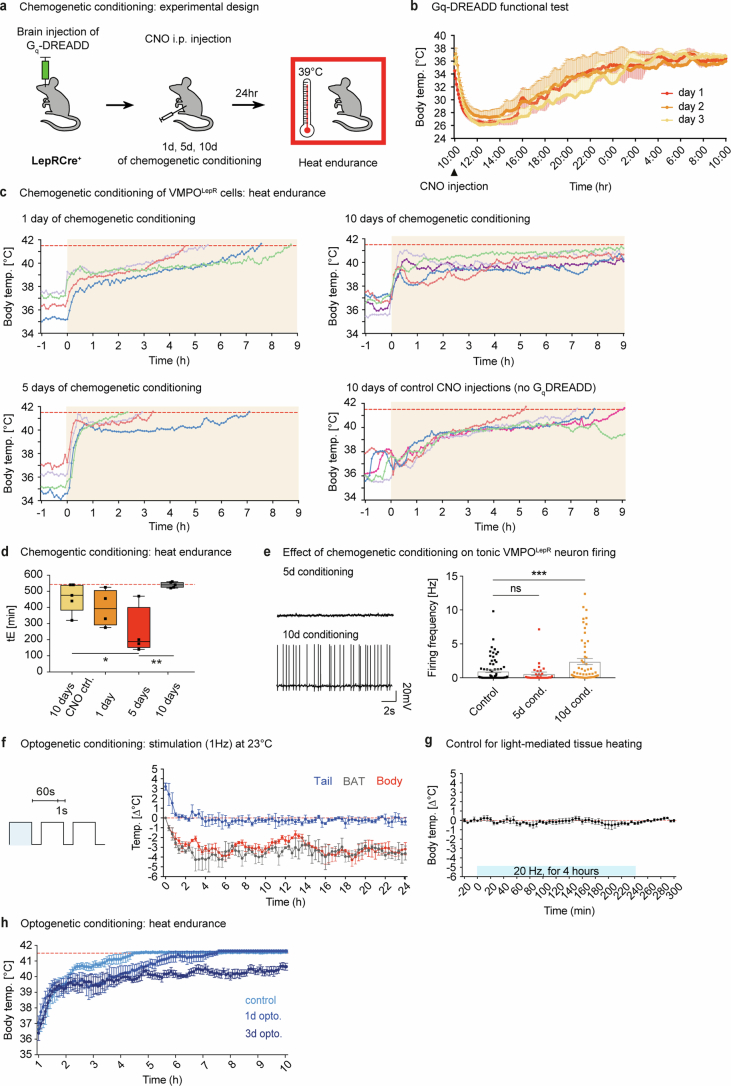
Long-term activation of VMPO^LepR^ by chemogenetic (Gq-DREADD) or optogenetic (ChR2) conditioning is sufficient to induce heat tolerance in the heat endurance assay. **a**, Viral injection of Cre-dependent Gq-DREADD into the rostral POA of LepR-Cre mice. VMPO^LepR^ neurons were chemogenetically activated (conditioned) by daily injection of CNO (0.3 mg/kg i.p.) for 1, 5 or 10 consecutive days. CNO injections were terminated 24 hours prior to heat endurance assay. **b**, Chemogenetic conditioning of VMPO^LepR^ cells via Gq-DREADD animals produced significant hypothermia that is protracted for up to 10 hours after CNO injection and could be repeated over multiple consecutive days. Traces represent group average (mean ± s.e.m.) for each day of CNO injection. N = 4 animals. **c**, All animals that were chemogenetically conditioned for 10 days passed the heat endurance assay. Animals that reached the cut-off temperature, demarcated by the dashed red line, were discontinued from the assay. **d**, Boxplots (median and IQR) showing endurance times (tE) before reaching 41.5 °C, corresponding to (c). The maximum tE was 9 hours (540 min, red dashed line). All chemogenetically conditioned animals for 10 days reached this maximum. Kruskal-Wallis test (H = 12.67, d.f = 3, P = 0.0006) with Sidak’s multiple comparison test, *P = 0.0312 **P = 0.0034. N = 5 for Control (animals that received saline injections for 10 days), N = 4 for ‘1 day’ (a single CNO injection), N = 4 for ‘5 day’ (5 days of CNO injections) and N = 5 for ‘10 day’ (10 days of CNO injections). **e**, Left: Representative traces of AP firing patterns of two VMPO^LepR^ neurons recorded 24 hours after chemogenetic conditioning for 5 or 10 days. Right: average AP firing frequency (mean ± s.e.m.) of VMPO^LepR^ neurons from non-stimulated control LepR-Cre;HTB animals and from animals chemogenetically conditioned for 5 or 10 days. Kruskal-Wallis test (H = 17.56, d.f = 2, P < 0.0001) with Dunn’s pairwise comparisons and Bonferroni corrections, ***P < 0.0001 (Control: 10d cond.). n = 42/4 cells per group. Neuronal recordings were performed without synaptic blockade, using ‘low-K^+^ aCSF’ and at 33 °C. **f**, Average body-, brown adipose- (BAT-) and tail temperature of LepR-Cre mice (N = 3) expressing Cre-dependent ChR2 and stimulated with blue light at a low frequency of 1 Hz at room ambient temperature (23 °C). **g**, Optogenetic control experiment: in the absence of ChR2, light stimulation of the POA/VMPO of up to 20 Hz continuously for 4 hours did not affect body temperature in freely moving mice (N = 4). **h**, Average body temperature (mean ± s.e.m.) during heat endurance of LepR-Cre mice expressing ChR2 in VMPO^LepR^ neurons that were either not optogenetically conditioned (control, light blue trace), conditioned for 1 day (1d opto, blue trace) or 3 days (3d opto, dark blue trace). All mice were optically stimulated with light pulses at 1 Hz during the heat endurance assay. N = 4 animals each.

We wondered whether we could mimic this process by continued, long-term activation of VMPO^LepR^ neurons in the absence of a warming stimulus. To this end, we implemented a chemogenetic gain-of-function approach. Stereotactic viral delivery and Cre-dependent expression of the chemogenetic activator hM3Dq (Gq-DREADD)^39^ allowed us to stimulate the neurons repetitively by injecting CNO every 24 h for 1, 5 and 10 d (Extended Data Fig. 8a,b). We found that ‘chemogenetic conditioning’ of the animals by increasing the activity of VMPO^LepR^ neurons for 10 d (but not for ≤5 d) before the heat endurance assay was sufficient to induce increased heat tolerance and, somewhat surprisingly, also slightly increased tonic activity in VMPO^LepR^ neurons assessed in brain-slice recordings (Extended Data Fig. 8c–e).

**Fig. 5:**
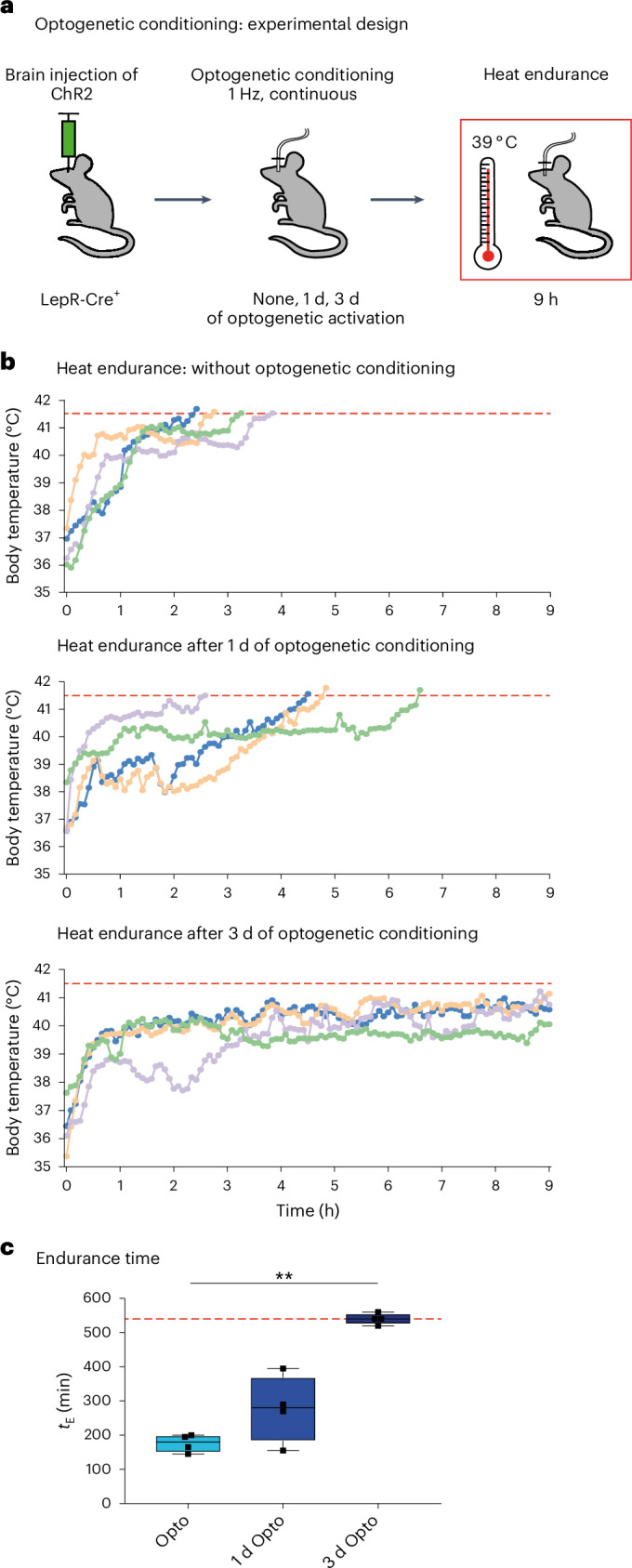
Optogenetic conditioning of VMPO^LepR^ neurons induces heat tolerance. **a**, Experimental paradigm used for continuous optogenetic activation of VMPO^LepR^ neurons before the heat endurance assay. LepR-Cre animals were injected with Cre-dependent ChR2 AAV particles into the rostral POA and either not stimulated or stimulated for 1 or 3 d by blue light at a low frequency (1 Hz) before the heat endurance assay. All animals were optogenetically stimulated during the heat endurance assay. **b**, Body temperature of individual mice subjected to optogenetic conditioning. Only those animals conditioned for 3 d had acquired heat tolerance and performed robustly in the heat endurance assay. Animals that reached the cut-off temperature of 41.5 °C were removed from the assay; assay duration was limited to 9 h. **c**, Endurance time (*t*_E_) of the differently conditioned groups shown in **b**. Box plots show the median and IQR. Kruskal–Wallis test: *H* = 8.649, d.f. = 2, *P* = 0.0019, with Dunn’s pairwise comparison test and Bonferroni’s corrections: ^**^P = 0.0088 (Opto:3 d Opto) (*N* = 4 per group (Extended Data Fig. 8)).

Gq-DREADD-mediated chemogenetic stimulation of VMPO^LepR^ neurons induces pronounced hypothermia^10^ (Extended Data Fig. 8b), presumably by acutely triggering excessive neuronal activation, thereby potentially also explaining the initial dip in heat tolerance capacity after 5 d (Extended Data Fig. 8d). To have more accurate control over firing rates of VMPO^LepR^ neurons, we next opted for long-term optogenetic stimulation—optogenetic conditioning—by expressing Cre-dependent channelrhodopsin (ChR2) in the POA of LepR-Cre animals (Fig. 5a). We optically stimulated VMPO^LepR^ neurons with a low stimulation frequency of 1 Hz, which still triggered hypothermia, albeit of lower magnitude compared with chemogenetic stimulation (Extended Data Fig. 8f). Optical stimulation of control mice absent ChR2 did not have any measurable effect on the *T*_core_ (Extended Data Fig. 8g), demonstrating that light-induced heating was minimal and did not affect this thermosensitive brain area^27^. Similar to chemogenetic conditioning, continuous optic stimulation for 3 d (but not for shorter periods) also resulted in increased thermotolerance and enhanced performance in the heat endurance assay (Fig. 5b,c and Extended Data Fig. 8h). Collectively, these data demonstrate that long-term increases in VMPO^LepR^ neuron activity can drive the expression of heat tolerance. Hypothermia, induced by artificially activating the neurons at normal ambient temperature (Extended Data Fig. 8b,f), probably influences the acquisition of heat tolerance. As chemogenetic conditioning induces more pronounced hypothermia and requires a longer time to acquire heat tolerance compared with optogenetic conditioning, it is possible that hypothermia slows down the establishment of heat tolerance.

### Ionic basis for acclimation-induced activity of VMPO^LepR^ neurons

To shed light on the molecular underpinnings of the acquired cell-autonomous, warm-sensitive VMPO^LepR^ neuron activity, we analyzed electrophysiological changes that occur in the face of acclimation.

**Fig. 6:**
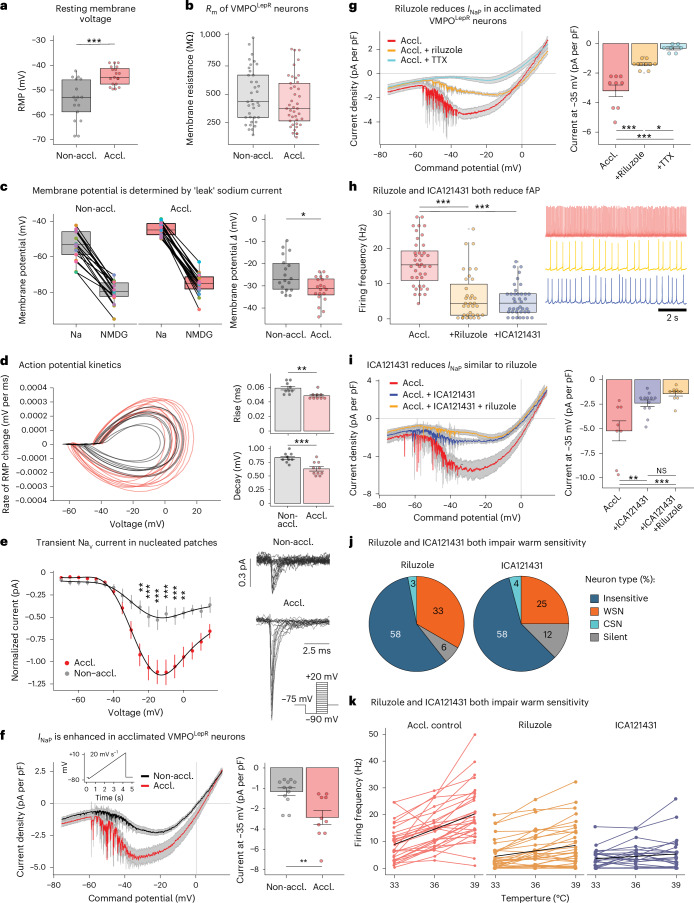
Electrophysiological characterization of VMPO^LepR^ neurons. **a**, RMP in acclimated VMPO^LepR^ neurons (*n* = 19/3) depolarized compared with the RMP of non-acclimated VMPO^LepR^ (*n* = 17/3) cells. Unpaired, two-tailed Student’s *t*-test, ^***^P = 0.0001. **b**, Membrane input resistance (*R*_m_) comparable between non-acclimated (*n* = 37/9) and acclimated (*n* = 41/10) VMPO^LepR^ neurons. **c**, Left: membrane hyperpolarization in non-acclimated (*n* = 17/2) and acclimated (*n* = 19/2) VMPO^LepR^ neurons caused by replacement Na^+^ for NMDG^+^ in aCSF. Right: the difference in membrane potential (*Δ*) between Na^+^-based aCSF and NMDG^+^-based aCSF is larger in acclimated VMPO^LepR^ neurons. Unpaired, two-tailed Student’s *t*-test, ^*^P = 0.0201. **d**, Left: AP phase plot of non-acclimated (gray, *n* = 9/4) and acclimated (red, *n* = 10/5) VMPO^LepR^ neurons. Right: both AP 10–90% rise time (Wilcoxon’s test, ^**^P = 0.0046) and 90% to 10% decay time (unpaired, two-tailed Student’s *t*-test, ^***^P = 0.0006) are significantly faster in VMPO^LepR^ neurons after acclimation. **e**, Left: current–voltage relationship for VMPO^LepR^ neuron peak transient Na_V_ currents recorded in nucleated patches. Two-way ANOVA (effect of acclimation voltage, ^*^P < 0.0001; Tukey’s multiple-comparison test, ^**^P = 0.0016 (−25 mV), ^***^P = 0.0003 (−20 mV), ^***^P = 0.0002 (−15 mV), ^***^P < 0.0001 (−10 mV), ^***^P = 0.0005 (−5 mV) and ^**^P = 0.0072 (0 mV); *n* = 6/2 (Non-accl.) and *n* = 6/2 (Accl.) cells). Right: example of transient Na_V_ current recordings from VMPO^LepR^ neurons. Inset: voltage step protocol used. **f**, Left: average *I*_NaP_, revealed by slow depolarizing voltage ramp, enhanced after heat acclimation (*n* = 12/4 (Non-accl.) and *n* = 10/4 (Accl.) cells). Inset: ramp protocol used to record *I*_NaP_. Right: quantification of *I*_NaP_ at −35 mV based on data shown on the left. Unpaired, two-tailed Student’s *t*-test, ^*^P = 0.0055. **g**, Left: *I*_NaP_ in acclimated VMPO^LepR^ neurons reduced by riluzole (10 µM) and completely blocked by TTX (1 µM). Right: quantification of *I*_NaP_ at −35 mV based on data shown on the left. One-way ANOVA, *P* < 0.0001; Tukey’s multiple-comparison test, ^***^P < 0.0001 (Accl.:Accl. + riluzole), ^***^P < 0.0001 (Accl.:Accl. + TTX), ^*^P = 0.0170 (Accl. + riluzole:Accl. + TTX) (*n* = 9/2 (Accl.), *n* = 10/2 (riluzole) and *n* = 7/2 (TTX) cells). **h**, Left: firing frequency (fAP) of acclimated VMPO^LepR^ neurons reduced by riluzole (10 µM) and ICA121431 (200 nM). One-way ANOVA, *P* < 0.0001; Tukey’s multiple-comparison test, ^***^P < 0.0001 (Accl.:Accl. + riluzole), ^***^P < 0.0001 (Accl.:Accl. + ICA121431); *n* = 40/10 (Accl.), *n* = 35/4 (riluzole) and *n* = 39/6 (ICA121431) cells. Right: example traces of the three conditions shown. **i**, Left: Na_V_1.3 antagonist ICA121341 blocking *I*_NaP_ in acclimated VMPO^LepR^ neurons to a similar extent to riluzole. Right: quantification of *I*_NaP_ at −35 mV based on data shown on the left. One-way ANOVA, *P* = 0.0002; Tukey’s multiple-comparison test, ^**^P = 0.0029 (Accl.:Accl. + ICA121431), ^***^P = 0.0001 (Accl.:Accl. + ICA121431 + riluzole); *n* = 8/3 (Accl.), *n* = 12/4 (ICA121431) and *n* = 10/2 (ICA121431 + riluzole). Part of the Accl. *I*_NaP_ data shown in **g** was repurposed for comparisons shown here. **j**, Distribution of temperature-insensitive, CSN, WSN and silent neurons within acclimated VMPO^LepR^ neuron populations recorded with either riluzole (10 µM) or ICA121431 (200 nM) in perfusion fluid (*n* = 33/4 for riluzole and *n* = 24/4 for ICA121431). **k**, Firing frequencies of acclimated VMPO^LepR^ control cells (*n* = 30/5), acclimated VMPO^LepR^ cells recorded with riluzole (*n* = 33/4) and acclimated VMPO^LepR^ cells recorded with ICA121431 (*n* = 24/4). Individual cells are plotted in color; black lines represent linear regression for each group *T*_core_ (slope or temperature coefficient) = 1.9 for Accl. control, *T*_core_ = 0.68 for riluzole and *T*_core_ = 0.29 for ICA121431. Acclimated control cells were randomly sampled from the acclimated VMPO^LepR^ cells plotted in Fig. 1e. Box plots in **a**–**c** and **h** represent the median and IQR; elsewhere data are shown as mean ± s.e.m (Extended Data Fig. 9 and Supplementary Figs. 1 and 2). Neuronal activity and currents were recoded under fast synaptic transmission blockade and at 36 °C.

We found that the average resting membrane potential (RMP) is depolarized by approximately 10 mV in heat-acclimated VMPO^LepR^ neurons compared with non-acclimated controls (Fig. 6a; RMP = −44.31 ± 0.85 versus RMP = −54.00 ± 1.98, *P* = 0.0001), possibly contributing to higher firing rates. In principle, a reduction of background K^+^ current can yield a more depolarized membrane potential. Rather than a decrease, we found a slight increase in overall K^+^ current in acclimated VMPO^LepR^ neurons (Supplementary Fig. 1a), suggesting that leaked K^+^ currents do not contribute to acclimation-induced RMP depolarization in a major way. This conclusion is further supported by indistinguishable membrane input resistance for the acclimated and non-acclimated groups (Fig. 6b).

Theoretically, cation-selective transient receptor potential (TRP) ion channels could pass tonic depolarizing current to increase AP firing in VMPO^LepR^ neurons. Ruthenium Red, 2-aminoethyl diphenylborinate (2-APB) and ML204—broad-spectrum inhibitors of heat-activatable TRPV1 (capsaicin receptor), TRPM2, TRPM3 (TRP subfamily M members 2 and 3) and TRPC (TRP subfamily C) channels^6,30,49,50^—had little or no effect on cation currents in acclimated VMPO^LepR^ neurons (Supplementary Fig. 1b–d). Importantly, none of the substances had a significant impact on tonic AP firing (Supplementary Fig. 1c,d). Next to TRPM2, TRPC4 channels have recently been implicated in warm-sensitive AP firing of POA neurons^30^. ML204 and Pico145, two potent inhibitors of TRPC4 channels^51^, did not attenuate tonic AP firing and warm sensitivity of heat-acclimated VMPO^LepR^ neurons, respectively (Supplementary Fig. 1d,e), suggesting that heat acclimation induces a TRPC4-independent molecular mechanism of warm sensitivity.

The sodium ‘leak’ channel NALCN^52^ has been described as modulating autonomous firing of other tonically active neurons, including suprachiasmatic nucleus (SCN) neurons that are neighboring the POA and regulate the circadian cycle^53^. We found that Na^+^ ‘leak’ currents contribute to the RMP in both non-acclimated and acclimated neurons with a significantly larger contribution in acclimated VMPO^LepR^ neurons (Fig. 6c). To determine whether the difference in RMP could explain the AP firing increase, we depolarized the non-acclimated cells to a similar membrane potential observed in acclimated VMPO^LepR^ neurons. We found that depolarization of non-acclimated cells did not have a major impact on either the frequency or the regularity of AP firing (Supplementary Fig. 1f), suggesting that mimicking a depolarized state is—on its own—insufficient to recapitulate their acclimation-induced firing pattern. Nevertheless, injecting a hyperpolarizing current into acclimated VMPO^LepR^ neurons reduced their firing rate (Supplementary Fig. 1g), showing that a depolarization bias supports tonic VMPO^LepR^ neuron activity.

As passive conductance—either K^+^ or Na^+^—did not fully explain the increased activity of acclimated VMPO^LepR^ neurons, we investigated the contribution of voltage-gated ion channels to tonic firing.

Despite the observation that fast after-hyperpolarization was changed upon acclimation (Supplementary Fig. 2a), we found that ion channels typically carrying or being activated by the underlying current, such as Ca^2+^-activated large conductance K^+^ (BK) channels and HCN (hyperpolarization-activated cyclic nucleotide-gated) channels, respectively, did not appear to contribute to acclimation-induced firing (Supplementary Fig. 2b,c). While removal of intracellular Ca^2+^ by including BAPTA in the patch pipette slightly reduced the firing frequency of acclimated VMPO^LepR^ neurons (Supplementary Fig. 2d), neither nifedipine nor mibefradil, blockers of L- and T-type voltage-gated Ca^2+^ (Ca_V_) channels, affected the firing frequency of acclimated VMPO^LepR^ neurons (Supplementary Fig. 2e) but rather we observed that the overall Ca_V_-mediated current was larger in non-acclimated VMPO^LepR^ neurons compared with acclimated cells (Supplementary Fig. 2f), arguing for a minor role of Ca_V_ channels, if any, in VMPO^LepR^ neuron pacemaking.

Voltage-gated Na^+^ (Na_V_) channels are at the core of AP initiation and upstroke. We therefore tested whether changes in Na_V_ channels could explain acclimation-induced spiking. We found the kinetic parameters of APs, such as the AP rise time and half-width, to be more rapid in acclimated VMPO^LepR^ neurons compared with controls (Fig. 6d and Supplementary Fig. 2a) and transient Na_V_ currents to be of larger amplitude (Fig. 6e), suggesting that acclimation had changed Na_V_ composition and functionality to support faster firing.

**Extended Data Fig. 9:**
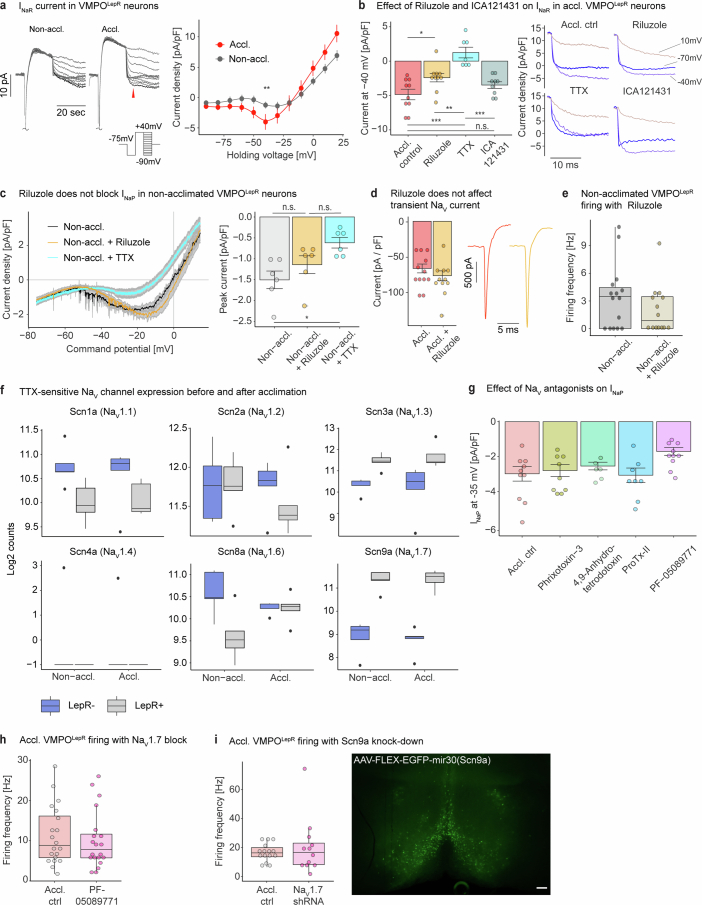
Na_V_ channel current characteristics and gene expression in VMPO^LepR^ neurons. **a**, Left: example traces of resurgent Na_V_ currents recorded in acclimated and non-acclimated VMPO^LepR^ neurons. Inset: voltage step protocol used to record the resurgent current. Right: Current-voltage relationship for VMPO^LepR^ resurgent Na_V_ current (mean ± s.e.m.). Two-way ANOVA (effect of acclimation * voltage), P < 0.0001; Tukey’s multiple comparison test, **P = 0.0041 (Non-accl.:Accl. at -40 mV). n = 10/2 (Non-accl.), n = 11/2 (Accl.) cells. **b**, Left: Riluzole reduced and ICA121431 did not affect the TTX-sensitive resurgent Na_V_ current present in acclimated VMPO^LepR^ cells. One-way ANOVA, P < 0.0001; Tukey’s multiple comparison test, *P = 0.0409 (Accl. ctrl : Riluzole), ***P < 0.0001 (Accl. ctrl : TTX), **P = 0.0033 (Riluzole : TTX), ***P = 0.0002 (ICA121431 : TTX). n = 9/2 (Accl. ctrl), n = 10/2 (Riluzole), n = 7/2 (TTX), n = 9/2 (ICA121431). Shown as mean ± s.e.m. Right: Example traces of resurgent Na_V_ currents at different potentials (-70 mV / blue, −40 mV / violet and +10 mV / beige) recorded in the acclimated condition and in the presence of Riluzole (10 µM), TTX (1 µM) or ICA121431 (200 nM). **c**, Left: Traces of I_NaP_ (mean ± s.e.m.) in non-acclimated VMPO^LepR^ neurons whereby TTX reduced the current but Riluzole did not have a significant effect. Right panel: quantification of I_NaP_ at −35mV based on presented traces. One-way ANOVA, P = 0.015; Tukey’s multiple comparison test, *P = 0.0117 (Non-accl. : Non-accl.+TTX). n = 6/2 (Non-accl.), n = 6/2 (Non-accl.+Riluzole) and n = 6/2 (Non-accl.+TTX) cells. **d**, Quantification of peak amplitude (mean ± s.e.m.) and example traces of transient Na_V_ currents recorded in acclimated VMPO^LepR^ neurons (based on the initial depolarizing step used in resurgent Na_V_ current recording protocol shown in (a)) with and without Riluzole. n = 12/2 (Accl.) and n = 11/2 (Accl. + Riluzole). Riluzole (10 µM) was found to not affect the amplitude of transient Na_V_ currents. **e**, Firing frequency of non-acclimated VMPO^LepR^ cells was not affected by Riluzole. n = 15/5 (Non-accl.) and n = 14/1 (Non-accl. + Riluzole). Boxplots represent median and interquartile range. **f**, Expression analysis of TTX-sensitive Na_V_ channels after Fluorescence-Activated Cell Sorting (FACS) of VMPO LepR^+^ and LepR^-^ cells obtained from non-acclimated and acclimated LepR-Cre;HTB mice; n = 5/3 (samples/mice); mRNA sequencing results of pooled cells are plotted as normalized log2 values. Boxplots represent median and interquartile range. **g**, Quantification of I_NaP_ amplitude (mean ± s.e.m.) at −35 mV recorded in acclimated VMPO^LepR^ neurons in the presence of the selective Na_V_ channel blockers Phrixotoxin-3 (100 nM), 4,9-Anhydrotetrodotoxin (50 µM), ProTx-II (30 nM) and PF-05089771 (150 nM). One-way ANOVA, P = 0.0471; Tukey’s multiple comparison test, P = 0.0676 (Accl.:PF-05089771). n = 10/2 (Accl.), n = 9/2 (Phrixotoxin-3), n = 6/2 (4,9-Anhydrotetrodotoxin), n = 8/2 (ProTx-II) and n = 10/2 (PF-05089771) cells. **h, i**, Neither PF-05089771 (**h**) nor Na_V_1.7 knock-down via shRNA AAV (**i**) affected AP firing frequency in acclimated VMPO^LepR^ neurons. n = 20 cells each for PF-05089771 and respective controls; n = 11 and n = 15 cells for Na_V_1.7 shRNA and controls respectively. Boxplots represent median and interquartile range. Image in (**i**) demonstrates the AAV9-pCAG-FLEX-EGFP-mir30(Scn9a) viral construct expression in LepR-cre mouse VMPO; scale bar 100 um. Brain slice recordings were conducted at 36 °C bath temperature with the exception of panel (h) where ‘low-K^+^ aCSF’ and 33 °C bath temperature were used.

Persistent (*I*_NaP_) and resurgent (*I*_NaR_) Na^+^ currents, both of which are carried by Na_V_ channels, have been associated with higher excitability and tonic pacemaker activity in several different central and peripheral neuronal populations^54–58^. Specific molecular rearrangements and the presence of certain auxiliary subunits permit tetrodotoxin (TTX)-sensitive Na_V_ channels to inject depolarizing currents during interspike intervals to drive neurons to threshold voltages, thereby inducing repetitive firing. We found that *I*_NaP_ and *I*_NaR_ in acclimated VMPO^LepR^ neurons were significantly larger than in non-acclimated controls (Fig. 6f and Extended Data Fig. 9a). Riluzole is a compound that preferentially blocks *I*_NaP_ and *I*_NaR_ but, unlike TTX, does not inhibit the transient Na_V_ current at low concentrations^59,60^. Indeed, riluzole inhibited TTX-sensitive *I*_NaP_ and *I*_NaR_ present in acclimated VMPO^LepR^ neurons; in contrast, the compound had only minimal effects on non-acclimated neurons (Fig. 6g and Extended Data Fig. 9b,c). In agreement with its reported selectivity for *I*_NaP_ and *I*_NaR_ (ref. ^60^), riluzole, at the concentration used, did not reduce transient Na_V_ currents (Extended Data Fig. 9d). Importantly, riluzole significantly reduced tonic AP firing in acclimated VMPO^LepR^ neurons, but had no significant effect on slowly firing non-acclimated neurons (Fig. 6h and Extended Data Fig. 9e), demonstrating that *I*_NaP_ and/or *I*_NaR_ contributes substantially to acclimation-induced pacemaking.

Among the different TTX-sensitive Na_V_ channels that could generate *I*_NaP_ and *I*_NaR_, we found five of the six corresponding α subunits, Na_V_1.1–Na_V_1.3 and Na_V_1.6–Na_V_1.7, to be expressed in VMPO^LepR^ neurons (Extended Data Fig. 9f). Pharmacological profiling, using semi-selective inhibitors targeting Na_V_1.7 (proToxin-II and PF-05089771), Na_V_1.6 (4,9-anhydro-tetrodotoxin, a TTX derivative that displays some crossinhibitory potential on Na_V_1.1 (ref. ^61^)) and Na_V_1.2 (phrixotoxin-3), ruled out these channels as major contributors to *I*_NaP_ in acclimated VMPO^LepR^ neurons (Extended Data Fig. 9g). Na_V_1.7 has been implicated in plastic changes of hypothalamic neurons^62^. However, Na_V_1.7 inhibition did not significantly affect Na_V_ currents or acclimation-induced AP firing (Extended Data Fig. 9g,h). In addition, RNA knock-down, specifically in VMPO^LepR^ neurons using LepR-Cre mice in combination with previously published viral AAV-shRNA particles targeting Na_V_1.7 (ref. ^62^), also did not affect tonic firing of acclimated VMPO^LepR^ neurons (Extended Data Fig. 9i). Only ICA121431, an antagonist of Na_V_1.3 and Na_V_1.1 channels^63^, substantially reduced *I*_NaP_ in VMPO^LepR^ neurons (Fig. 6i). As Na_V_1.1 is also inhibited by 4,9-anhydro-tetrodotoxin^61^, an antagonist that did not show any effect on *I*_NaP_ (Extended Data Fig. 9g), we concluded that Na_V_1.3 is the more likely candidate of the two Na_V_ subtypes inhibited by ICA121431 and relevant for generating acclimation-induced *I*_NaP_.

The effect of ICA121431 on *I*_NaP_ was similar (and nonadditive) to that observed for riluzole (Fig. 6i). ICA121431 also reduced tonic AP firing of VMPO^LepR^ neurons (Fig. 6h) and, as opposed to riluzole, had a negligible effect on *I*_NaR_ (Extended Data Fig. 9b). Importantly, both riluzole and the Na_V_1.3 blocker robustly reduced acclimation-induced warm sensitivity of VMPO^LepR^ neurons (Fig. 6j,k).

Collectively, these pharmacological experiments suggest that Na_V_1.3-driven *I*_NaP_, but not *I*_NaR_, is a major contributor to VMPO^LepR^ warm-sensitive pacemaking.

### Na_V_1.3 drives tonic warm-sensitive activity of VMPO^LepR^ neurons

**Fig. 7:**
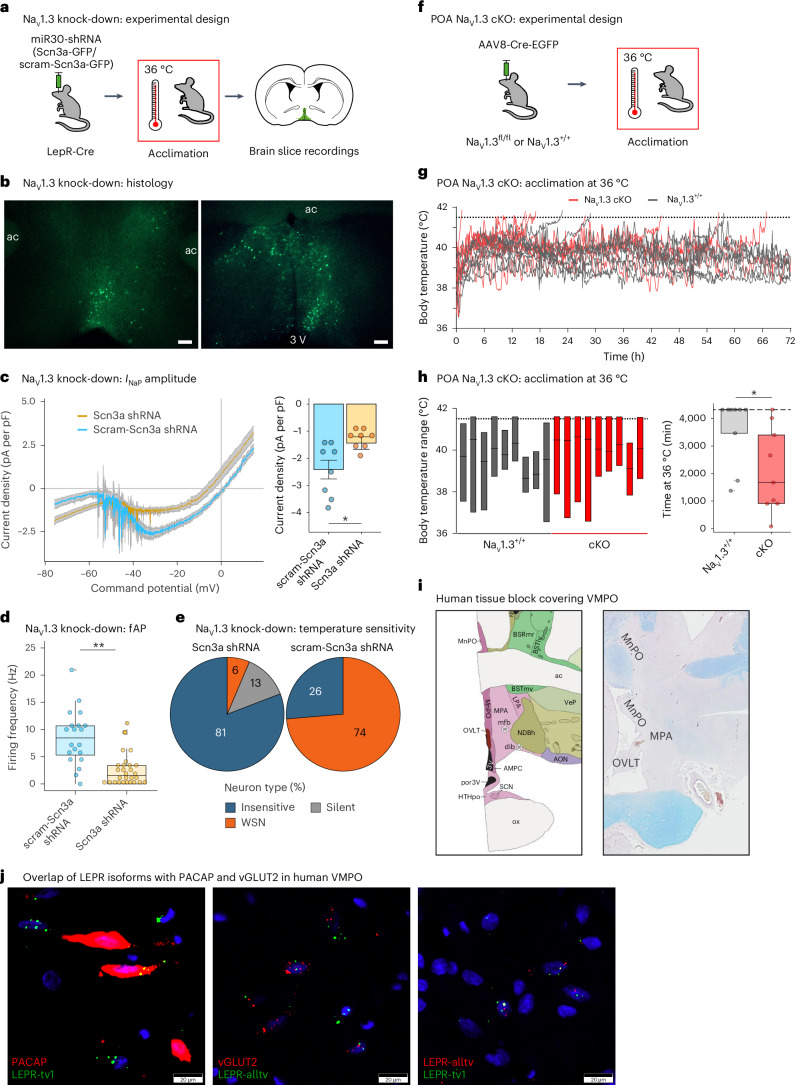
Na_V_1.3 is required for acclimation-induced, tonic warm-sensitive firing and heat tolerance. **a**, LepR-Cre mice POA injected with Cre-dependent constructs encoding shRNAs against Scn3a or scrambled control (scram-Scn3a shRNA). **b**, Acclimated VMPO^LepR^ neurons labeled with GFP encoded within the shRNA constructs. Scale bars, 250 μm. ac, anterior commissure. **c**, Left: average *I*_NaP_ in VMPO^LepR^ neurons expressing Scn3a shRNA and scrambled control. Traces are presented as mean ± s.e.m. Right: quantification (mean ± s.e.m.) of *I*_NaP_ at −35 mV, showing a reduction of *I*_NaP_ in Scn3a shRNA expressing acclimated LepR^+^ neurons. Unpaired, two-tailed Student’s *t*-test, ^*^P = 0.0174; *n* = 8/4 (Scn3a shRNA) and *n* = 8/3 (scram-Scn3a shRNA) cells. **d**, Firing frequency of acclimated VMPO^LepR^ neurons significantly reduced by the functional shRNAs. Unpaired two-tailed Student’s *t*-test, ^**^P = 0.0044; *n* = 30/5 (Scn3a shRNA) and *n* = 20/3 (scram-Scn3a shRNA) cells. **e**, Distribution of temperature-insensitive, WSN and silent neurons within the acclimated VMPO^LepR^ neuron population expressing either Scn3a (*n* = 47/5) or scram-Scn3a (*n* = 19/3) shRNA. **f**, Na_V_1.3^fl/fl^ and WT controls were injected with an AAV encoding the Cre recombinase into the POA (cKO). **g**, Most of the WT animals able to defend their body temperature within physiological range. In contrast, all but one of the Na_V_1.3 cKO animals were unable to maintain their body temperature <41.5 °C (*N* = 9 for each group). **h**, Left: range of body temperatures of animals shown in **g**. Right: quantification of endurance time at 36 °C acclimation temperature of mice shown in **g**. Cut-off time was 72 h (dashed line). Mann–Whitney *U*-test, ^*^P = 0.0155 (*N* = 9 each). **i**, Left: Allen Brain Atlas annotation of human POAs. Right: human tissue block covering POAs MnPO/MPA/OVLT (LFB/H&E stain). **j**, LEPR coexpression in human VMPO with RNAscope ISH. Left: PACAP + LEPR-tv1 (long isoform; coexpression in yellow). Middle: vGLUT2 + LEPR-alltv (all isoforms; coexpression in yellow). Right: LEPR-alltv + LEPR-tv1 (coexpression in yellow). Electrophysiological recordings were performed with fast synaptic transmission blockade and at 36 °C. Box plots show the median and IQR (Extended Data Fig. 10 and Supplementary Figs. 4 and 5).

**Extended Data Fig. 10:**
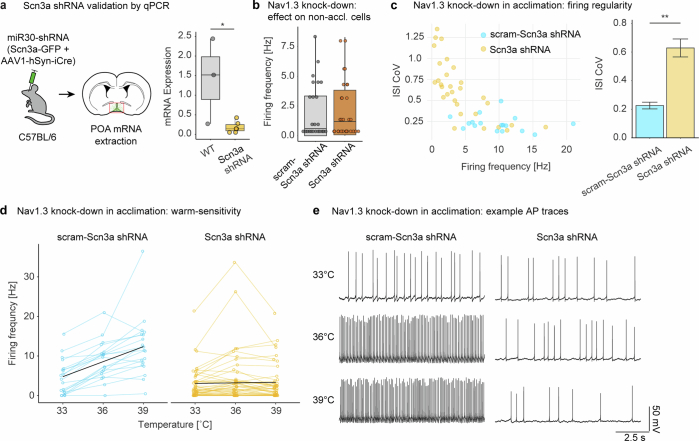
Validation and electrophysiological characterization of Na_v_1.3 knock-down. **a**, Left: in order to test the efficacy of shRNA against the Na_V_1.3 mRNA, Cre-dependent shRNA-carrying AAVs were co-injected together with AAV encoding the Cre recombinase into the POA of C57/BL6 mice. Following 3 weeks of virus expression, mRNA was extracted from the POA tissue. Non-injected C57BL/6 mouse POA tissue served as control. Right: boxplot (median and interquartile range) of relative Na_V_1.3 mRNA expression normalized to the housekeeping genes Tubb3 and Ube2l3 in mouse POA. Unpaired two-tailed t-test, *P = 0.0226. N = 3 (WT) and N = 6 (Scn3a shRNA) mice. **b**, Firing frequencies of non-acclimated VMPO^LepR^ neurons expressing either scrambled shRNA (scram-Scn3a shRNA; n = 27/2) or functional shRNAs against Nav1.3 (Scn3a shRNA; n = 27/3) at 36 °C. Shown as median and interquartile range. **c**, Left: plot showing AP firing frequency vs regularity of firing in VMPO^LepR^ neurons expressing either functional shRNAs against Na_V_1.3 / Scn3a mRNA or scrambled control. Right: quantification of the firing regularity between the two groups (plotted as mean ± s.e.m.). Unpaired two-tailed t-test, *P = 0.0226. n = 30/5 (Scn3a shRNA) and n = 17/3 (scram-Scn3a shRNA) cells. **d**, Firing frequencies of acclimated VMPO^LepR^ neurons expressing either scrambled shRNA (scram-Scn3a shRNA; n = 19/3) or functional shRNAs against Na_V_1.3 (Scn3a shRNA; n = 47/7) at the three indicated bath temperatures. Individual cells are plotted in color; black lines represent linear regression for each group. Slope=temperature coefficient (T_C_) = 1.2651 for scram-Scn3a shRNA and T_C_ = 0.0474 for Scn3a shRNA, demonstrating that Na_V_1.3 knock-down significantly reduced warm sensitivity of acclimated VMPO^LepR^. **e**, Example traces of spontaneous warm-sensitive activity of acclimated VMPO^LepR^ expressing either scrambled or functional shRNAs against Na_V_1.3 recorded at 33 °C, 36 °C and 39 °C.

To further investigate the role of Na_V_1.3 in VMPO^LepR^ neuron activity and heat tolerance, we used an RNA interference-mediated knock-down strategy, similar to that used for Na_V_1.7 above, and we developed AAV vectors for Cre-dependent, cell-type-specific knock-down of Na_V_1.3 in VMPO^LepR^ neurons (Fig. 7a,b). We confirmed Na_V_1.3 knock-down by quantitative (q)PCR (Extended Data Fig. 10a). Indeed, the amplitude of *I*_NaP_ was reduced in acclimated VMPO^LepR^ neurons when Na_V_1.3 was knocked down, but not when scrambled control small hairpin (sh)RNA was used (Fig. 7c). Moreover, warm sensitivity was also strongly and significantly reduced by Na_V_1.3 knock-down, whereas baseline excitability of non-acclimated neurons was not affected (Fig. 7d,e and Extended Data Fig. 10b–e), further strengthening the association between the *I*_NaP_ and acclimation-induced VMPO^LepR^ neuron firing properties.

To assess the role of Na_V_1.3’s enhanced functionality in acclimation and heat tolerance in vivo, we employed Na_V_1.3^fl/fl^ mice to conditionally delete the *Scn3a* gene^64^. Homozygous floxed mice and wild-type controls were preoptically injected with Cre recombinase-encoding AAVs (Fig. 7f and Supplementary Fig. 3). After verification of Cre-mediated *Scn3a* gene deletion in the POA (Supplementary Fig. 4a,b), we first assessed baseline *T*_core_ of the animals at room temperature (RT; 23 °C) and found it to be indistinguishable between the conditional POA-Na_V_1.3^−/−^ mice and the control group (Supplementary Fig. 4c). Strikingly, all but one of the POA conditional Na_V_1.3 knock-out (cKO) mice failed already during heat acclimation at 36 °C and the animals reached the critical cut-off *T*_core_ of 41.5 °C approximately twice as fast as AAV-Cre-injected wild-type controls (Fig. 7g,h). It appeared that AAV-Cre infection, broadly covering the mouse POA, had some effect in wild-type animals, slightly hampering their ability to acclimate, albeit to a lesser extent than in mice lacking the Na_V_1.3 channel. These data provide further genetic evidence that preoptic Na_V_1.3 plays an important role in heat acclimation and tolerance.

Given the critical role of VMPO^LeprR^ neurons in heat acclimation and heat tolerance in mice, we wondered whether this population of neurons, which are part of so-called ‘QPLOT’ neurons mediating heat loss responses^9^, also exists in humans. To this end, we carried out multiplex in situ hybridization (ISH; RNAscope) on postmortem human brain tissue encompassing the human VMPO (based on anatomical annotations from the adult human Allen Brain Atlas of MnPO, medial preoptic area (MPA) and the closely neighboring organum vasculosum laminae terminalis (OVLT) region: https://atlas.brain-map.org). Indeed, we were able to detect a subset of neurons within the human VMPO to express overlapping ‘QPLOT’ marker genes, including the leptin receptor, *PACAP*, *OPN5* and *PTGER3* (Fig. 7i,j and Supplementary Fig. 5).

In summary, our work emphasizes an acclimation-induced plasticity mechanism involving a persistent Na^+^ current—carried largely by Na_V_1.3—that drives warm-sensitive AP firing in VMPO^LepR^ neurons to promote heat tolerance.

## Discussion

Intrinsic warm sensitivity has been used as a defining functional parameter for a subset of POA neurons for many decades^65,66^. To what extent this feature is physiologically relevant has been a matter of debate^16^. Recent studies suggest that, in mice living under normal—coolish—housing conditions^67^, experimental POA heating has only a small effect on body temperature regulation^27,30^, arguing for modest relevance of POA heat sensitivity in rodents under these conditions. In the present study, we showed that long-term heat acclimation plastically transforms VMPO neurons to become spontaneously active, highly warm-sensitive neurons. Their gained activity is critically important to drive heat tolerance and to trigger heat loss responses in hot environments.

It is interesting to note that, in vivo, the increase in acclimation-induced warm responsiveness is recruited from the cold-responsive neuron (CRN) population, whereas, in brain slices, it is recruited from temperature-insensitive neurons. Cold sensitivity has been suggested to largely stem from synaptic connectivity rather than constituting a neuron-intrinsic property^26^. Given the high degree of reciprocal (local and long-range) POA connectivity—which is largely absent in ex vivo slice preparations—it is therefore conceivable that heat acclimation additionally induces changes in the strength of synaptic connections, thereby contributing to the robust induction of WRNs from the pool of CRNs.

Heat acclimation modulates energy metabolism and promotes loss of body weight^33^. On the other hand, perturbed energy metabolism and obesity negatively affect heat acclimation^68^. Leptin, a major signaling indicator of energy metabolism status, has been implicated in thermoregulation and body temperature increases^41,69,70^. Parallel to a reduction in body weight, we observed a drop in leptin levels in heat-acclimated animals. In line with a subtle role of leptin to modulate heat acclimation, we found that leptin supplementation during heat acclimation slightly reduced AP firing frequencies of VMPO^LepR^ neurons. However, leptin supplementation neither affected VMPO^LepR^ neuron warm sensitivity nor the animals’ performance in the heat endurance assay. This, along with our in vivo imaging data, indicates that the acquired warm sensitivity—rather than a general tonic activity increase—probably mediates enhanced heat tolerance in acclimated animals on a heat challenge.

Our study suggests that the LPBN, which processes and relays peripheral temperature information to thermoregulatory POA neurons^13,14,17^, is critical at the beginning of heat acclimation but dispensable at later stages. These two phases—phase I (thermoafferent or LPBN driven) and phase II (driven by spontaneous, temperature-sensitive VMPO^LepR^ neuron activity)—may very well coincide with the two phases of heat acclimation that have been described based on transcriptional profiling studies in rodents^31^.

After heat acclimation, chemogenetic inhibition of VMPO^LepR^ neurons had a robust effect and dramatically reduced heat tolerance, which contrasts with the effect observed when inhibiting LPBN (Fig. 3e,f and Extended Data Fig. 7d). These data suggest that the low activity of VMPO^LepR^ neurons before heat acclimation is not a major contributing factor to baseline heat tolerance, a task possibly performed by other or parallel thermoafferent pathways. However, on heat acclimation, VMPO^LepR^ neurons gain dominance and peripheral thermoafferent pathways have a reduced influence on heat tolerance.

This peripheral → central shift in thermoregulatory control can be explained by a transfer of intrinsic warm sensitivity to VMPO^LepR^ neurons on heat acclimation, which we find not only in ex vivo brain slice recordings but also in in vivo imaging experiments. Given that (1) synaptic blockers don’t affect warm sensitivity in slice recordings, (2) inhibition of LPBN → POA thermoafferent pathways appears to have little impact subsequent to heat acclimation and (3) an elevation in *T*_core_ caused by environmental heat challenges is directly detectable in the POA^27,30^ suggests that VMPO^LepR^ gain intrinsic cell-autonomous warm sensitivity and may become independent of thermoafferent synaptic drive. We presume that the newly gained warm sensitivity allows robust detection of *T*_core_ and permits VMPO^LepR^ neurons to keep *T*_core_ in check at dangerously high ambient temperatures. This transformation may reduce VMPO receptivity to peripheral—anticipatory^16^—heat detection, which presumably is of lesser importance when ambient temperatures are permanently high and affect *T*_core_.

We hypothesize that VMPO^LepR^ neurons’ tonic warm sensitivity, which we find develops over many days during heat acclimation, drives the adaptation of peripheral organs involved in thermoregulation. Whether acclimation-relevant parameters of multiple organs—including heart rate or weight, basal metabolic rate, brown adipose tissue (BAT) activity or capacity, cutaneous heat dissipation or insulation, water balance and others^33–35^—are orchestrated by VMPO^LepR^ neuron activity or whether only a subset of adaptations is VMPO^LepR^ neuron dependent is currently unknown.

The ion channels TRPM2 and TRPC4 have previously been proposed to detect acute temperature changes in the POA^27,30,48^. However, pharmacological blockade of either ion channel did not significantly inhibit spontaneous warm-sensitive AP firing of acclimated VMPO^LepR^ neurons. As TRPM2 was shown to constitute a molecular temperature sensor in presynaptic terminals, we did not expect the channel to drive cell-autonomous, warm-sensitive AP firing in VMPO^LepR^ neurons. It is possible that acclimation induces a warm-sensitivity mechanism in VMPO^LepR^ neurons that is distinct from the molecular mechanism(s) utilized by canonical WSNs.

We implicate background and voltage-gated Na^+^ currents in the acclimation-induced tonic pacemaker activity and warm sensitivity. Induced pacemaker activity is a feature that is similar to hypothalamic neurons residing in the SCN which is important for circadian clock function: during the daytime the circadian cycle, background and voltage-gated Na^+^ currents are induced to increase tonic AP firing of SCN neurons relevant for circadian homeostasis^53,71^. Intriguingly, AP firing of some SCN neurons is also temperature sensitive^72^.

Genetic perturbation as well as pharmacological inhibition of Na_V_1.3 channels not only reduced the tonic activity of acclimated VMPO^LepR^ neurons but also abrogated their warm sensitivity, suggesting that the two features are mechanistically linked. However, it is unclear whether Na_V_1.3 channels convey warm sensitivity directly or whether another molecular mechanism modulates its pacemaker activity in a temperature-dependent manner. Na_V_1.3 channels appear to be present in murine POA already at baseline conditions and its messenger RNA level does not seem to change after heat acclimation (Extended Data Fig. 9f and Supplementary Fig. 4b). It is thus likely that Na_V_1.3 channels interplay with additional channels and/or auxiliary channel subunits may render the neurons spontaneously active and warm sensitive. Such a scenario would be similar to Na_V_1.7 channels’ reported interaction with fibroblast growth factor 13 (FGF13) which results in increased heat sensitivity in peripheral sensory neurons^73^.

Possibly, the Na_V_1.3 channel is already required by preoptic neurons to transmit temperature information early on during acclimation, potentially explaining why mice lacking the Na_V_1.3 channel in preoptic neurons already fail during the early phase of acclimation.

Although our study emphasizes the role of the Na_V_1.3 channel for acclimation-induced activity in VMPO neurons, it is important to note that the coordinated action of several different classes of channels is probably necessary to fully express the highly regular, warm-sensitive, AP firing rate increases observed in acclimated VMPO^LepR^ neurons, akin to the electrical interaction of multiple conductances in SCN pacemaker neurons^74^.

**Fig. 8:**
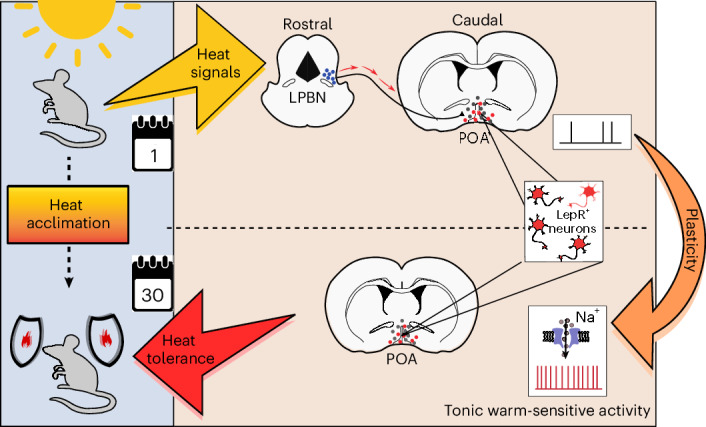
Summary. Heat stimuli reach thermoregulatory neurons in the hypothalamic preoptic area (POA) via parabrachial thermoafferent pathways (LPBN: lateral parabrachial nucleus). Sustained, long-term heat exposure triggers an adaptive process that transforms LepR-expressing POA neurons to become tonically active and warm sensitive. This form of cellular plasticity, which is mediated in part by the activity of a voltage-gated sodium channel, increases heat tolerance in mice to protect the animals from the detrimental effects of hot environments.

Our findings provide a basic molecular and cellular framework governing the central regulation of heat acclimation (Fig. 8). We anticipate that this work will pave the way to further elucidate how homeostatic pathways adapt rheostatically and whether the underlying plasticity can be utilized in medical settings, such as enhancing tolerance to hot environmental conditions.

## Methods

### Mice

The following mouse lines were used in the present study: LepR-Cre (B6.129-Leprtm3(cre)Mgmj/J, the Jackson Laboratory, IMSR cat. no. JAX:032457); PACAP-EGFP (Tg(Adcyap1-EGFP)FB22Gsat/Mmucd, MGI, cat. no. 4846839)^75^; Rosa26Lox-stop-LoxHTB (the Salk Institute for Biological Studies)^76^; Vglut2-Cre (Slc17a6tm2(cre)Lowl/J, the Jackson Laboratory, IMSR, cat. no. JAX:016963); Vgat-cre (Slc32a1tm2(cre)Lowl/J, the Jackson Laboratory, IMSR, cat. no. JAX:016962); TrpV1-cre (B6.129-Trpv1tm1(cre)Bbm/J, the Jackson Laboratory, IMSR, cat. no. JAX:017769); Rosa_DTA (Gt(ROSA)26Sortm1(DTA)Jpmb/J, the Jackson Laboratory, IMSR, cat. no. JAX:006331); FosTRAP2 (Fos2A-iCreER/+(FosTRAP2), the Jackson Laboratory, IMSR, cat. no. JAX:030323); and Na_V_1.3-floxed (B6.129S6-Scn3atm1.1Jwo/H; EMMA strain, cat. no. EM:02214). Heterozygous mice were used for experiments with the exception of the Na_V_1.3-floxed line where homozygous mice were used to create a conditional knock-out (cKO).

All animal experiments were in accordance with the local ethics committee and governing body (Regierungspräsidium Karlsruhe, Germany) and were approved under protocol nos. G-111/14, G-168/15, G-169/18, G-223/18 and G-181/21. Mice were housed at RT (23 ± 1 °C, unless specified otherwise) in an air-conditioned lab space or animal vivarium with a standard 12-h light:dark cycle and free access to food and water. All genetically modified mice in the present study were on the C57BL/6N background. All studies employed a mixture of male and female mice.

### Acclimation protocol

Mice were divided according to their acclimation status. Acclimated animals were attained by continuous exposure to 36 ± 1 °C for 24 h, 4 d or ≥4 weeks, at a humidity level of 45 ± 5%. Full heat acclimation in rodents is reached after around 4 weeks of habituating the animals to warm temperatures^19^. We therefore generally—and if not stated otherwise—used mice acclimated for 4–5 weeks, which in the present study we denote as ‘≥4 weeks’. Mice held at RT served as a control (non-acclimated) group. For heat acclimation, mice were placed in a climate chamber (Binder, cat. no. KB720) with free access to food and water. All mice were kept at the standard 12-h light:dark cycle. Mice aged between 7 and 14 weeks were used for heat acclimation. Mice were randomly assigned to the two groups.

### Heat endurance assay

Previous studies on the dynamics of acclimation reported that acclimatory homeostasis is reached after 25–30 d whereas short-term acclimation occurs after 2–3 d of acclimation^36^. At the end of acclimation period, animals were evaluated in a heat endurance assay. The heat endurance assay took place in a similar climate chamber to that used for acclimation where the ambient temperature was set to 39 °C ± 0.5 °C. Animals (aged 11–16 weeks after full acclimation) were transferred immediately from the acclimation chamber to the 39 °C chamber (always in the morning, between 09:00 and 11:00), where they took part in a heat endurance assay lasting for up to a maximum of 24 h. Similar to the acclimation period, mice had free access to food and water. The body temperature of the mice was constantly monitored for the entire period. A body temperature of 41.5 °C was used as the cut-off criterion^37,77^. At the end of the heat endurance test, animals were shortly placed back to 36 °C to avoid prolonged hypothermia and monitored until the animals were sacrificed. Mice were tested only once in the heat endurance assay and not multiple times.

In experiments where mice where supplemented with leptin during heat acclimation (and before heat endurance assay), animals were administered leptin (Peprotech, cat. no. 450-31, diluted in phosphate-buffered saline (PBS)) at an i.p. dose of 1.25 mg kg^−1^ twice daily^78^.

### General immunohistochemistry procedures

Animals were deeply anesthetized with isoflurane and transcardially perfused with PBS (3.85 g of NaOH and 16.83 g of NaH_2_PO_4_ in 1 l of distilled water) followed by a 4% paraformaldehyde (PFA) solution. Brains were dissected out and left overnight (O/N) in 4% PFA at 4 °C. Over the next 2 d, brains were immersed into PBS/sucrose solutions (24 h in 10% sucrose followed by 30% sucrose, until the brains sank to the bottom of the container tube). Brains were sectioned with a cryo-microtome at 30-μm thickness and sections (free floating) were kept in cryoprotectant solution (250 ml of glycerol and 250 ml of ethylene glycol made up to 1 l with PBS) at 4 °C until stained.

For antibody staining (ʽAntibodiesʼ), sections were washed once in PBS and left overnight at 4 °C in 0.2% Triton X-100 (PBX0.2). On the following day, sections were blocked with 5% goat serum in PBS containing 0.1% Triton X-100 (PBX0.1) for 2 h at RT. Sections were then incubated with primary antibodies, diluted in 1% goat serum in PBX0.1 for 3 d at 4 °C. On the fifth day, sections were washed extensively with PBX0.1 and then incubated with secondary antibodies and DAPI for 4 h at RT. Finally, tissue was washed extensively with PBSX0.1 and once with PBS, after which sections were mounted using Immu-Mount (Thermo Fisher Scientific, cat. no. 9990402) on to glass slides.

Confocal images were taken at the Nikon imaging center of Heidelberg University, with the Nikon A1R confocal microscope under Nikon Plan Apo λ ×10 magnification, numerical aperture (NA) 0.45 (working distance 4 mm, field of view 1.27 × 1.27 mm^2^) objective. Cell counting of cells expressing markers of interest was performed with NIS-Elements software (Nikon Instruments, Inc.) using an automatic cell-counting method. The same thresholding of the fluorescence signal was used for each of the color channels in all the quantified images. Images presented were processed with ImageJ.

### Antibodies

The following antibodies were used: chicken anti-GFP (1:1,000, Novus Biotechne, cat. no. NB100-1614); rabbit anti-c-Fos (1:1,000, Synaptic Systems, cat. no. 226 003); rabbit anti-mCherry (1:1,000, Abcam, cat. no. ab167453R); rabbit anti-SCN3A (1:700, Abcam, cat. no. ab65164); goat anti-chicken Alexa Fluor-488 (1:750, Thermo Fisher Scientific, cat. no. A-11039); goat anti-rabbit Alexa Fluor-555 (1:750, Thermo Fisher Scientific, cat. no. A-21430); and DAPI (1:10,000, Sigma-Aldrich, cat. no. 10236276001).

### TRAPping of WRNs using FosTrap2 mice

#### TRAPping of WRNs

Heterozygous FosTRAP2;HTB mice (resulting from crossing FosTRAP2 mice with the Rosa26Lox-stop-LoxHTB reporter line) were habituated in their home cages in a climate chamber (Binder, cat. no. KB720) at 23 °C and injected with saline solution on 5 d consecutively to reduce stress responses. On the day of the experiment, the climate chamber was warmed to 36 °C; 2 h into warmth exposure, *z*-4-hydroxytamoxifen (4-OHT) (see below) was delivered by i.p. injection at a dose of 50 mg kg^−1^. Mice were kept at 36 °C for another 2 or 6 h to reach a total of 4-h and 8-h TRAPping duration, respectively. Control FosTRAP2;HTB mice kept at RT (and not warmed to 36 °C) were treated in the same way (5 d consecutively of saline injections before 4-OHT injection). After the corresponding warmth exposure, both groups of animals were left at thermoneutrality (31 °C) for 48 h to prevent secondary trapping of cold-responsive cells and expecting the 4-OHT to be completely metabolized. For electrophysiology, mice were subsequently either kept at RT or acclimated at 36 °C.

#### Drug preparation

4-OH (Sigma-Aldrich, cat. no. H7904) was prepared for i.p. delivery essentially as described previously^79^ with some modifications: 4-OHT was dissolved at 20 mg ml^−1^ in ethanol by vigorous shaking at RT for 5 min + 1 min of sonication in a bath sonicator and was then aliquoted in 50-μl (1-mg) aliquots and stored at −80 °C for up to several weeks. Before use, 4-OHT was redissolved by vigorous shaking at RT for 5 min + 1 min of sonication in a bath sonicator; subsequently, 200 μl of a 1:4 mixture of castor oil:sunflower seed oil (Sigma-Aldrich, cat. nos. 259853 and S5007) was added per 50-μl aliquot containing 1 mg of 4-OHT and the ethanol:oil suspension was vigorously mixed; then, the ethanol was evaporated by vacuum under centrifugation (without heating). The final 5 mg ml^−1^ of 4-OHT solution was always used on the day of preparation. All injections were delivered intraperitoneally.

For immunohistochemistry, animals following warmth exposure were left at 31 °C until the next day. After this, all three groups (4-h TRAPped, 7-h TRAPped and control groups) were transferred to their home cages for the next 2.5 weeks. After this period, all mice were placed in a climate chamber for 24 h at 23 °C to get accustomed once more to the chamber’s environment. On the next day, the temperature in the climate chamber was adjusted to reach 36 °C to perform the classic warming challenge for hours. After the 4-h exposure to warmth, animals were sacrificed using isoflurane and transcardially perfused. POA-containing brain sections were cut at 30-μm thickness as described above. Tissue was stained for GFP and cFos to quantify the overlap of the TRAP-positive neurons (HTB/GFP positive), with neurons expressing endogenous cFos after the 36 °C warming stimulus.

### Expression of cFOS in VMPO^LepR^ neurons after exposure to 36 °C ambient temperature

To elucidate the role of VMPO^LepR^ neurons in thermoregulatory responses, we investigated whether LepR^+^ neurons are activated by acute warmth exposure. To do this, LepR-Cre mice crossed to the Rosa26Lox-stop-LoxHTB reporter line^80^ (here referred to as LepR-Cre;HTB mice) were accustomed to the climate chamber for 24 h. On the second day control animals were taken out, anesthetized with isoflurane and transcardially perfused with PBS, followed by 4% PFA.

The temperature of the climate chamber was switched to 36 °C and the experimental animals were kept at this temperature for 4 h, immediately followed by anesthesia and perfusion. Brains were dissected out and left O/N in PFA at 4 °C. Brains were immersed in sucrose solutions and sliced as described above. α-GFP and α-cFos primary antibodies were applied to amplify HTB/GFP reporter and label endogenous cFos proteins.

### Na_V_1.3 channel staining

C57BL/6 and Na_V_1.3^flox/flox^ mice were injected with AAV-Cre-GFP. After 4 weeks, to allow AAV expression and protein turnover, animals were transcardially perfused with PFA and brain tissue was processed for immunohistochemistry as described above. Then, 30-μm free-floating brain sections containing POA and cortex were stained with primary antibodies against Na_V_1.3 and GFP.

### Constructs for Scn3a knock-down

ShRNA constructs for Scn3a were developed according to the method described in ref. ^81^, with the murine Scn3a canonical complementary DNA sequence as the template. The AAV2-based CAG::FLEX-rev-hrGFP:mir30(Scn9a) vector, used previously by Branco et al.^62^, was used as a backbone after the excision of the shRNA sequence-targeting Na_V_1.7 using EcoRI and XhoI restrictases (New England Biolabs). Using the miR_Scan tool (https://www.ncbi.nlm.nih.gov/staff/ogurtsov/projects/mi30), we selected three sequences, binding to the 5ʹ-region (encoding the extracellular loop between segments 5 and 6 of domain I of the channel: sense strand sequence GAAGGACTATATCGCAGATGA), a central region (encoding the intracellular loop connecting domains II and III: sense strand sequence GTGGAGAAATACGTAATTGAT) and the 3ʹ-region (encoding segment 2 of domain IV: sense strand sequence GTCCCGAATCAACCTGGTATTT), to construct shRNAs against. Sense strands and guide strands, separated by the loop sequence TACATCTGTGGCTTCACTA, and supplemented with restriction site overhangs, were synthesized as oligonucleotides and, together with complementary oligonucleotides, aligned and cloned into the recipient vector. Such AAV vectors, where the shRNA sequences were placed between a FLEX switch sequence together with a GFP reporter gene, were packaged into AAV1/2 particles by the Viral Vector Facility, University of Zurich (Switzerland).

As a negative control for these shRNA Scn3a constructs, we produced an AAV containing a scrambled sequence (ACTGTAGTCGTCGACTTACCAT) that was subcloned into the same vector backbone as functional shRNAs.

### AAV brain injections

All surgical procedures were performed under aseptic conditions and deep anesthesia. Adult mice (7–18 weeks) were anesthetized using an i.p injection of anesthesia mix (medetomidine 0.5 mg kg^−1^, midazolam 5 mg kg^−1^ and fentanyl 0.05 mg kg^−1^). Mice were placed on a stereotaxic apparatus (Model 1900, Kopf) and kept warm using a heating pad at 33.5 °C. The fur of the head was removed, the skin disinfected (Braunol, Braun) and the cornea moisture preserved during surgery by the application of eye ointment (Bepanthen, Bayer). Craniotomies of approximately 0.5-mm diameter were drilled on the skull with a hand drill (Osada Electric, cat. no. OS40). A pulled-glass capillary with a 20- to 40-µm tip diameter was lowered into the brain and specific recombinant AAV (rAAV) carrying the functional construct or a fluorescent protein was injected using a manual air pressure system.

The following AAVs and titers were used:

- single-stranded (ss)AAV-DJ/2-hSyn1-chI-dlox-hChR2(H134R)_mCherry(rev)-dlox-WPRE-hGHp(A) (Zurich Vector Core, 5.3 × 10E12 vg ml^−1^)
- ssAAV-DJ/2-hSyn1-chI-dlox-mCherry(rev)-dlox-WPRE-hGHp(A) (Zurich Vector Core, 7.2 × 10E12 vg ml^−1^)
- ssAAV-5/2-hSyn1-chI-dlox-EGFP_2A_FLAG_TeTxLC(rev)-dlox-WPRE-SV40p(A) (Zurich Vector Core, 7.7 × 10E12 vg ml^−1^)
- ssAAV-1/2-hEF1α-dlox-hM4D(Gi)_mCherry(rev)-dlox-WPRE-hGHp(A) (Zurich Vector Core, 4.5 × 10E12 vg ml^−1^)
- ssAAV-1/2-hSyn1-chI-dFRT-EGFP_2A_FLAG_TeTxLC(rev)-dFRT-WPRE-hGHp(A) (Zurich Vector Core, 5.0 × 10E12 vg ml^−1^)
- ssAAV-1/2-hSyn1-dlox-EGFP(rev)-dlox-WPRE-hGHp(A) (Zurich Vector Core, 6.7 10E12 vg ml^−1^)
- ssAAV2/9-CAG::FLEX-rev-hrGFP:mir30(Scn9a) (a gift from S. Sternson, 1.5–1.7 10E13 GC per ml)
- ssAAV2/9-CAG::FLEX-rev-hrGFP:mir30(Scn9a-scrambled) (a gift from S. Sternson, 1.5–1.7 10E13 GC per ml)
- ssAAV-1/2-hEF1α-dlox-hM3D(Gq)_mCherry(rev)-dlox-WPRE-hGHp(A) (Zurich Vector Core, 4.0 × 10E12 vg ml^−1^)
- ssAAV-retro/2-hSyn1-chI-dlox-mCherry_2A_FLPo(rev)-dlox-WPRE-SV40p(A) (Zurich Vector Core, 6.3 × 10E12 vg ml^−1^)
- ssAAV-retro/2-hSyn1-chI-dlox-EGFP_2A_FLPo(rev)-dlox-WPRE-SV40p(A) (Zurich Vector Core, 9.9 × 10E12 vg ml^−1^)
- ssAAV-1/2-hSyn1-dFRT-hM4D(Gi)_mCherry(rev)-dFRT-WPRE-hGHp(A) (Zurich Vector Core, 8.4 × 10E12 vg ml^−1^)
- ssAAV-1/2-shortCAG-dlox-miR(Na_V_1.3-v1)(rev)-hrGFP(rev)-dlox-WPRE-SV40op(A) (Zurich Vector Core, 1.0 × 10E13 vg ml^−1^)
- ssAAV-1/2-shortCAG-dlox-miR(Na_V_1.3-v2)(rev)-hrGFP(rev)-dlox-WPRE-SV40op(A) (Zurich Vector Core, 8.9 × 10E12 vg ml^−1^)
- ssAAV-1/2-shortCAG-dlox-miR(Na_V_1.3-v3)(rev)-hrGFP(rev)-dlox-WPRE-SV40op(A) (Zurich Vector Core, 7.8 × 10E12 vg ml^−1^)
- ssAAV-1/2- shortCAG-dlox-miR(Na_V_1.3-scrambled)(rev)-hrGFP(rev)-dlox-WPRE-SV40op(A) (Dirk Grimm laboratory, Heidelberg University, 1.9 × 10E12 vg ml^−1^)
- ssAAV-8/2-CAG-EGFP_Cre-WPRE-SV40p(A) (Zurich Vector Core, 2.1 × 10E12 vg ml^−1^)
- ssAAV-1/2-hSyn1-chI-iCre-WPRE-SV40p(A) (Zurich Vector Core, 5.2 × 10E12 vg ml^−1^)
- ssAAV.DJ/2.hEF1α.dlox.GCaMP6f(rev).WPRE.bGHp(A) (Zurich Vector Core, 4.8 × 10E12 vg ml^−1^).

Skin was sutured with sterile, absorbable, needled sutures (Marlin, cat. no. 17241041, catgut) and mice were injected subcutaneously with carprofen at 5 mg kg^−1^ (Rimady, Zoetis). Finally, anesthesia was antagonized using a subcutaneous injection of atipamezole 2.5 mg kg^−1^, flumazenil 0.5 mg kg^−1^ and naloxone 1.2 mg kg^−1^ and mice were transferred to their home cages. For postoperative care, a second dose of carprofen was injected after 24 h and mice cages were kept on a veterinary heating pad at 37 °C for 12 h and monitored closely. A minimum of 3 weeks of viral expression was allowed before any experiments were conducted.

### Telemetry transmitter implantation

All animals (with the exclusion of those used for electrophysiological recordings) were implanted with a telemetry transmitter (Data Sciences International, cat. no. TA11TA-F10) to monitor body temperature during the acclimation procedure and behavioral testing. Animals were injected intraperitoneally with an anesthesia mix as described above, and the fur of the abdomen was removed, the skin disinfected with Braunol (Braun, cat. no. 3864065) and the cornea protected with Bepanthen ointment (Bayer). A sterile telemetric transmitter was implanted in the abdominal cavity. Thereafter, muscle and skin layers were separately sutured with absorbable surgical threads. After the surgery, the anesthesia was antagonized and animals were monitored as described above; recovery for at least 1 week was allowed before any further procedures were undertaken.

### Tail, interscapular BAT and core body temperature measurement

In ChR2-encoding, AAV-injected mice (and respective control animals), tail temperatures and BAT temperatures were measured using an infrared thermal camera (VarioCAMhr, InfraTec). Snapshot images were taken every 5 min using IRBIS 3 software (InfraTec). The average temperature was calculated in the middle of the tail (segment length of 1 cm) and at the center of the interscapular region, which was shaved 3–5 d before measurement. Core body temperature was sampled every 5 min via receiver plates (DSI, cat. no. RSC-1) placed underneath the cages. Telemetry data were registered using Ponemah (DSI). All measurements were conducted during the light phase.

### Optogenetic stimulation of LepR cells

Stereotactic surgeries were performed in adult LepR^Cre^ neurons. Animals were injected bilaterally with 250 nl of AAV encoding the Cre-dependent ChR2 or mCherry (control group) (ssAAV-DJ/2-hSyn1-chI-dlox-hChR2(H134R)_mCherry(rev)-dlox-WPRE-hGHp(A) or ssAAV-DJ/2-hSyn1-chI-dlox-mCherry(rev)-dlox-WPRE-hGHp(A)) at coordinates targeting VMPO neurons: bregma: mediolateral (ML): ±0.400 mm, anteroposterior (AP): 0.800 mm, dorsoventral (DV): −4.850 mm (VMPO). A 200-μm diameter fiberoptic probe (ThorLabs, cat. no. FT200UMT) was lowered to target the preoptic LepR cell population (coordinates: bregma: ML: 0.400 mm, AP: 0.800 mm, DV: −4.700 mm (VMPO)). The probe was anchored to the skull with dental acrylic. After the surgery, the anesthesia was antagonized and mice were transferred to their home cages. Postoperative care and telemetry implantation were performed as described above. At least 4 weeks were allowed for recovery and full expression of ChR2 before the start of optogenetic stimulation.

To activate ChR2-expressing LepR neurons, a fiberoptic probe was attached through an FC/PC adapter to a 473-nm blue light-emitting diode (LED; Optogenetics-LED-Blue, Prizmatix). All experiments were conducted unilaterally and the fiberoptic cable was connected at least 2 h before the experiments to allow for habituation. For the optogenetic probing, mice received light pulses of 4–6 mW power and 10 ms, delivered at 20-Hz stimulation frequency using a Prizmatix Pulser software and pulse train generator. In each optogenetic probing experiment, the light stimulation period was of 1 min followed by an interstimulation interval of 3 min.

### TeTxLC and Gi-DREADD silencing of LepR cells

For these experiments, stereotactic surgeries were performed in adult LepR^Cre^ mice as described in previous sections; 250 nl of rAAV encoding the Cre-dependent tetanus toxin light chain (TeTxLC) (ssAAV-5/2-hSyn1-chI-dlox-EGFP_2A_FLAG_TeTxLC(rev)-dlox-WPRE-SV40p(A)) or the inhibitory Gi-DREADDs (ssAAV-1/2-hEF1α-dlox-hM4D(Gi)_mCherry(rev)-dlox-WPRE-hGHp(A)) was injected bilaterally into the VMPO. AAVs encoding a Cre-dependent mCherry/EGFP were used as controls. After brain injection, the anesthesia was antagonized and mice were transferred to their home cages. Postoperative care was performed as described above. After telemetry implantation, heat (or RT) acclimation and heat endurance assay were performed as detailed in previous sections.

Acute chemogenetic silencing of LepR cells was performed by i.p. injection of CNO (or saline) 0.3 mg kg^−1^ (Enzo, diluted in saline) 10 min before transferring the animals to the heat endurance assay. Body temperature was constantly monitored as mentioned above. To validate CNO effects on the firing frequency of acclimated LepR cells, a group of chemogenetically silenced animals were used for in vitro electrophysiological recordings. The slice preparation and electrophysiological recording procedures are described below.

### Gq-DREADD and ChR2 stimulation and long-term activation (optogenetic and chemogenetic conditioning) of LepR cells

For these experiments, stereotactic surgeries were performed in adult LepR-Cre mice as described in previous sections; 250 nl of AAV encoding for Cre-dependent excitatory Gq-DREADDs (ssAAV-1/2-hEF1α-dlox-hM3D(Gq)_mCherry(rev)-dlox-WPRE-hGHp(A)) or Cre-dependent ChR2 (ssAAV-DJ/2-hSyn1-chI-dlox-hChR2(H134R)mCherry(rev)-dlox-WPRE-hGHp) was injected bilaterally into the VMPO.

To mimic acclimation by optogenetic activation of VMPO^LepR^ cells, ChR2-expressing mice received light pulses via the fiberoptic probe of 10-ms duration or pulse (4–6 mW), triggered at 1-Hz frequency. The stimulation was protracted continuously for the duration of the heat challenge (maximum 9 h) or was started 1 d or 3 d before heat endurance. The degree of hypothermia produced by this continuous optogenetic stimulation was tested in a different cohort of mice at RT.

For chemogenetic activation of VMPO^LepR^ cells to mimic acclimation, animals were injected daily with CNO (i.p. 0.3 mg kg^−1^, Enzo, diluted in saline) for 1, 5 or 10 d consecutively. The CNO effect on their body temperature was monitored constantly. At the end of the injection period, and 24 h from the last injection, animals were tested in the heat endurance assay while the temperature was recorded telemetrically as described above. A group of long-term, chemogenetically stimulated animals were used for electrophysiological recordings.

Repeated administration of CNO to control mice (in the absence of Gq-DREADD) over a period of 10 d also had a small but discernible effect, in particular on the kinetics of *T*_core_ at the initial phase of the heat endurance assay, possibly reflecting the activity of CNO metabolites, such as clozapine, known to modulate several neuronal receptor systems^82,83^. Nevertheless, this effect was considerably smaller than that observed in mice carrying the chemogenetic activator Gq-DREADD. Of note, all mice carrying Gq-DREADD and chemogenetically conditioned for 10 d reached the cut-off time in the heat endurance assay, suggesting that their gained heat tolerance—and different to that of any of the other experimental groups—was underestimated in this assay (Extended Data Fig. 9c,d).

### Silencing of LPBN neurons

Vglut2-Cre mice were injected with 250 nl of retroAAV encoding the Cre-dependent FlpO recombinase (ssAAV-retro/2-hSyn1-chI-dlox-mCherry_2A_FLPo(rev)-dlox-WPRE-SV40p(A) or ssAAV-retro/2-hSyn1-chI-dlox-EGFP_2A_FLPo(rev)-dlox-WPRE-SV40p(A)) bilaterally into the VMPO. After 3 weeks, the same animals received 250 nl of a bilateral injection of AAV encoding the FlpO-dependent TeTxLC or the inhibitory Gi-DREADD (ssAAV-1/2-hSyn1-chI-dFRT-EGFP_2A_FLAG_TeTxLC(rev)-dFRT-WPRE-hGHp(A) or ssAAV-1/2-hSyn1-chI-dFRT-EGFP_2A_FLAG_ hM4D(Gi)(rev)-dFRT-WPRE-hGHp(A)) at bregma: ML: ±1.25 mm, AP: −4.900 mm, DV: −2.7 mm (LPBN). After telemetry implantation, heat acclimation was performed as detailed earlier.

Acute chemogenetic silencing of LPBN presynaptic partner cells was performed by i.p. injection of CNO (or saline) 0.3 mg kg^−1^ (Enzo, diluted in saline) at the end of the acclimation protocol and 10 min before transferring the animals to the heat endurance assay. Cre-negative animals were subjected to the same injection procedure and served as controls. Body temperature was constantly monitored for all animals during the acclimation period and/or the heat endurance assay.

### Na_V_1.7 or Na_V_1.3 knock-down in VMPO^LepR^ cells

Animals were anesthetized as described above and the shRNA virus against Scn9a or Na_V_1.7 (rAAV2/9-CAG::FLEX-rev-hrGFP:mir30(Scn9a)) or scrambled control (rAAV2/9-CAG::FLEX-rev-hrGFP:mir30 (Scn9a-scrambled))^62^ or against Scn3a or Na_V_1.3 (ssAAV-1/2-shortCAG-dlox-miR(Na_V_1.3-v1/v2/v3)(rev)-hrGFP(rev)-dlox or scrambled control ssAAV-1/2-shortCAG-dlox-miR(Na_V_1.3-scrambled)(rev)-hrGFP(rev)-dlox) was injected into the POA to target LepR^+^ neurons (250 nl, bilaterally). The three Na_V_1.3-targeting shRNA AAVs (v1, v2 and v3) were mixed at 1:1:1 proportions before injections. After recovery and acclimation, animals were used for in vitro electrophysiological recordings.

### Na_V_1.3 cKO

To create Na_V_1.3 cKO, Na_V_1.3-floxed mice were brought to homozygosity (Na_V_1.3^fl/fl^) and injected with Cre-encoding AAV (AAV8 CAG EGFP-Cre) into VMPO. Wild-type mice (Na_V_1.3^+/+^) were injected with Cre-encoding AAV to serve as controls. At least 3 weeks of virus expression or protein turnover was allowed before subjecting the cKO mice and controls to heat acclimation. A mix of Cre-AAV-injected wild-type (WT) littermates and WT C57BL/6 mice was used as a control group.

### Histology of AAV-injected mouse brains

Mice were anesthetized, transcardially perfused with PFA and decapitated. The entire heads were left in 4% PFA for at least 1 d at 4 °C. Subsequently, the brains were removed from the skull and transferred to PBS containing sucrose. Coronal sections of 30 µm were cut at the microtome and stored at −20 °C in cryoprotectant solution. Subsequently, brain sections were stained for GFP or mCherry as described previously.

### LepR cell dissociation (for RNA-seq)

Adult mice LepR-Cre;HTB, acclimated and non-acclimated (10–12 weeks of age), were anesthetized with isoflurane and decapitated. The brain was immediately removed and submerged in ice-cold artificial cerebrospinal fluid (aCSF). Three brains were sectioned at the same time on a Vibratome (Leica, cat. no. VT1200S) in a slicing chamber containing ice-cold aCSF: NaH_2_PO_4_ (1.2 mM), KCl (1.2 mM), Hepes (20 mM), glucose (25 mM), NaHCO_3_ (30 mM), *N*-methyl-d-glucamine (NMDG; 93 mM), Na ascorbate (5 mM), Na pyruvate (3 mM), *N*-acetylcysteine (12 mM), CaCl_2_ (0.5 mM) and MgSO_4_·7H_2_O (10 mM), constantly bubbled with carbogen. Brain slices of 250-μm thickness, containing the rostral POA, parts of the OVLT and MnPO, were transferred to a Petri dish containing aCSF. We implemented the neuron isolation protocol described in ref. ^20^. The regions of interest (ROIs)were micro-dissected under a dissecting microscope and transferred to a small Petri dish containing 3 ml of papain mix consisting of Hibernate mix (Hibernate-A medium (Invitrogen, cat. no. A1247501), 1× Glutamax (Gibco, cat. no. 35050-038), 0.8 mM kynurenic acid (Sigma-Aldrich, cat. no. K3375-5G), 0.05 mM AP-V (HelloBio, cat. no. HB0225), 0.01 mM Rock inhibitor Y-27632 (HelloBio, cat. no. HB2297), 1 mM B27 (Invitrogen, cat. no. 17504001), 5% trehalose (Sigma-Aldrich, cat. no. T9531-10G)) and 8 U ml^−1^ of papain (Sigma-Aldrich, cat. no. P4762), 100 U ml^−1^ of DNAse I (Worthington über Cell-Systems, cat. no. LK003172), 0.005 U ml^−1^ of chondroitinase ABC (Sigma-Aldrich, cat. no. C3667-5UN), 0.07% hyaluronidase (Sigma-Aldrich, cat. no. H2126) and 0.001 mM NaOH. The tissue was cut into smaller pieces and transferred together with the papain mix into a 2-ml tube at 37 °C to incubate while shaking (700 r.p.m.) for 2 h. After incubation the papain solution was pipetted out of the tube and exchanged with Hibernate mix containing 0.1 mg ml^−1^ of ovalbumin and centrifuged for 1 min at 300*g*. Supernatant was removed and the Hibernate mix was added to the tissue pieces, which were further dissociated into single cells by gentle trituration through Pasteur pipettes with fire-polished tip openings of 600-, 300- and 150-μm diameter. Cell suspension was centrifuged at RT and 300*g* for 10 min and the supernatant was removed and exchanged with 500 μl of Hibernate-A medium. Resuspended cell material was passed through a 20-µm filter. Cell suspension was stained with propidium iodide (PI; BD Pharmingen, cat. no. 5166211E) to exclude the dead cells before the FACS analysis. FACS sorting was performed on a BD FACS Aria II using the purity sorting mode. FACS populations were chosen to select cells with low PI and high GFP fluorescence.

Cells were FACS sorted into bulks of GFP^+^ and GFP^−^ directly into the RLT buffer (QIAGEN RNeasy Micro Kit, cat. no. 74004), according to the arbitrary levels of GFP fluorescence, immediately frozen on dry ice and stored at −80 °C. Samples were processed for a maximum of 1 month from the isolation by using the column purification method according to the manufacturer’s instructions and samples were stored at −80 °C until further processing.

### cDNA library preparation (for RNA-seq)

RNA integrity and the concentration of each sample were assessed by Agilent Bioanalyzer Nano 6000 chip (Agilent Technologies) and QUBIT (Invitrogen, cat. no. QUBIT2) measurement. We used the Smart seq2 protocol^84^ for the cDNA library preparation (all processing performed at Gene Core EMBL, Heidelberg). Then, 200 pg of each RNA bulk sample was processed for the reverse transcription (Superscript IV) followed by 18 cycles of PCR amplification, library tagmentation (Tn5 transposase produced in house, PEP Core EMBL, Heidelberg), sample barcoding and a final 12 cycles of PCR enrichment. All samples were sequenced on Illumina NextSeq 500 High sequencer, single end with 75-bp long reads (Gene Core EMBL).

The RNA sequencing (RNA-seq) results are deposited at Array Express (https://www.ebi.ac.uk/biostudies/arrayexpress) and can be found under the following accession no.: E-MTAB-14029

### POA slice preparation for electrophysiology

For in vitro electrophysiology, 8- to 15-week-old mice were deeply anesthetized using a ketamine/xylazine mixture (ketamine: 220 mg kg^−1^ (Ketavet, Zoetis) and xylazine 16 mg kg^−1^ (Rompun, Bayer)), decapitated and their brains transferred to ice-cold (4 °C) oxygenated (95% O_2_, 5% CO_2_) slicing aCSF (in mM): NaCl, 85; KCl, 2.5; glucose, 10; sucrose, 75; NaH_2_PO_4_, 1.25; NaHCO_3_, 25; MgCl_2_, 3; CaCl_2_, 0.1; myoinositol, 3; sodium pyruvate, 2; and ascorbic acid, 0.4. Coronal (250-μm thick) POA slices were prepared with a Vibratome and then incubated at 32 °C in a bath containing oxygenated holding aCSF (in mM): NaCl, 109; KCl, 4; glucose, 35; NaH_2_PO_4_, 1.25; NaHCO_3_, 25; MgCl_2_, 1.3; and CaCl_2_, 1.5. After a recovery period of 30 min, individual slices were transferred to the recording chamber where they were continuously superfused with oxygenated recording aCSF (for recipes, see below) at ~2 ml min^−1^.

In some experiments, brain slices were prepared using carbogen-bubbled NMDG–Hepes solution (at 4 °C) containing (in mM): NMDG, 93; KCl, 2.5; NaH_2_PO_4_, 1.2; l(+)-ascorbic acid, 5; thiourea, 2; sodium pyruvate, 3; MgSO_4_·7H_2_O, 10; CaCl_2_·2H_2_O, 0.5; Hepes, 20; NaHCO_3_, 30; glucose, 25; and *N*-acetyl-l-cysteine, 10 (pH 7.37–7.38, 295–305 mosmol kg^−1^). After slicing, POA coronal slices were incubated for 15 min in the same NMDG–HEPES solution at 32 °C and subsequently transferred to a chamber containing holding aCSF composed of (in mM): NaCl, 118; KCl, 2.5; NaHCO_3_, 24; NaH_2_PO_4_, 1.2; sodium pyruvate, 2.4; l(+)-ascorbic acid, 4; *N*-acetyl-l-cysteine, 2; Hepes, 5; MgSO_4_, 1; CaCl_2_, 2; and glucose, 7 (pH 7.3–7.5, 295–305 mosmol kg^−1^).

Cells in acute POA slices were visualized using a SliceScope upright microscope (Scientifica) equipped with a ×40 water immersion objective (Olympus, cat. no. U-TV1X-2). Images were acquired by a digital CCD camera (Hamamatsu Photonics K.K., ORCA-R2, cat. no. C10600-10B) using MicroManager 1.4 software (Vale’s lab, University of California San Francisco (UCSF)). Electrophysiological recordings were acquired using a MultiClamp 700B amplifier (Molecular Devices), together with an Axon Digidata 1550B digitizer (Molecular Devices) and Clampex 11.0.3 software (Molecular Devices). All signals were sampled at 20 kHz and low pass filtered at 10 kHz. Borosilicate glass micropipettes used (outer diameter 1.5 mm, inner diameter 0.86 mm; Sutter Instrument, cat. no. BF150-86-7.5) were pulled on a micropipette puller (Sutter Instrument, cat. no. P-97). Intracellular solution was passed through a 0.22-µm filter before filling the electrode pipette. The open pipette resistance was between 4 MΩ and 8 MΩ.

### Electrophysiological measurement of warmth sensitivity of VMPO neurons

In acute slice experiments where neuronal action potentials were recorded at varying temperatures, a bridge in a form of glass capillary filled with agar dissolved in 3 M KCl was placed between the bath chamber and the ground electrode to isolate the reference electrode from the temperature changes applied to the chamber^85^. Equipment for bath temperature control consisted of temperature-controlled microscope stage (Luigs & Neumann, cat. no. TC07), an in-line heater (Warner, cat. no. CL-100) and a liquid cooling system (Warner, cat. no. LCS-1).

Neuronal action potentials were recorded with aCSF containing (in mM)—NaCl, 125; KCl, 6.25; glucose, 15; NaH_2_PO_4_, 1.25; NaHCO_3_, 25; MgCl_2_, 1.3; and CaCl_2_, 2.4 (called ‘high-K^+^ aCSF’) as previously described^85^—and with an internal solution containing (in mM): K gluconate, 138; KCl, 2; NaCl, 5; Hepes, 10; (ethylenebis(oxonitrilo))tetra-acetate (EGTA), 10 (or equimolar amount of BAPTA); CaCl_2_, 1; and Mg-ATP, 1.

AP frequencies were analyzed in traces where the bath temperature was 33, 36 or 39 °C; a deviation of a maximum ±0.5 °C was tolerated. Neurons were classified as warm sensitive (WSN) when their temperature coefficient reached 0.75 Hz per °C and as cold-sensitive (CSN) when their temperature coefficient was lower than −0.6 Hz per °C, thresholds traditionally used to define central temperature-sensitive neurons^86^. Temperature-insensitive neurons had their temperature coefficient between ≥−0.6 Hz per °C and <0.75 Hz per °C and neurons were classified as silent when not a single spontaneous AP could be detected. Cells unable to produce AP even when stimulated with current injection were excluded from analysis. Probing VMPO neuronal populations for temperature sensitivity was done in the presence of synaptic blockers (gabazine 5 µM, CNQX 10 µM and AP-V 50 µM) added to the bath solution. In experiments where the effect of cholinergic transmission was tested, 10 μM tubocurarine and 10 μM scopolamine were included in the perfusion fluid.

In some experiments, APs were measured without varying bath temperature (at 33 °C) and with a ‘low-K^+^ aCSF’, containing (in mM): NaCl, 125; KCl, 2.5; NaHCO_3_, 24; NaH_2_PO_4_, 1.2; Hepes, 5; MgSO_4_, 1; CaCl_2_, 2; and glucose, 8. The solution used and temperature of recordings are indicated in the figure legends showing spontaneous AP firing data.

### Recordings of ionic currents

The RMP was measured in the current-clamp mode using extracellular solution containing (in mM): NaCl, 150 (or equimolar amount of NMDG); KCl, 3.5; Hepes, 10; glucose, 20; CaCl_2_, 1.2; and MgCl_2_, 2 (as per ref. ^87^). TTX (0.5 μM) was added to the aCSF and pipette solution contained (in mM): K gluconate, 120; Hepes, 40; MgCl_2_, 5; Na_2_ATP, 2; and Na-GTP, 0.3.

To record voltage-ramp responses in voltage-clamp mode to approximate passive membrane permeability to potassium, we used a low-sodium and 0 mM nominal calcium solution that contained (in mM): NMDG, 125; NaHCO_3_, 24; KCl, 2.5; NAH_2_PO_4_, 1.2; Hepes, 5; glucose, 8; and MgSO_4_, 1. The pipette solution contained (in mM)—cesium methanesulfonate, 120; Hepes, 40; MgCl_2_, 5; Na-ATP, 2; Na-GTP, 0.3; QX-314, 5; tetraethylammonium chloride (TEAC), 5; and 4-aminopyridine (4-AP), 1—to block voltage-gated potassium and sodium channels. ‘Leak’ potassium channels are largely unaffected by intracellular cesium^88^.

Voltage ramps as well as voltage-gated calcium currents were recorded using the same cesium methanesulfonate-based pipette solution and external solution composed of (in mM): NaCl, 125; NaHCO_3_, 24; KCl, 2.5; NaH_2_PO_4_, 1.2; Hepes, 5; glucose, 8; MgSO_4_, 1; and CaCl_2_, 2. To record voltage-gated calcium currents, aCSF additionally contained 0.5 μM TTX, 1 mM TEAC and 100 μM 4-AP.

To measure voltage-gated sodium currents in whole-cell and nucleated patch configurations, we used solutions as described in ref. ^89^. In the present study, external solution contained (in mM): NaCl, 124; KCl, 3; glucose, 30; NaH_2_PO_4_, 0.5; NaHCO_3_, 25; MgSO_4_, 1; and CaCl_2_, 1.5; with the addition of TEAC (5 mM) and CdCl_2_ (50 μM). The pipette solution contained (in mM): Cs gluconate, 100; NaCl, 4; TEAC, 10; 4-AP, 5; EGTA, 10; CaCl_2_, 1; Hepes, 10; Mg-ATP, 4; Na-GTP, 0.3; and Na phosphocreatine, 4.

Spontaneous synaptic currents were recorded with ‘low-K^+^ aCSF’ and cesium methanesulfonate-based pipette solution. The spontaneous excitatory postsynaptic currents were recorded while holding the neurons at −65 mV in gap-free mode; The spontaneous inhibitory postsynaptic currents were recorded at the potential of 0 mV (reversal potential for α-amino-3-hydroxy-5-methyl-4-isoxazolepropionic acid currents).

In vitro validation of DREADD receptor function was performed in current-clamp mode using low-K^+^ aCSF, a K gluconate-based intracellular solution as described above and with the addition of 5 µM CNO.

For AP frequency quantification, the first 3 min of recordings were omitted in voltage-clamp recordings and at least 1 min was allowed after break-in before any recording was performed. All ionic current recordings were conducted at 36 °C (±0.5 °C) to mimic more closely the physiological neuronal conditions. Basic cell membrane properties such as capacitance and input resistance were calculated based on a membrane test protocol (a brief step of −10 mV from a holding potential of −65 mV). Series resistance (*R*_s_) was typically 10–25 MΩ across experiments. In voltage-clamp recordings, whole-cell capacitance compensation was applied and *R*_s_ values were compensated 50–60%; the compensation was readjusted before each protocol. The voltage protocols applied are shown in the insets to the Extended Data Figs. In current-clamp experiments, pipette capacitance neutralization and bridge balance were used. In experiments where voltage-gated sodium currents were measured, a liquid junction potential (LJP) of 8 mV was corrected online; in other experiments, the LJP was corrected offline. In voltage-clamp experiments, cells with a membrane resistance changed by >50% or *R*_s_ values changed by >20% between the start and end of the recording were excluded from analysis. All electrophysiology data were acquired with pClamp 10 and pClamp 11 software (Molecular Devices). An in-house software was developed for the automated analysis of the AP waveforms. Cells were chosen for patch-clamp recordings on a random basis, provided that they were within the specified brain region and had a healthy cell membrane.

### Quantitative PCR

The animals were sedated with isoflurane and sacrificed via cervical dislocation 3 weeks after injection of shRNA AAVs to the POA. The whole brain was prepared and stored in cold Dulbecco’s PBS (Gibco). The brain was cut with the help of a mouse brain matrix and the whole POA was extracted and transferred to an Eppendorf tube, which was subsequently filled with TRIzol reagent. RNA was extracted using the TRIzol (Ambion, cat. no. 15596026) and ROTI phenol/chloroform/isoamyl alcohol (Carl Roth, cat. no. A156) protocol. The POA tissue was transferred from TRIzol solution to a glass mortar and manually disrupted with a pestle. Subsequently, disrupted tissue was suspended in ROTI phenol/chloroform/isoamyl alcohol. The samples were centrifuged at 208 r.c.f. (relative centrifugal force) in a tabletop centrifuge at 4 °C for 10 min. The resulting aqueous phase was transferred to a spin column for purification (Zymo Research, cat. no. R1013) and the eluted RNA was stored at −80 °C until further analysis.

Total RNA, 600 ng, was used for first-strand cDNA generation with SuperScript III Reverse Transcriptase (Thermo Fisher Scientific, cat. no. 10368252) using oligo(dT) primers according to the manufacturer’s instructions. The resulting cDNA was diluted to a concentration of 600 ng µl^−1^. The cDNA was analyzed by qPCR using the following primers specific for the *Scn3a* transcript. *Ube2l3* and *Tubb3* served as housekeeping genes. Primer sequences are listed here:

**Table:** 

**Gene**	**Primer sequence**
*Scn3a*	F: GTGGACCTGGGCAATGTCT
	R: CACGATGGTCTTTAAACCTGGAA
*Ube2l3*	F: CAGCAGCACCAGATCCAAGA
	R: GGTTGTCAGGAACAATAAGCCC
*Tubb3*	F: TGAGGCCTCCTCTCACAAGT
	R: GTCGGGCCTGAATAGGTCTC

The qPCR amplification reactions (15 µl) contained 7.5 µl of FastStart Essential DNA Green Master Mix (Roche, cat. no. 06402712001), 5 µl of RNase-free water (QIAGEN), 1 µl of cDNA and 1.5 µl of forward (F) and reverse (R) primer. Reactions were run on a Roche LightCycler 96 System (Roche Diagnostics). Controls without reverse transcription were included to control for traces of genomic DNA. No template controls were included to check for contamination and nonspecific amplification. The resulting *C*_T_ values were exported as text files and imported into Microsoft Excel for further analysis. The acquired data were analyzed by an approach described in refs. ^90,91^. Data are expressed as relative gene expression ratios. All samples were measured in triplicates.

### Microendoscopy of VMPO^LepR^ neurons in awake behaving mice (Miniscope experiments)

Experiments were performed in 8-week-old, male LepR-Cre^+/−^ mice. Each mouse underwent two sequential stereotaxic surgeries, one for injections of an AAV vector expressing GCaMP6f (Zurich Virus Core) and a second performed 7 d later to implant a gradient refractive index (GRIN) lens attached to a baseplate.

For AAV injections, mice were deeply anesthetized with 2% isoflurane at a flow rate of 0.5 l min^−1^ and placed in a stereotactic frame (Kopf Instruments). Body temperature was maintained at 37 °C with a heating pad (Hot-1, Alascience, Scientific Instruments). Ophthalmic ointment (Bepanthen) was applied to the eyes to prevent drying. On deep anesthesia, mice underwent bilateral craniotomies at two AP locations, using a high-speed, rotary, micro-drill (Stereotaxic Drill, Kopf Instruments). The following stereotaxic coordinates were used: 0.2 mm AP ± 0.4 mm ML and 0.5 mm AP ± 0.4 mm ML. Then, a glass pipette filled with GCaMP6f delivered the virus into the VMPO (5-mm DV). In each injection site, 200 nl of a 1:3 virus dilution in saline solution was injected, using a NanoJet microinjector (World Precision Instruments) at a rate of 20 nl min^−1^. After each injection, the pipette was left at the injection site for 10 min to avoid backflow and then slowly withdrawn. The skin was then sutured with nylon suture thread (Dafilon, Braun).

At 7 d, a GRIN lens + baseplate was implanted. For this, mice were again anesthetized as previously described and a 0.6 × 7 mm^2^ GRIN lens with an integrated baseplate for the nVista, miniaturized, head-mounted microscope (Miniscope, Inscopix) was slowly inserted into the brain (60 µm min^−1^) on one of the hemispheres previously injected. The following stereotaxic coordinates were used: 0.35 mm AP, 0.4 mm ML and 5 mm DV. Then, the baseplate was fixed to the skull using a self-curing adhesive resin (Super-Bond, Sun Medical) and a light-cured composite resin (Gradia-Direct Flo, GC Corp.). The surface of the lens was then covered with a plastic basecap (Inscopix) and the skin was sutured with nylon suture thread.

Then, 6–7 weeks after implantation, the pre-acclimation recordings were performed. To dock the Miniscope to the baseplates, mice were briefly anesthetized with isoflurane (2%). On docking, the Miniscope was locked to the baseplate with a small screw. Mice recovered from anesthesia for at least 30 min, at RT, in a Plexiglass box (25 × 25 cm^2^) with bedding. Recordings of calcium signals were first performed at RT, at five different focal points. At each focal point, calcium transients were recorded for 2 min, at 20 Hz, with a maximum resolution of 1,280 × 800 pixels^2^ and an LED power of 1.0–1.1 mW mm^−2^. After this, mice were moved to the heating chamber that was maintained at close to 36 °C (±2 °C). Recordings at high temperature started 5 min after the mice were transferred into the heating chamber, using the same parameters of low temperature recordings. Once the pre-acclimation recording session finished, mice were temperature acclimated for 30 d, as described earlier. Post-acclimation recordings were performed following the same procedure as for the pre-acclimation recordings described above. In the control experiment, mice were recorded at identical time points and in identical conditions, but maintained at RT between recording sessions (30 d).

After recordings, mice were anesthetized with an i.p. injection of Narcoren, transcardially perfused with 4% PFA in PBS and post-fixed in 4% PFA for 48 h at 4 °C. After this, the implant was removed, the brain dehydrated in 30% sucrose overnight at RT and cut into 30-μm sections using a sliding microtome (Hyrax S50 and KS34, Zeiss). Four series were generated for each mouse’s VMPO and one of these series was mounted and imaged with an EPI fluorescence microscope (×10, Leica, cat. no. DM6000) to assess virus expression and implant location within the MPOA. Raw recordings were first preprocessed using the Inscopix Data Processing Software (IDPS, v.1.6.0.3225). For each experimental session, video-recordings obtained at 22 and 36 °C were merged and processed together. The Timeseries module of the IDPS toolbox was used to merge the two videos. Raw imaging data were cropped to accommodate only the desired ROI and remove the lens boundary artifact. Recordings were then filtered using the spatial filter module that removes the low and high spatial frequencies (measured as the number of oscillations per pixel), thus effectively reducing out-of-focus background fluorescence. We used a trial-and-error method to identify the filter cut-off values and found that the best low cut-off value was 0.005 and the high cut-off value 0.900. The file was then motion corrected using the motion correction module of IDPS. The mean image of the video file was considered as the global reference. The maximum translational value for a pixel was also estimated using the trial-and-error method and it varied between 20 and 40 for recordings from different mice. These files were then exported as .tiff image stacks and were used to extract calcium transients using EZcalcium^92^ extraction and an analysis toolbox based on the CaImAn pipeline^93^. For this, we first performed manual ROI detection using the ROI detection module to generate the fluorescence (*ΔF*/*F*) and the deconvolved neural spiking values. The deconvolution was performed using the Markov chain Monte Carlo^94^ method, which is a fully Bayesian deconvolution method. and we used the rise and decay autoregression method to estimate the calcium indicator dynamics. We considered 0.9 as the merge threshold above which two neurons sharing a correlation coefficient would be merged into a single ROI. Calcium signals arising from each ROI were visually inspected and curated using the ROI refinement module in EZcalcium. The data were then saved as a MATLAB data (.mat) file. In addition, ROI masks were created for each file. These masks were then used for manual matching of neurons across different recording sessions. Raw *ΔF*/*F* values obtained from the EZcalcium module were then used to compute the baseline (22 °C) *z*-score (BZ), for each neuron, given by

$${\rm{BZi}}=({\mathrm{xi-xib}})/{\mathrm{sib}}$$

where xi is the fluorescence value during the baseline period, xib the mean of values from the baseline period and sib the s.d. of values from the baseline period.

Post-stimulus (36 °C) *z*-scores (PZ) were computed as a function of the BZ, given by

$${\rm{PZj}}=({\mathrm{xj-xib}})/{\mathrm{sib}}$$

where xj is the fluorescence value of a neuron after stimulus, xib the mean of values of the xj neuron from the baseline period and sib the s.d. of the xj neuron values from the baseline period. The *z*-score computation was performed using a customized Python code and was then exported as an .xlsx file for further analysis. To assess the effect of increasing ambient temperature on neuronal activity and to categorize VMPO^LepR^ neurons into WSN + WRN, CSN + CRN or insensitive cell types (Fig. 1h), we statistically compared the *z*-scores before and after increasing the ambient temperature from 22 °C to 36 °C. Specifically, we used a Kolmogorov–Smirnov test (with a *P* < 0.05 considered statistically significant) to compare the *z*-scores of each neuron at 22 °C and 36 °C. If the *z*-scores at 36 °C were significantly larger than at 22 °C, cells were classified as WSN + WRN. If the *z*-scores at 36 °C were significantly smaller than at 22 °C, cells were classified as CSN + CRN. If the *z*-scores at 36 °C were not significantly different than at 22 °C, cells were classified as temperature insensitive.

### Human brain tissue in situ hybridization

RNAscope FISH was performed on formalin-fixed, paraffin-embedded (FFPE) human brain sections covering the VMPO (tissue blocks obtained from the Edinburgh Brain Bank in collaboration with C. Smith). The tissue was sectioned at 5 μm and mounted on to Fisher SuperFrost Plus glass slides (Thermo Fisher Scientific). Multiplex FISH was performed using the Leica RX Fully Automated Research Stainer (Leica) and the RNAscope LS multiplex fluorescent reagent kit (Advanced Cell Diagnostics, Bio-Techne) with Opal fluorophore reagent pack detection (Akoya BioSciences, Inc.). All slides were counterstained with DAPI and coverslipped with ProLong Diamond antifade mountant (Thermo Fisher Scientific). The sections were hybridized with human-specific probes to detect messenger RNA transcripts for PACAP (ADCYAP1, cat. no. 582508), LEPR-tv1 (long isoform, cat. no. 410378-C2), LEPR-alltv (all isoforms, cat. no. 410388), vGLUT2 (SLC17A6, cat. no. 415678), PTGER3 (cat. no. 488438) and OPN5 (cat. no. 1058668-C2) (all from Advanced Cell Diagnostics, Bio-Techne). The slides were scanned with the Olympus VS200 slidescanner using (Olympus) a ×20 air objective (0.8 NA) and a DAPI/CY3/CY5 filter set. Images were prepared with the Olympus OlyVIA software, and signal intensity levels were adjusted to match across staining/slides.

Luxol Fast Blue (LFB) and hematoxylin and eosin (H&E) staining of myelinated fibers (blue) and cell bodies (purple) were performed on human FFPE brain sections to facilitate correct anatomical annotation. The standard staining protocol included: deparaffination, LFB (Solvent Blue 38/ethanol/acetic acid, Sigma-Aldrich) incubation overnight, followed by lithium carbonate/hematoxylin/acetic alcohol/lithium carbonate/eosin (Sigma-Aldrich/Merck) incubation steps, dehydration in xylene and mounting with Pertex.

### Data, statistical analysis and reproducibility

Data were analyzed using ImageJ (v.1.53c), Olympus OlyVIA software, R and RStudio (v.1.2.5033), Python (v.3.7.6), Microsoft Excel, Igor Pro (v.6.37), Clampfit (pClamp 11) and MATLAB (v.R2021a). Statistical tests were performed using R or GraphPad Prism (v.5.00 and v.6.00; GraphPad software). *N* numbers in each figure legend are displayed in the format ‘*n*/*N*’, with *N* being the number of mice and *n* the total number of cells recorded. Results are presented either as mean ± s.e.m. or as box plots, where the middle line represents the median, box limits represent the interquartile range (IQR) and whiskers show the minimum to maximum values. The distribution of data was assayed using the Kolmogorov–Smirnov normality test, D’Agostino and Pearson’s omnibus normality test and the Shapiro–Wilk normality test. The difference between two groups was tested using a two-sample Student’s *t*-test or the nonparametric Mann–Whitney *U*-test. For multiple group testing with analysis of variance (ANOVA) or the Kruskal–Wallis test, Tukey’s honestly significant difference, Dunn’s or Šidák’s multiple-comparison test was used as a post-hoc test.

In endoscopic imaging experiments, we used a nonparametric Kolmogorov–Smirnov test and compared the *z*-scores of individual cells at 22 °C and 36 °C to estimate whether units increased, decreased or did not change their activity on acute increase of external temperature; to statistically assess the impact of acclimation on temperature sensitivity, we compared averaged *z*-scores using two-sided Kolmogorov–Smirnov test or Wilcoxon’s signed-rank test. Values of *P* < 0.05 were considered statistically significant: ^*^P ≤ 0.05, ^**^P ≤ 0.01 and ^***^P ≤ 0.001. Details on the statistical methods applied are included in the figure legends.

In in vivo experiments, animals were excluded from analysis only if the viral injection was unsuccessful (AAV expression was not detected or was not detected in the target brain nucleus).

Data collection and analysis were not performed blind to the conditions of the experiments.

Individual data points are represented throughout all the figures.

For all ex vivo experiments, each experiment was performed at least two independent times (in most cases more than two times). For the in vivo manipulations and experiments, each experiment was at least performed two independent times.

For ex vivo recordings of neuronal firing activities, we were guided by an estimate of a minimum sample size of five cells, because this was the minimum sample size required to detect differences between acclimated and non-acclimated neurons (as per a G-power calculator); the actual number of cells used was in reality much larger than that. For other experiments, no statistical methods were used to predetermine sample sizes, but our sample sizes are similar to those reported in previous publications.

### Reporting summary

Further information on research design is available in the Nature Portfolio Reporting Summary linked to this article.

## Online content

Any methods, additional references, Nature Portfolio reporting summaries, source data, extended data, supplementary information, acknowledgements, peer review information; details of author contributions and competing interests; and statements of data and code availability are available at 10.1038/s41593-024-01830-0.

## Supplementary information

Supplementary Information Supplementary Figs. 1–5 including titles and legends. Inventory of the source data can be found on the Heidelberg University repository at the following URL: https://doi.org/10.11588/data/MRCFI2.

Reporting Summary

Supplementary Video 1 In vivo GCaMP6 imaging of VMPO^LepR^ neurons before heat acclimation. Related to Fig. 1. Representative LepR-Cre animal injected to express GCaMP6 in the VMPO. The mouse was not heat acclimated but kept at normal housing conditions (22–23 °C). During the GCaMP6 signal recording, the mouse was first kept at a temperature of 22 °C and then exposed to 36 °C as indicated in the upper part of the video. Blue ovals indicate registered cells that respond to the heat stimulus.

Supplementary Video 2 In vivo GCaMP6 imaging of VMPO^LepR^ neurons after heat acclimation. Related to Fig. 1. The same animal as shown in Supplementary Video 1 but after 30 d of heat acclimation at 36 °C. Shortly before and during the first part of the GCaMP signal recording session, the mouse was first exposed to a temperature of 22 °C and then exposed to 36 °C as indicated in the upper part of the video. Blue ovals indicate registered cells that had also responded to the heat stimulus before heat acclimation (as shown in Supplementary Video 1); red ovals indicate additional cells that responded to the heat stimulus only after heat acclimation and were not detectable in the non-acclimated mouse.

## Data Availability

The associated data are provided as source data files, with all data that are presented in Figs. 1–7 and Extended Data Figs. 1–10 (as well as Supplementary Figs. 1, 2, 4 and 5) included in subfolders named correspondingly, via the HeiData server of Heidelberg University at 10.11588/data/MRCFI2. We have made our RNA-seq data available via the publicly accessible repository Array Express (https://www.ebi.ac.uk/biostudies/arrayexpress) and the data can be accessed using the following accession no.: E-MTAB-14029. Further information and requests for resources and reagents should be directed to and will be fulfilled by the lead contact, J.S. (jan.siemens@pharma.uni-heidelberg.de).

## Code availability

The Python code used for AP waveform analysis has been deposited on GitHub and is available under the following link: https://github.com/wambroziak/Abfun.git. The MATLAB and Python codes used for endoscopic imaging data analysis can be accessed at the following GitHub link: https://github.com/AcunaLabUHD/AcunaLab_Miniscope_Siemens. Any additional information required to reanalyze the data reported in this paper is available from the lead contact upon request.
