# Supplementary material for: Thermally induced neuronal plasticity in the hypothalamus mediates heat tolerance
Source: Nat Neurosci. 2024 Dec 9;28(2):346–60. doi: 10.1038/s41593-024-01830-0 (PMC11802458; doi:10.1038/s41593-024-01830-0)
Supplement: Supplementary file 1 — Supplementary Figs. 1–5 including titles and legends. Inventory of the source data can be found on the Heidelberg University repository at the following URL: https://doi.org/10.11588/data/MRCFI2. [file 41593_2024_1830_MOESM1_ESM.pdf]

nature neuroscience

Supplementary information

https://doi.org/10.1038/s41593-024-01830-0

Thermally induced neuronal plasticity in the hypothalamus mediates heat tolerance

In the format provided by the authors and unedited

Supplementary Figure 1. Characterization of background cation currents and their effect on VMPO^{LepR} activity.

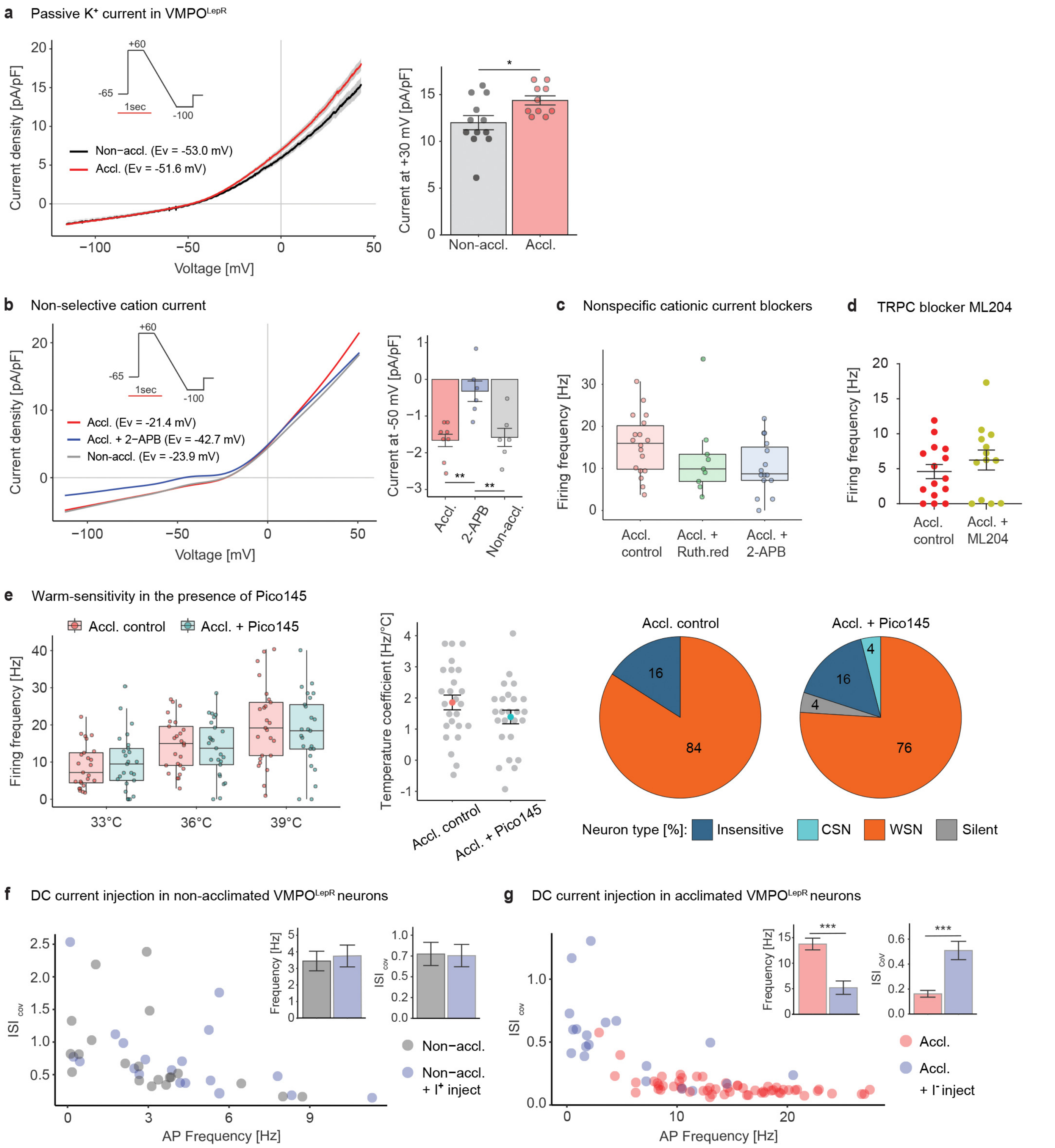

a, Left: Passive K^+ transmembrane conductance was slightly enhanced in acclimated $VMPO^{LepR}$ compared to non-acclimated controls. Average reversal potentials (E_V) are stated. Inset shows the hyperpolarising ramp protocol used. Right: Quantification of current amplitude at +30 mV based on presented traces. Unpaired two-tailed t-test, *P = 0.0208. n = 12/2 (Non-accl.) and n = 10/2 (Accl.) cells.

b, Left: Passive, nonspecific cation transmembrane current was similar between non-acclimated and acclimated $VMPO^{LepR}$ groups, but reduced by 2-APB (100 µM) in the acclimated condition. Traces represent group averages. Inset shows the hyperpolarising ramp protocol used. Right: Quantification of current amplitude at -50 mV based on presented traces. One-way ANOVA, P = 0.0016; Tukey's multiple comparison test, **P = 0.0013 (Accl. : Accl.+2-APB), **P = 0.0052 (Non-accl. : Accl.+2-APB). n = 8/1 (Accl.), n = 6/1 (Accl.+2-APB) and n = 6/1 (Non-accl.) cells.

c, Inhibition of non-selective cation currents using the non-specific TRP channel blockers Ruthenium red (10 µM) and 2-APB (100 µM) did not have a significant effect on tonic firing frequency of acclimated $VMPO^{LepR}$ neurons. n = 10/5 (Accl. control), n = 9/1 (Accl. + Ruth.red) and n = 15/2 (Accl. + 2-APB) cells.

d, Inhibition of TRPC channels with the blocker ML204 (10 µM) did not majorly affect the firing frequency of acclimated $VMPO^{LepR}$ neurons. n = 15/1 (Accl. control), n = 13/1 (Accl. + ML204) cells.

e, Blockade of TRPC1/4/5 channels did not affect the firing pattern nor warm-sensitivity of acclimation-induced $VMPO^{LepR}$ neurons. Left: Frequency of AP firing in acclimated $VMPO^{LepR}$ neurons without (red) and with the selective TRPC1/4/5 channel antagonist Pico145 (10 nM) at three bath temperatures. n = 25/5 (Accl. control), n = 25/3 (Accl. + Pico145) cells. Middle: Temperature coefficient was comparable between $VMPO^{LepR}$ acclimated control group and $VMPO^{LepR}$ neurons recorded in the presence of Pico145. Right: Distribution of temperature-insensitive, cold-sensitive (CSN), warm-sensitive (WSN) and silent neurons within acclimated $VMPO^{LepR}$ control group (n = 25/5 cells) and $VMPO^{LepR}$ group recorded in the presence of Pico145 (25/3 cells).

f, Depolarization of non-acclimated $VMPO^{LepR}$ neurons to approximate average membrane potential of acclimated $VMPO^{LepR}$ neurons (-45 mV, requiring the injection of current I+ between +20 pA to +70 pA) did not lead to an increase of firing frequency or increased regularity of firing (represented by the coefficient of variation of interspike interval, ISI_{CoV}). n = 22/8 (Non-accl.) and n = 20/2 (Non-accl. + I+) cells.

g, Hyperpolarisation of acclimated $VMPO^{LepR}$ neurons to match the average membrane potential of non-acclimated $VMPO^{LepR}$ neurons (approx. -55 mV, requiring the injection of current I- between -50 to -100 pA) decreased both firing frequency and regularity of firing. Unpaired two-tailed t-test, ***P = 0.0001 for both AP frequency and ISI_{CoV}. n = 20/7 (Accl.) and n = 20/2 (Accl. + I-) cells.

Brain slice recordings were conducted at 36°C bath temperature (unless indicated otherwise). In panel (e), "high-K^+ aCSF" and synaptic blockers were used. In panel (d), "low-K^+ aCSF" and 33°C bath temperature were used.

All barplots represent mean ± s.e.m. Boxplots represent median and interquartile range.

Supplementary Figure 2. Characterization of action potential waveform and active, voltage-gated conductances in VMPO^{LepR} neurons.

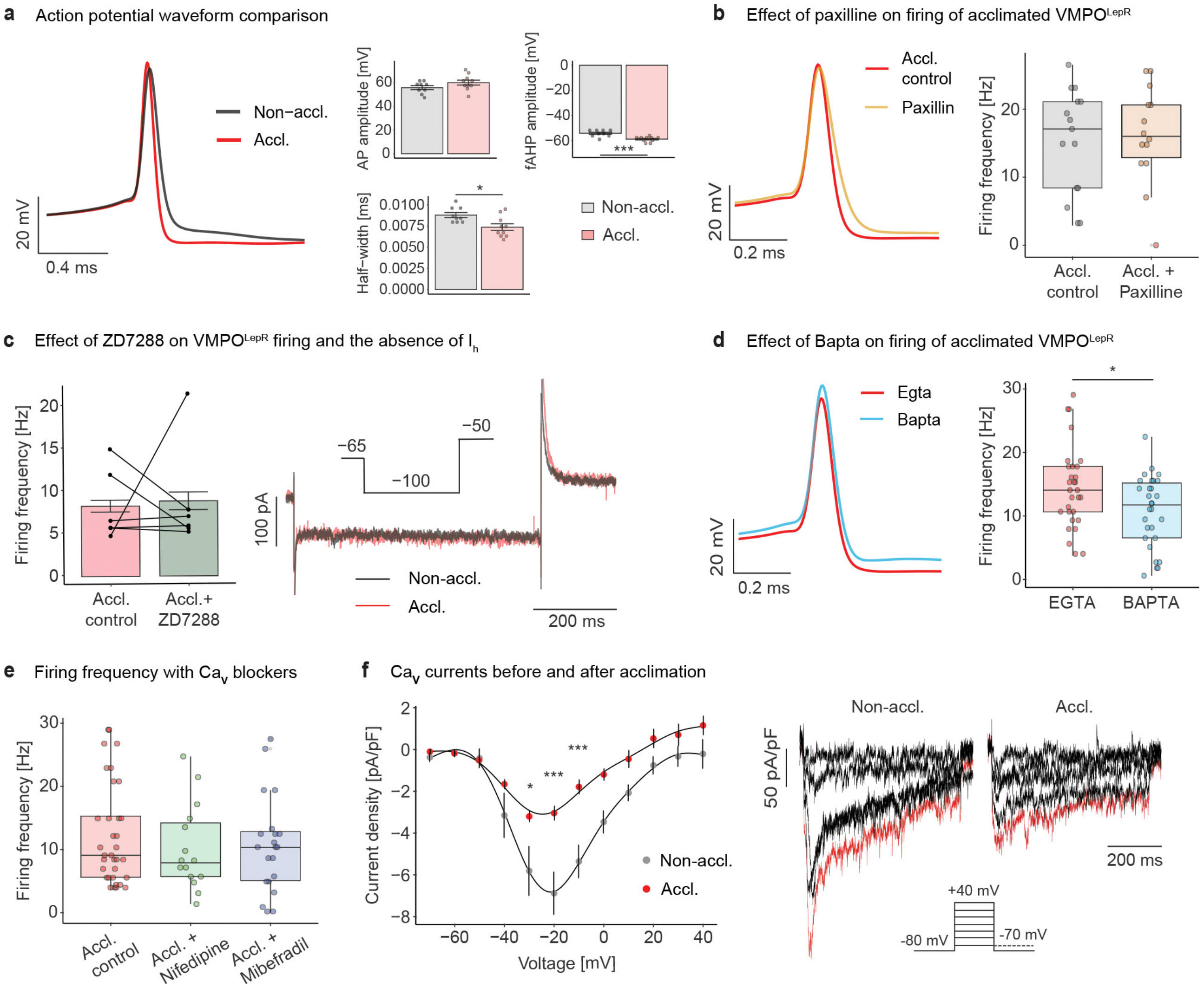

a, Left: Alignment of averaged action potential waveforms recorded from non-acclimated (n = 9/4 cells) and acclimated (n = 10/5 cells) $VMPO^{LepR}$ neurons. Right: Average amplitude of action potentials was similar between non-acclimated and acclimated $VMPO^{LepR}$ neurons. AP half-width was found to be shorter in $VMPO^{LepR}$ cells after acclimation (Unpaired two-tailed t-test, *P = 0.0109). Fast afterhyperpolarization (fAHP) was found to have a significantly larger amplitude in the acclimated condition compared to non-acclimated controls (Unpaired two-tailed t-test, ***P = 0.0005). Barplots represent mean ± s.e.m.
b, Left: Paxilline (10 μM) reduced fast AHP in acclimated $VMPO^{LepR}$ neurons (yellow trace, n = 5/1 cells, averaged and aligned AP waveforms are shown). Right: Despite reduced fAPH, Paxilline had no impact on the frequency of AP firing in acclimated $VMPO^{LepR}$ cells. n = 15/9 cells for Accl. control and n = 15/1 cells for Accl. + Paxilline.
c, Left: HCN channel blocker ZD7288 did not affect firing frequency in acclimated $VMPO^{LepR}$ neurons (n = 6, paired recordings). Barplots represent mean ± s.e.m. Right: Example traces recorded from a non-acclimated and acclimated $VMPO^{LepR}$ cell using a hyperpolarising step protocol (shown in inset).
d, Left: Exchanging EGTA for BAPTA in the pipette solution had a reducing effect on the AHP in acclimated $VMPO^{LepR}$ cells (blue trace, n = 6/2 cells; averaged and aligned AP waveforms are shown). Right: BAPTA slightly reduced the frequency of firing in acclimated $VMPO^{LepR}$ neurons (Unpaired two-tailed t-test, *P = 0.0230; n = 30/10 cells for EGTA and n = 29/4 cells for BAPTA).
e, Ca_V channel blockers Nifedipine (10 μM) and Mibefradil (1 μM) did not significantly alter AP firing frequency of acclimated $VMPO^{LepR}$ neurons (n = 17/8 cells for Accl. control, n = 15/2 cells for Accl. + Nifedipine and 21/2 for Accl. + Mibefradil).
f, Voltage-gated Ca^{2+} (Ca_V) currents recorded in non-acclimated and acclimated $VMPO^{LepR}$ neurons suggested the overall Ca_V density to be reduced in acclimated $VMPO^{LepR}$ compared to non-acclimated controls. Left: averaged (± s.e.m.) Ca_V current-voltage relationship obtained for acclimated and non-acclimated $VMPO^{LepR}$ neurons. Two-way ANOVA (effect of acclimation + voltage), P < 0.0001; Tukey's multiple comparison test, *P = 0.0342 (-30 mV), ***P < 0.0001 (-20 mV), ***P = 0.0001 (-10 mV). n = 7/2 (Non-accl.) and n = 10/2 (Accl.) cells. Right: Example traces of recorded Ca_V currents; inset: voltage step protocol used.
Brain slice recordings were conducted at 36°C bath temperature with the exception of panel (c) where "low-K^+ aCSF" and 33°C bath temperature were used.
All boxplots represent median and interquartile range.

Supplementary Figure 3. Histological Validation of AAV-Cre expression in $Na_V1.3^{fl/fl}$ and wildtype mice.

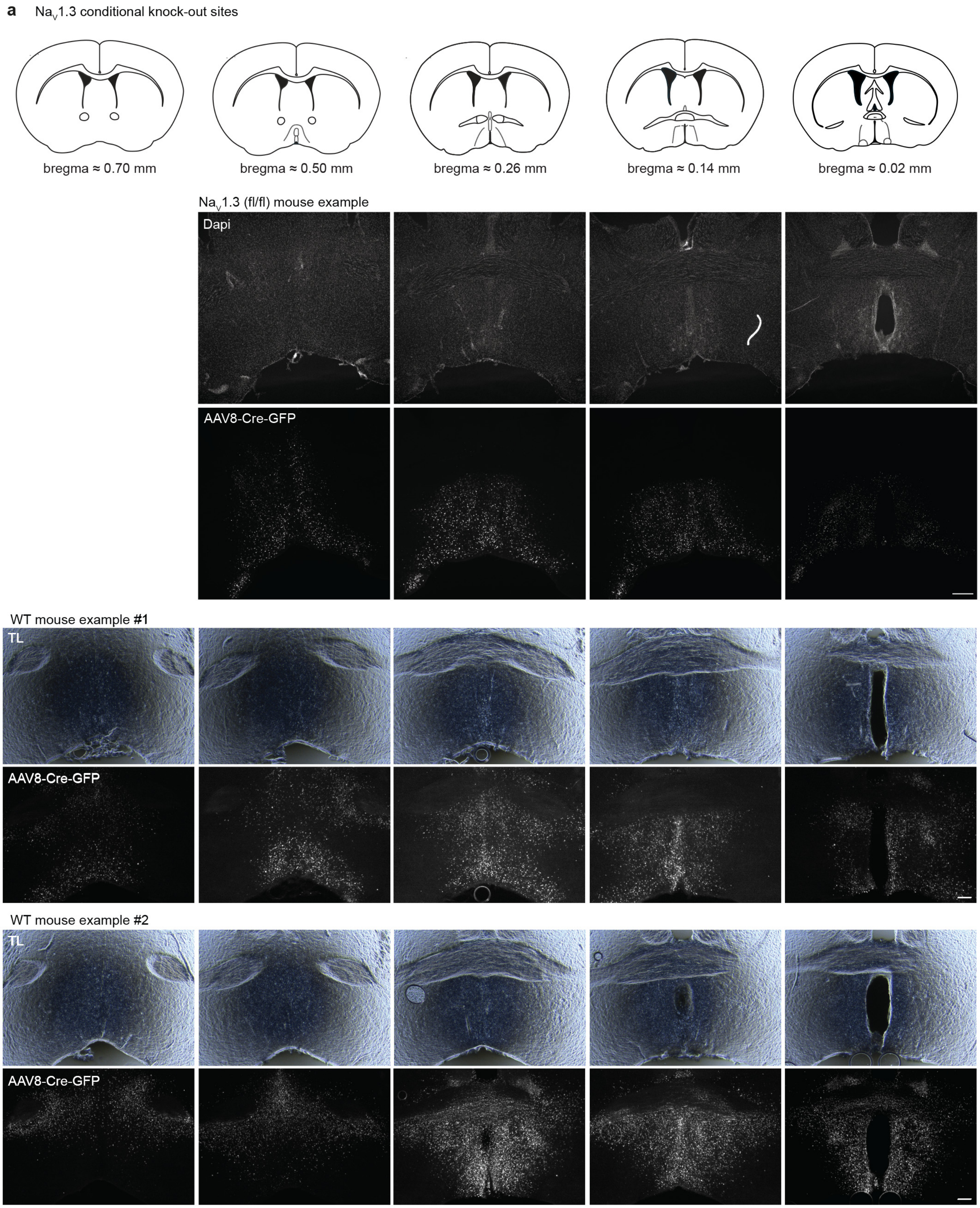

a, Images showing the site of virally-delivered Cre recombinase by GFP expression in $Na_V1.3^{fl/fl}$ and wildtype control mice. Cartoons on top show antero-posterior coordinates of the mouse brain, reflecting the stereotactic injection sites.

For wildtypes the DIC images are shown above the fluorescent images (TL = transmitted light); Scale bars = 100 μm.

Supplementary Figure 4. Verification and characterization of $Na_v1.3$ conditional Knock-out.

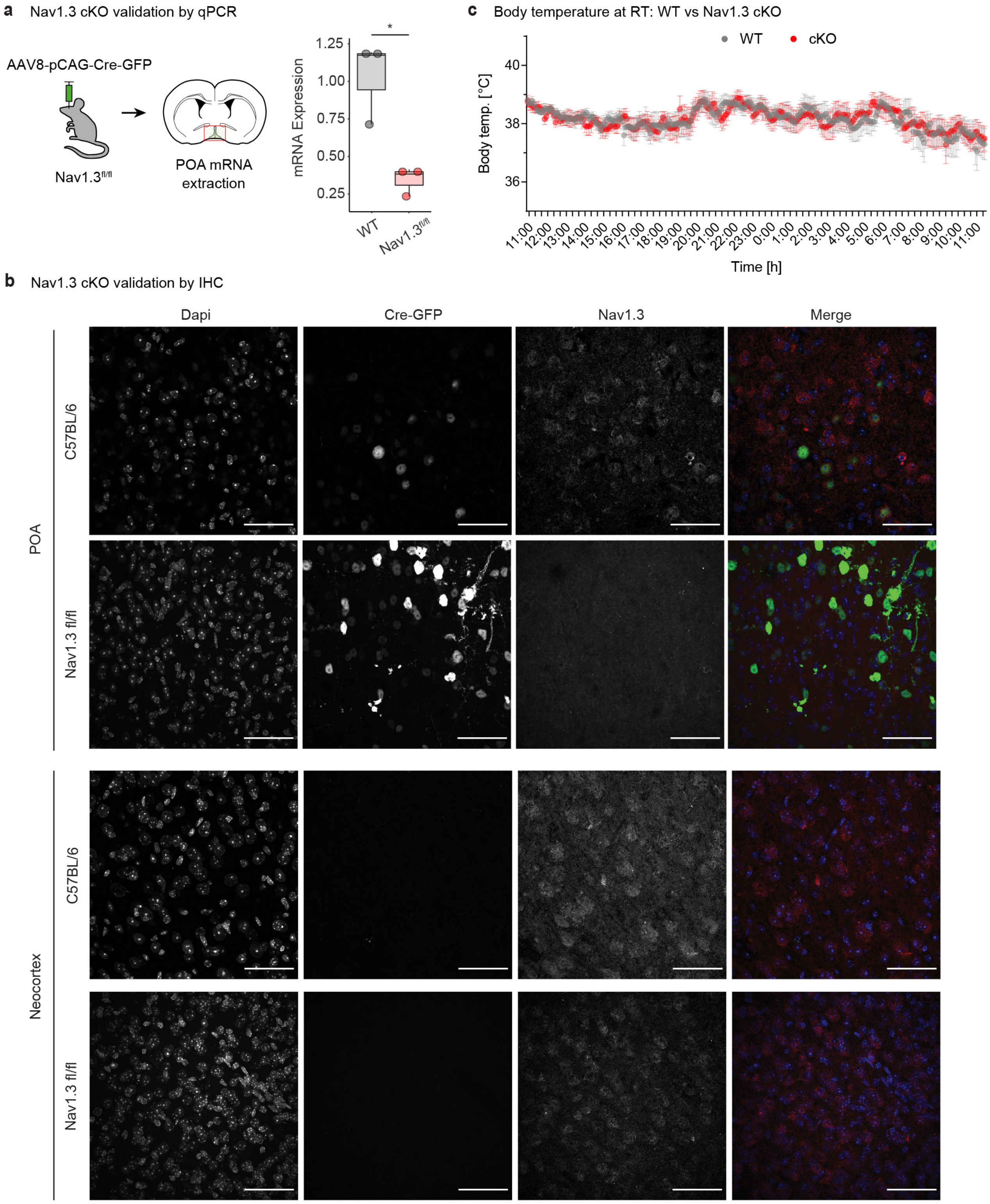

a, Left: in order to test the efficacy of Scn3a conditional knock-out (cKO), Cre recombinase-carrying AAVs were injected into the POA of Nav1.3$^{fl/fl}$ mice and C57BL/6 wildtype (WT) mice. Following 3 weeks of virus expression, mRNA was extracted from the POA tissue. Right: boxplot (median and interquartile range) showing qPCR to assess relative Na_V1.3 mRNA expression normalized to the housekeeping genes Tubb3 and Ube2l3. Unpaired two-tailed t-test, *P = 0.0147. N = 3 Cre-injected wildtype (WT) and N = 3 Cre-injected Nav1.3$^{fl/fl}$ (NaV1.3 cKO) mice.

b, Average (mean ± s.e.m.) body temperature measured telemetrically of Nav1.3 cKO and control mice at room temperature. N = 9 mice.

c, Immunohistochemistry in brain slices using antibodies detecting GFP and Na_v1.3 and counterstained with DAPI. Merged images show GFP in green and Na_v1.3 in red. 60x images show staining signal in POA and neocortex of AAV-Cre injected C57BL/6 wildtype (WT) and AAV-Cre injected Na_V1.3$^{fl/fl}$ (Na_V1.3 cKO) mice. Shown are representative images of mice stereotaxically injected with AAV8-Cre-GFP into the POA (but not into the neocortex, a brain region that therefore served as internal control). Scale bar = 50 μm.

Supplementary Figure 5. Human VMPO expression analyses of "QPLOT" marker genes LEPR, vGLUT2, PACAP, PTGER3 and OPN5.

a Human tissue block covering VMPO - expression of PACAP

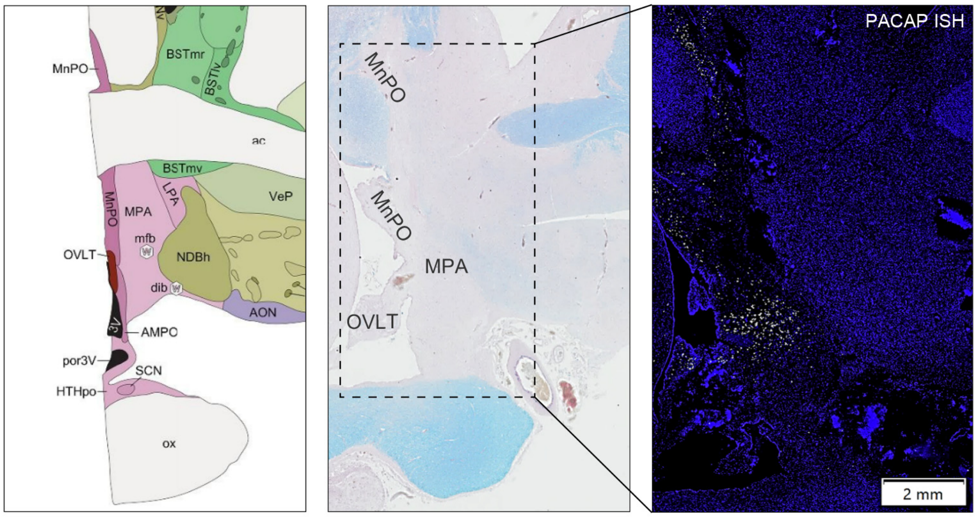

b Overlap of LEPR isoforms with PACAP and vGLUT2 in human VMPO

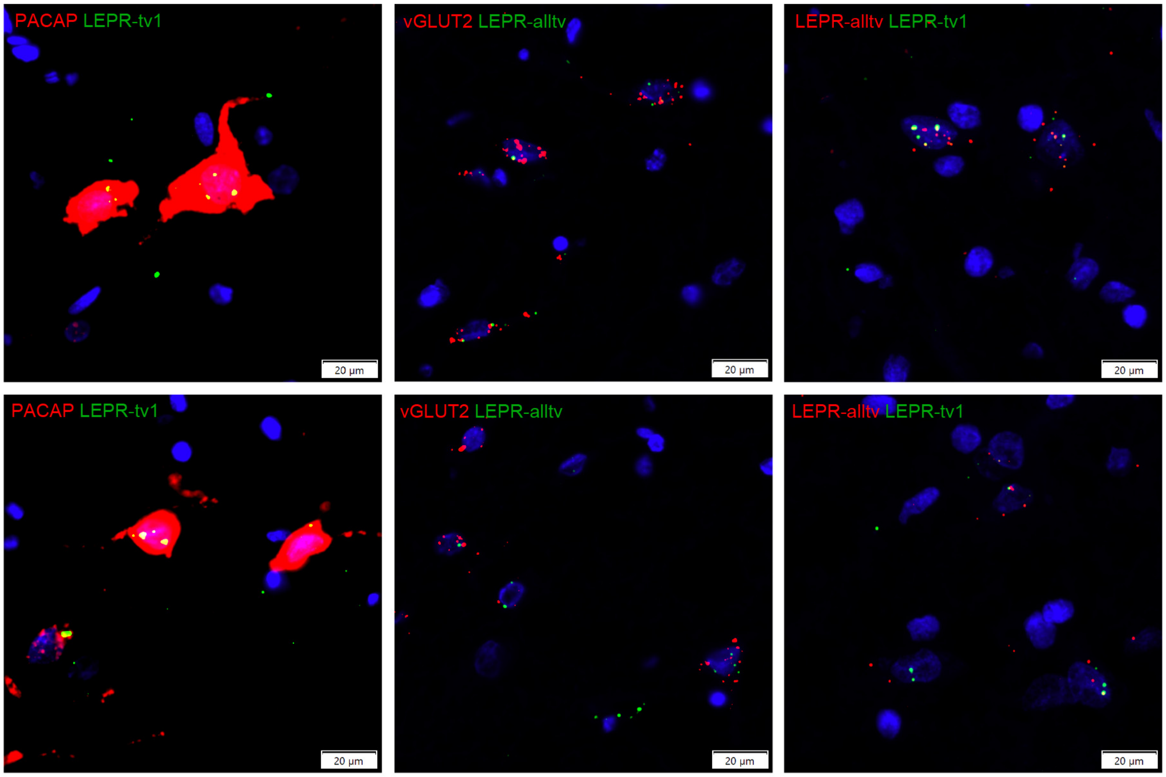

c Overlap of PTGER3 and OPN5 in human VMPO

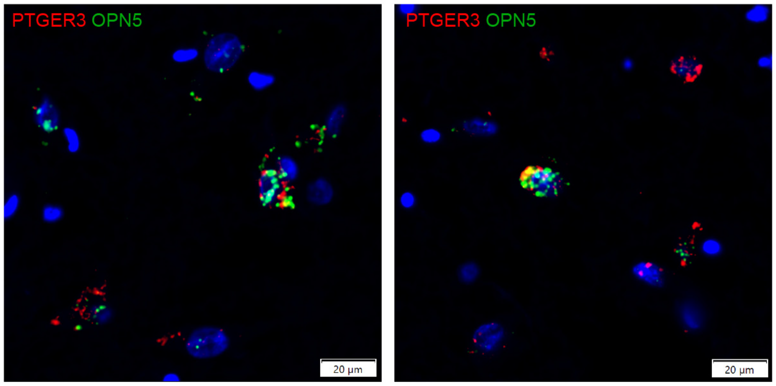

a, Left: Allen Brain Atlas annotation of human VMPO. Middle: human tissue block covering preoptic areas MnPO/MPA/OVLT (LFB/HE stain). Right: ISH of the VMPO brain section showed in the middle panel stained for PACAP (ADCYAP1).
b, Additional examples of RNAscope ISH showing LEPR co-expression in human VMPO. Two different LEPR ISH probes (see Methods) were co-labeled with PACAP (ADCYAP1) and vGLUT2 (SLC17A6). Co-expression of mRNA is shown in yellow.
c, RNAscope ISH showing co-expression (yellow) of other thermoregulatory neuronal markers, the Prostaglandin E Receptor 3 (PTGER3) and Opsin 5 (OPN5), in human VMPO.
Images were taken from 1 tissue section/staining from a single human donor.

Source data - inventory
to be found at the following address: https://doi.org/10.11588/data/MRCFI2

Main Figures:

Source data Fig 1
Statistical source data for Fig 1b-e.

Source data Fig 2
Statistical source data for Fig 2a,b.

Source data Fig 3
Statistical source data for Fig 3b,c,e,f.

Source data Fig 4
Statistical source data for Fig 4c.

Source data Fig 5
Statistical source data for Fig 5b,c.

Source data Fig 6
Statistical source data for Fig 6a-k.

Source data Fig 7
Statistical source data for Fig 7c,d,e,g,h and image data for Fig 7i,j.

Extended Data Figures:

Source data Ext Data Fig 1
Statistical source data for Ext Data Fig 1a,b,d,e and image data for Ext Data Fig 1a,b,e.

Source data Ext Data Fig 2
Statistical source data for Ext Data Fig 2a-j.

Source data Ext Data Fig 3_miniscope
Statistical source data for Ext Data Fig 3_miniscope d-h.

Source data Ext Data Fig 4
Statistical source data for Ext Data Fig 4a-i.

Source data Ext Data Fig 5
Statistical source data for Ext Data Fig 5b,c.

Source data Ext Data Fig 6
Statistical source data for Ext Data Fig 6a,b,c,f,g,h,i.

Source data Ext Data Fig 7

Statistical source data for Ext Data Fig 7a,c-i.

Source data Ext Data Fig 8
Statistical source data for Ext Data Fig 8b-h.

Source data Ext Data Fig 9
Statistical source data for Ext Data Fig 9 a,b,c,d,e,g,h,i.

Source data Ext Data Fig 10
Statistical source data for Ext Data Fig 10a-d.

Supplementary Figures:

Source data Suppl Fig 1
Statistical source data for Suppl Fig 1a-g.

Source data Suppl Fig 2
Statistical source data for Suppl Fig 2a-f.

Source data Suppl Fig 4
Statistical source data for Suppl Fig 4a,c.

Source data Suppl Fig 5
Image data for Suppl Fig 5a,b.
