# Supplementary material for: Thermally induced neuronal plasticity in the hypothalamus mediates heat tolerance
Source: Nat Neurosci. 2024 Dec 9;28(2):346–60. doi: 10.1038/s41593-024-01830-0 (PMC11802458; doi:10.1038/s41593-024-01830-0)
Supplement: Supplementary file 2 — Reporting Summary [file 41593_2024_1830_MOESM2_ESM.pdf]

nature portfolio

Corresponding author(s): Jan Siemens

Last updated by author(s): 20 Oct 2024

Reporting Summary

Nature Portfolio wishes to improve the reproducibility of the work that we publish. This form provides structure for consistency and transparency in reporting. For further information on Nature Portfolio policies, see our Editorial Policies and the Editorial Policy Checklist.

Statistics

For all statistical analyses, confirm that the following items are present in the figure legend, table legend, main text, or Methods section.

n/a	Confirmed	
☐	☒	The exact sample size (*n*) for each experimental group/condition, given as a discrete number and unit of measurement
☐	☒	A statement on whether measurements were taken from distinct samples or whether the same sample was measured repeatedly
☐	☒	The statistical test(s) used AND whether they are one- or two-sided<br>*Only common tests should be described solely by name; describe more complex techniques in the Methods section.*
☒	☐	A description of all covariates tested
☐	☒	A description of any assumptions or corrections, such as tests of normality and adjustment for multiple comparisons
☐	☒	A full description of the statistical parameters including central tendency (e.g. means) or other basic estimates (e.g. regression coefficient) AND variation (e.g. standard deviation) or associated estimates of uncertainty (e.g. confidence intervals)
☐	☒	For null hypothesis testing, the test statistic (e.g. *F*, *t*, *r*) with confidence intervals, effect sizes, degrees of freedom and *P* value noted<br>*Give P values as exact values whenever suitable.*
☒	☐	For Bayesian analysis, information on the choice of priors and Markov chain Monte Carlo settings
☒	☐	For hierarchical and complex designs, identification of the appropriate level for tests and full reporting of outcomes
☒	☐	Estimates of effect sizes (e.g. Cohen's *d*, Pearson's *r*), indicating how they were calculated

Our web collection on statistics for biologists contains articles on many of the points above.

Software and code

Policy information about availability of computer code

Data collection	pClamp 10/11 (Molecular Devices, USA), Ponemah (DSI, USA), IRBIS 3 (InfraTec, Germany), Olympus OlyVIA
Data analysis	Clampfit 10/11 (pClamp package, Molecular Devices, USA), Rstudio (version 1.2.5033), GraphPad Prism (V5.00 and V6.00; GraphPad software, USA), Python (V3.7.6, custom code was described and made available via link specified in Methods), Igor Pro (V6.37), IRBIS 3 (InfraTec, Germany), Fiji (ImageJ v1.53c), MATLAB (custom code was described and made available via link specified in Methods)

For manuscripts utilizing custom algorithms or software that are central to the research but not yet described in published literature, software must be made available to editors and reviewers. We strongly encourage code deposition in a community repository (e.g. GitHub). See the Nature Portfolio guidelines for submitting code & software for further information.

Data

Policy information about availability of data

All manuscripts must include a data availability statement. This statement should provide the following information, where applicable:

- Accession codes, unique identifiers, or web links for publicly available datasets
- A description of any restrictions on data availability
- For clinical datasets or third party data, please ensure that the statement adheres to our policy

The associated data are provided as Source Data Files, with all data that is presented in each main and Extended Data figure included in subfolders named

correspondingly in the Source Data folder found on the HeiData server of Heidelberg University (https://heidata.uni-heidelberg.de/) at the following address: https://doi.org/10.11588/data/MRCFI2
We have made our RNAseq data available via the publicly accessible repository Array Express (https://www.ebi.ac.uk/biostudies/arrayexpress) and the data can be accessed using the following entry ID: E-MTAB-14029.
The Python code used for action potential waveform analysis has been deposited on Github and is available under following link: https://github.com/wambroziak/Abfun.git
The MATLAB and Python codes used for endoscopic imaging data analysis can be accessed at the following Github link: https://github.com/AcunaLabUHD/AcunaLab_Miniscope_Siemens
Any additional information required to reanalyze the data reported in this paper is available from the lead contact upon request.

Research involving human participants, their data, or biological material

Policy information about studies with human participants or human data. See also policy information about sex, gender (identity/presentation), and sexual orientation and race, ethnicity and racism.

Reporting on sex and gender	N/A
Reporting on race, ethnicity, or other socially relevant groupings	N/A
Population characteristics	N/A
Recruitment	N/A
Ethics oversight	N/A

Note that full information on the approval of the study protocol must also be provided in the manuscript.

Field-specific reporting

Please select the one below that is the best fit for your research. If you are not sure, read the appropriate sections before making your selection.

☒ Life sciences ☐ Behavioural & social sciences ☐ Ecological, evolutionary & environmental sciences

For a reference copy of the document with all sections, see nature.com/documents/nr-reporting-summary-flat.pdf

Life sciences study design

All studies must disclose on these points even when the disclosure is negative.

Sample size	For electrophysiology experiments where the frequency of action potentials was measured, the minimum sample size based on the effect size calculated was 5 cells. Sample size calculations were performed with the help of G*Power 3.1 software. For other electrophysiological recordings as well as in vivo behavioral experiments sample size was determined based on comparable literature data.
Data exclusions	For in vivo experiments, dataset with faulty telemetry readings (due to failure of implantable transmitters) and where an incorrect implantation/injection site was confirmed post-hoc with immunohistochemistry were excluded. In electrophysiological voltage clamp experiments, cell recordings where membrane resistance or series resistance changed considerably during experiment (>50% and >20% respectively) were excluded from analysis. In all experiments, the animal genotype was assessed before allocating them into groups and verified after concluding the experiment; when genotype was mistaken, the animal was excluded from the experiment or (when possible) allocated retrospectively to the correct group.
Replication	Electrophysiological experiments were usually done with cell recordings from at least two mice with few exceptions as indicated in the appropriate figure legends. Minimum number of cells recorded was 5 per condition. In vivo behavioral experiments were conducted according to comparable literature data (minimum 4 animals per group). At least two rounds of testing were conducted per type of experiment to verify that the results were reproducible. Number of cells measured ex vivo is indicated in the figure legends as n = x/X, meaning x number cells from X number of animals. Number of animals tested behaviorally per each experiment is indicated in the figure legends as N = X).
Randomization	Mice of all genotypes and both sexes were randomly chosen from core colonies to allocate them into experimental groups.<br>Cells recorded ex vivo were chosen randomly as long as their cell membrane appeared healthy and they occupied the brain area of interest.
Blinding	Investigators were not blinded to mice genotypes.

Reporting for specific materials, systems and methods

We require information from authors about some types of materials, experimental systems and methods used in many studies. Here, indicate whether each material, system or method listed is relevant to your study. If you are not sure if a list item applies to your research, read the appropriate section before selecting a response.

Materials & experimental systems

n/a	Involved in the study
	☒ Antibodies
☒	☐ Eukaryotic cell lines
☒	☐ Palaeontology and archaeology
	☒ Animals and other organisms
☒	☐ Clinical data
☒	☐ Dual use research of concern
☒	☐ Plants

Methods

n/a	Involved in the study
☒	☐ ChIP-seq
	☒ Flow cytometry
☒	☐ MRI-based neuroimaging

Antibodies

Antibodies used

Chicken anti-GFP (Novus Bio, NB100-1614), Rabbit anti-c-Fos (Synaptic Systems, 226003) Rabbit anti-mCherry (abcam, ab167453), rabbit anti-SCN3A (abcam, ab65164), Goat anti-Chicken IgY secondary antibody + Alexa Fluor 488 (Invitrogen, A-11039), Donkey anti-Rabbit IgG secondary andtibody + Alexa Fluor 555 (Invitrogen, A-21430)

Validation

Chicken anti-GFP: https://www.novusbio.com/products/gfp-antibody_nb100-1614; Rabbit anti-c-Fos (polyclonal) validated for use in several species, including mouse and rat for WB, ICC and IHC as per manufacturer's datasheet https://pim.sysy.com/product/226308; Rabbit anti-mCherry: https://www.abcam.com/products/primary-antibodies/mcherry-antibody-ab167453.html; rabbit anti-SCN3A validated for use in human samples for WB, ICC and IHC (see https://www.abcam.com/products/primary-antibodies/scn3a-antibody-ab65164.html); rabbit anti-SCN3a was also validated for mouse tissue (IHC) in our manuscript, see Extended Data Fig. 14.

Animals and other research organisms

Policy information about studies involving animals; ARRIVE guidelines recommended for reporting animal research, and Sex and Gender in Research

Laboratory animals

Mice (mus musculus) of both sexes were used for experiments. Lines used: LepR-cre: LRb stop IRES Cre 3’UTR neo; HTB.DFrt: Rosa26 CAG LoxP PGK-neo Stop LoxP FRT H-GFP 2A TVA 2A B19G; Vgat-Flpo: B6.Cg-Slc32a1tm1.1(flpo)Hze/J; TrpV1-cre: B6.129-Trpv1tm1(cre)Bbm/J (TRPV1-cre); Rosa-DTA: Gt(ROSA)26Sortm1(DTA)Jpmb/J; Vglut2-cre: STOCK Slc17a6tm2(cre)Lowl/J; FosTRAP2: Fostm2.1(icre/ERT2)Luo/J; Nav1.3-floxed: B6.129S6-Scn3atm1.1Jwo/H; PACAP-EGFP: Tg(Adcyap1-EGFP)FB22Gsat/Mmucd

Wild animals

No wild animals were used in this study.

Reporting on sex

Animals of both sexes were used in this study for both ex vivo and in vivo recordings. However, no sex-specific analyses were performed since the study did not focus on sex-dependent nuances of temperature regulation, and no apparent differences were observed with respect to the effects studied.

Field-collected samples

Study did not contain samples collected from the field.

Ethics oversight

All experiments involving animals were approved by the local authorities (Regierungspräsidium Karlsruhe), under animal protocol numbers G-111/14, G-168/15, G-169/18, G-223/18 and G-181/21.

Note that full information on the approval of the study protocol must also be provided in the manuscript.

Plants

Seed stocks

N/A

Novel plant genotypes

N/A

Authentication

N/A

Flow Cytometry

Plots

Confirm that:

☐ The axis labels state the marker and fluorochrome used (e.g. CD4-FITC).

☐ The axis scales are clearly visible. Include numbers along axes only for bottom left plot of group (a 'group' is an analysis of identical markers).

☐ All plots are contour plots with outliers or pseudocolor plots.

☐ A numerical value for number of cells or percentage (with statistics) is provided.

Methodology

Sample preparation	Micro-dissected POA brain tissue of LepR-Cre;HTB-GFP mice that were subjected to heat acclimation for various time periods. Dispersion of cells and preparation of samples is explained in detail in the methods section of the manuscript.
Instrument	BD FACSAria III
Software	BD FACSDiva Software v7
Cell population abundance	abundance of GFP-positive cells was between 0.2% and 0.8% of all cells alive (dead cells were excluded based on propidium iodid/PI labelling)
Gating strategy	The target cell population was gated using FSC/SSC (Gate1), then dead cells (Subgate2) were excluded via propidium iodide (PI) labeling. Because the target cell population was very small, a high number of absolute cells (approximately 500,000) were sorted to establish a meaningful GFP+/GFP- gate for sorting. All gates were set as dot plot (not contour plot). For the GFP gate, PI was set as the second parameter.

☐ Tick this box to confirm that a figure exemplifying the gating strategy is provided in the Supplementary Information.
